# Precision medicine in nasopharyngeal carcinoma: comprehensive review of past, present, and future prospect

**DOI:** 10.1186/s12967-023-04673-8

**Published:** 2023-11-06

**Authors:** Pui Yan Siak, Win Sen Heng, Sharon Siew Hoon Teoh, Yu Yu Lwin, Shiau-Chuen Cheah

**Affiliations:** 1 Faculty of Medicine and Health Sciences, UCSI University, Bandar Springhill, 71010 Port Dickson, Negeri Sembilan Malaysia; 2 Department of Otorhinolaryngology-Head and Neck Surgery, University of Medicine, Mandalay, Myanmar

**Keywords:** Nasopharyngeal carcinoma, Personalized medicine, Epstein–Barr virus, Cancer stem cell, Metastasis, Molecular heterogeneity, Targeted therapy, Genomic profiling, Precision oncology

## Abstract

Nasopharyngeal carcinoma (NPC) is an aggressive malignancy with high propensity for lymphatic spread and distant metastasis. It is prominent as an endemic malignancy in Southern China and Southeast Asia regions. Studies on NPC pathogenesis mechanism in the past decades such as through Epstein Barr Virus (EBV) infection and oncogenic molecular aberrations have explored several potential targets for therapy and diagnosis. The EBV infection introduces oncoviral proteins that consequently hyperactivate many promitotic pathways and block cell-death inducers. EBV infection is so prevalent in NPC patients such that EBV serological tests were used to diagnose and screen NPC patients. On the other hand, as the downstream effectors of oncogenic mechanisms, the promitotic pathways can potentially be exploited therapeutically. With the apparent heterogeneity and distinct molecular aberrations of NPC tumor, the focus has turned into a more personalized treatment in NPC. Herein in this comprehensive review, we depict the current status of screening, diagnosis, treatment, and prevention in NPC. Subsequently, based on the limitations on those aspects, we look at their potential improvements in moving towards the path of precision medicine. The importance of recent advances on the key molecular aberration involved in pathogenesis of NPC for precision medicine progression has also been reported in the present review. Besides, the challenge and future outlook of NPC management will also be highlighted.

## Introduction

Nasopharyngeal carcinoma (NPC) is an aggressive malignancy with significant percentage of patients developed distant metastasis [1]. It is arisen in mucosal epithelium of the nasopharynx, frequently found in the pharyngeal recess and exhibits remarkable ethnic and distinct geographic distribution [2]. This cancer is highly prevalent with 70% of total cases found in Southeast Asia, South China, Northern Africa, Greenland, and Alaska but remains incredibly rare in western countries [3]. With the fact that NPC is highly prevalence in Guangdong Province in Southern China in the early twentieth century, it is also being known as the “Guangdong cancer” [4, 5]. According to the International Agency for Research on Cancer (IARC), approximately 133,354 new cases of NPC and 80,008 of mortality were reported worldwide in 2020 [6]. The annual incidence rate in endemic region is between 10 and 50 per 100,000 persons, which is up to 50 times higher than the incidence in the western countries with a rate of 1 per 100,000 persons [7]. Meanwhile, according to Global Cancer Observatory (GLOBOCAN) 2020, the world age-standardized rate (ASR) was reported at 2.2 per 100,000 for males and 0.8 per 100,000 for females (data is retrieved as of October 2023). The peak age of disease occurrence is 45 years old [2]. According to the World Health Organization (WHO), NPC is categorized into 3 sub-types based on the distinct histopathological characteristics observed under the light microscope, i.e., keratinizing squamous cell carcinoma (type I), non-keratinizing squamous cell carcinoma (type II), and basaloid squamous carcinoma (type III). Type II NPC is further sub-classified into undifferentiated or differentiated subtype [8]. Based on this classification, it is clear that NPCs are of squamous cell origin. In fact, type II NPC comprises up to 95% of incidence in the endemic areas with annual peak of 30 per 100,000 persons, while type I NPC is more common in the western countries (United States and Europe) with up to 75% of cases [9]. Non-keratinizing NPC is more likely to display metastatic disease albeit with better therapeutic response compared to the keratinizing NPC which is associated with poorer prognosis and locally advanced disease development [8].

Genetic susceptibility and Epstein Barr Virus (EBV) infection are the two prominent etiological factors that are closely implicated in malignancy transformation and oncogenesis of NPC. Notwithstanding, migrants from Southern China living in non-endemic areas are still associated with high NPC susceptibility [10]. Therefore, it is suggested that hereditary predisposition is one of the most important risk factors. Various NPC susceptible loci and gene mutations were already reported [11–15]. Notably, men are having 2–3 times higher risk of NPC compared to women. This is probably due to the genetic difference between male and female in X-chromosome or oestrogen sex hormone, or a combination of these factors [16, 17]. Certain genetic susceptibilities, such as chromosomal alterations and human leukocyte antigen (HLA) polymorphism, have been found to be associated with high risk of NPC. For example, Chinese and other Asians with HLA-A2, -B-46, -B17 and Caucasians with HLA-B5 were reported to have a higher (~ twofold) risk of developing NPC [18, 19]. The loss of chromosomes 3p and 9p, as well as copy number increases on chromosome 12, were also associated with a high risk of NPC [20, 21]. Besides that, studies of NPC genetic landscape have allowed the identification of numerous NPC oncogenic molecular aberrations. Recent study by Xiao team has discovered germline mutations in *DNA polymerase nu* (*POLN*) gene (*P577L*, *R303Q*, and *F545C*) were highly susceptible to NPC development [22]. A whole-genome mutation analysis study has revealed numerous oncogenic mutations in the nuclear factor kappa B (NF-κB) pathway [12]. Meanwhile, these genetic aberrations could be triggered or further promoted by EBV infection. EBV-associated oncoviral protein has been known with its significant roles in aberrantly inducing the intracellular signalling, cytokines, or chemokines release in NPC’s tumor microenvironment (TME), leading to abnormal proliferation, immune escape, and acquisition of NPC invasive nature and metastatic features [23]. EBV infection is thought to be a critical etiological factor for the local prevalence of non-keratinizing NPC pathogenesis. However, considering that a minor group of NPC patients were not EBV infected, viral infection alone is insufficient to justify the cause of this disease. Therefore, epidemiologic factors including alcohol drinking, consumption of salt-preserved food, cigarette smoking, environmental exposure, and lifestyle have also contributed to NPC pathogenesis, especially in the keratinizing NPC [24, 25]. Researchers have found that people living in the endemic region generally consume heavily salted preserved meat, vegetables, fish and pickled foods [26]. These foods are known to contain high levels of nitrates and nitrites, which can lead to the formation of nitrosamines that damage DNA and contribute to carcinogenesis [27]. In addition, numerous studies in southern China have demonstrated a significantly increase in the incidence rate of NPC associated with the consumption of salted fish and vegetables [27–30]. Furthermore, a study has described a possible interaction between genetic factors (family history of cancer) and environmental factors (consumption of salt-preserved fish) in driving NPC development. However, the mechanism of this interaction that drives carcinogenesis remains to be explored. There is a relevant study that demonstrated a polymorphism of cytochrome P450 family 2 subfamily E member 1 (CYP2E1), which is associated with NPC susceptibility, and it was found to upregulate the activation of pro-carcinogens, including nitrosamines that found in tobacco, salted and preserved food [31]. Therefore, the combination of genetic polymorphism of CYP2E1 and diet high in preserved foods or tobacco smoking could further increase the susceptibility to NPC development.

With the advancement of radiotherapy technology, wide variety of chemotherapy application, and disease staging system, overall prognosis has been improved over the past three decades. The different stages of NPC are determined based on the tumor, node, and metastasis (TNM) system which is also a fundamental system for treatment decisions [32]. Current treatment has excellent control with a good prognosis up to 90% for the early stage NPC, but the treatment outcomes in advanced NPC remain disappointing [33]. The overall survival (OS) of NPC patients is still unsatisfatory and this is mainly due to: (1) the emergence of radio- or chemo-resistance, (2) the development of distant metastasis or disease recurrence after radiotherapy, and (3) the fatal toxicity of salvage radiotherapy and concurrent chemotherapy in advanced stage of NPC [9]. Besides that, due to asymptomatic characteristic of early stage NPC, more than 90% of NPC patients were initially diagnosed with advanced stage, thereby limiting the choice of treatment and leading to higher risk of disease recurrence and development of distant metastasis [7]. Hence, it is crucial to develop effective therapeutics, early diagnosis strategies, as well as prognostic approaches. Recent focus has shifted towards targeted therapy and immunotherapy for NPC treatment, such as inhibitors of epidermal growth factor receptor (EGFR), vascular endothelial growth factor (VEGF), phosphoinositide 3-kinase (PI3K)/serine/threonine-protein kinase (Akt)/mammalian target of rapamycin (mTOR) pathways, programmed death-ligand 1 (PD-L1) and adoptive T-cell therapy, which are currently under study [34–38]. The clinical trials are ongoing to further investigate the potential of targeted therapies, both as standalone treatment and in combination with other therapies [39, 40].

Perhaps, the exposure to different risk factors results in the development of distinct histological type of NPC. Furthermore, heterogeneity across individual tumor may cause patient with similar TNM profile to respond differently to the current treatment. All of these reflected the importance of individually tailored NPC therapy. Precision medicine is a logical approach when a more personalized treatment is desired. In precision medicine, the tailoring of disease treatment to a specific patient or subset of patients takes into account their genetic and biological make-up, the environment in which they live, and their lifestyle. In this case, genome mapping and in-depth exploration of the molecular aberrations across the individual or subset of NPC patients are required to discover effective and reliable biomarker in order to develop suitable targeted therapy or to make informed decision of using different combinations and sequences of currently available therapies for subsets of patients. With precision medicine, better therapeutic outcome, quality of life, and more cost effective treatment can be attended. To achieve this goal, having efficient and reliable biomarkers for precise screening, and treatment targets is of paramount importance.

Herein, we present the current status of NPC screening, diagnosis, treatment, and prevention and their respective limitation. In supporting the successful implementation of precision medicine, potential improvements in those aspects are also described. In addition, recent findings on the key molecular aberration involved in pathogenesis of NPC as potential target for precision medicine exploitation are discussed, and the challenge and future outlook of NPC management are also highlighted.

## Existing limitation in NPC: screening, diagnosis, treatment and prevention

### Existing NPC screening, diagnosis and their limitation

#### Clinical presentation

NPC is often diagnosed when patients encountered significant signs and symptoms such as nasal obstruction, epitaxis, conductive hearing loss, cranial nerve neuropathies or lump in the neck. The presence of NPC is usually determined by endoscopic and biopsy-guided examination. In United Kingdom multidisciplinary guidelines, these assessments are mandatory with targeted biopsies of the fossa of Rosenmüller after staging scans to avoid false artefacts. Prior imaging assessment before diagnosis are recommended for cancer staging. Multislice computed tomography (CT) scan of the head, neck and chest for all patients and magnetic resonance imaging (MRI) scans of the skull base in locally advanced tumors. Positron emission tomography–computed tomography (PET–CT) is recommended for occult primary tumor in the nasopharynx [41]. According to ESMO-EURACAN clinical practice guidelines, primary nasopharyngeal tumor is defined by endoscopy biopsy and if no visible positive tumor is discovered, imaging assessment such as MRI and PET is suggested to confirm the diagnosis. Nevertheless, first sign of disease is often appeared in neck nodes, where neck biopsy or neck node dissection is not recommended due to impact of late treatment sequelae [42]. NPC tumor cells arising from the mucosal epithelium of the nasopharynx which is located deep inside the head has created the difficulty for diagnostic evaluation. Concerning that malignancy cannot be determined on cross-sectional imaging, ultrasound guided fine needle aspiration cytology (FNAC) of suspected cervical lymph node metastases is recommended [41]. Despite this, the tumor lining in submucosa may lead to false negative in endoscopy examination. In a retrospective study by Wang et al., nearly one third (33%) of 101 NPC patients had been misdiagnosed as normal in the initial nasopharyngoscopy and imaging assessment, 29% were associated with endophytic on radiographic imaging, which were very likely judged as submucosa on endoscopic examination [43]. In non-endemic zone, MRI is not the primary setting of imaging assessment. The radiographic imaging in this study was in mixture type, where MRI only consisted of 34%, 58% were CT and 8% were PET–CT. The authors highlighted that the high false negative rates may be due to the subjective judgement of radiologists when they receive imaging order from clinicians without further communication, resulting in oversight of NPC signs in the imaging examination [43]. Due to the asymmetric nature of NPC symptoms, non-specific signs and painless lump often lead to advanced stage detection. MRI has been widely used for disease diagnosis for its non-invasive benefit and its ability to produce high resolution anatomical images. King and the team conducted MRI examination on 275 patients with positive plasma EBV DNA, followed by blinded endoscopy examination, whereby the results revealed that MRI had more than 90% of sensitivity and specificity in identifying primary nasopharynx tumors. It had also detected 10% of hidden tumor from endoscopic view [44]. Another study with high sensitivity (98.1%) and specificity (91.7%) for lesion detection has suggested that MRI can be the primary diagnostic tool for NPC detection [45].

In NPC management, artificial intelligence (AI) application play a role in varies area: early detection, diagnosis and treatment, as several positive outcomes have been reported for the smart recognition in the imaging examination (CT, MRI and PET–CT), AI able to reduce biases of radiologists and workloads of physicians in the faster manner, enhance its accuracy for diagnosis and staging analysis. Involvement of AI application in medical sciences, is the trend of every discipline including healthcare system. Basu et al. listed several AI systems in the world, which focus on patients-, clinicians-, and administrative-orientated AI. With large amount of dataset processing for the disease simulation, AI comprises of machine learning (ML) and deep learning (DL) algorithms. However, AI analysed results by trained examples, not all the cases providing same parameters, wrong algorithms may occurred due to the complexity pathogenesis of the disease as well as biased of individuals. As such, it requires enormous dataset, not only general but also biased models to be trained. This raise to the concerns of privacy and security issues. Nevertheless, the complexity of the diseases still required physician to deliver the decision and diagnosis, reinforces with comprehensive diagnostic tool to provide precision detections [46, 47].

Positive EBV expression is highly associated with non-keratinizing carcinoma. The EBV-ISH expression in histological specimen has long been used as a diagnostic marker of NPC. Recently, the use of PCR technique to detect the amount of plasma EBV DNA in patients has been broadly studied for predicting the tumorigenesis of NPC due to its high sensitivity and specificity. In 8th edition of the American Joint Committee on Cancer (AJCC), it was recommended to incorporate pretreatment plasma EBV DNA into the staging system for prognostic surveillance. Rueda Domínguez and team have suggested to include pretreatment plasma Epstein–Barr virus (EBV) DNA levels in the diagnostic and staging evaluation in the clinical practise guideline (SEOM-TTCC clinical guideline). Moreover, the ESMO-EURACAN clinical practice guides recommended the use of plasma EBV DNA, coupled with endoscopic examination and MRI examination, to detect early, asymptomatic NPC [III, A] [42, 48].

Early detection improves survival outcomes, it is therefore crucial to explore a promising biomarker as a diagnosis tool. Apart from histological specimen, a wide range of molecular level detection methods has been studied to overcome the limitations of clinical presentation.

#### Molecular level detection

In non-endemic countries such as United States and some areas with low incidence and sporadic cases, instead of routine screening for NPC, individuals are suggested to approach dentist for regular check-ups [43, 49]. Interestingly, various subtypes showed differential incidence in endemic and non-endemic area. Keratinizing NPC is more prominent in non-endemic area. On the other hand, in endemic area, a higher incidence of non-keratinizing type was reported, which is more highly associated with EBV infection. In Southern China and other countries, EBV serology test has been carried out in the early diagnosis and screening since the 1970s. Infection of EBV can be identified by viral specific antibodies against EBV-associated antigen: viral capsid antigen (VCA), Early antigen (EA) and nuclear antigen (EBNA). Antibodies VCA-IgM developed when primary infection occurred, then disappeared after 4 to 6 weeks, therefore the presence of VCA-IgM indicates early infection marker. VCA-IgG and EBNA antibodies appearance indicates the recent or past infection and most probably persist lifelong in human body [50]. However, among several EBV antibodies, higher titer of IgA was detected in the majority of NPC patients, hence it has marked its prominence as the biomarker for EBV detection. To date, VCA-IgA, EA-IgA, and EBNA1-IgA, Epstein Barr nuclear antigen 1 (EBNA1)-IgA, either in single or combination form, are widely used as a diagnostic markers for early detection of NPC.

Serological mass screening in Wuzhou, China was conducted in 1978–1980. The screening was to detect VCA-IgA and EA-IgA antibodies from individuals’ sera. Among 12,932 participants, positive rate of 5.3% VCA-IgA was found. For the EA-IgA serology test, none was detected in the VCA-IgA antibody-negative persons, while 1.9% of EA-IgA was detected among the VCA-IgA antibody-positive person. This result implied the specificity but not sensitivity of EA-IgA in the detection of NPC [51]. This study indicated that the combination of EBV-related markers has increased the sensitivity, specificity, and positive predictive value (PPV); some even discovered the malignancy developed after several years of detection (Table 1) [51–54]. Poor specificity of serological test is often associated with high false positive rates (2–18%) [55], especially for serological marker in single form manner. Biomarker with high false positive rate require repeatable tests or additional clinical examinations to validate the diagnosis. As such, combination of serological markers have been performed to study the feasibility. In the study by Fachiroh and team. reporting high risk population in Indonesia and China, the sensitivity and specificity has increased to 80.5% and 90.1%, respectively with the combination of IgA EBNA1 + VCA, when compared to single peptide either EBNA1 or VCA [56].

**Table 1:** Sensitivity, specificity and PPV of the studies

Study	Sample size	Marker	Sensitivity (%)	Specificity (%)	PPV (%)	Positive results (%)
[57]	12,932	VCA-IgA EA-IgA (among positive VCA-IgA)	–	–	–	5.3% 1.9%
[56]	405^a^	EBNA1 VCA EBNA1 + VCA	88.6 79.8 85.4	80.1 70.9 90.1	80.6 67.7 78.7	–
[58]	22,186	Plasma EBV DNA	87.5	98.9	41.2	–
[59]	317^b^	IgA-VCA EBV DNA IgA-VCA + EBV DNA	81 95 99	96 98 96–98	–	–

^a^151 NPC patients + 254 healthy individuals

^b^139 NPC patients + 178 healthy individuals

Scientists put effort on viral load measurement to predict the NPC development in high-risk population. EBV infection is usually asymptomatic and persists lifelong in memory B-cells. During the latent infection, EBV circularize into an episomal form at low viral level with approximately 1 in 10,000 to 100,000 memory B cells. The EBV-infected cell thereafter proliferated and transited into peripheral blood. The infected B-cells enable lytic reactivation and a high viral genome to be generated during this phase. This has enabled detection of cell-free EBV DNA in the plasma and serum in NPC [60, 61]. Compared to serological test, meta-analysis supported that EBV DNA screening has higher sensitivity and specificity in NPC diagnosis, whereby the plasma has higher performance than serum samples [62, 63].

In southern China from 2006 through 2013, one large scale population-based cohort study was conducted for EBV DNA load screening in the prospect of early detection of NPC in high-risk population. Among 22 186 participants, 1070 (4.8%) with VCA-IgA titer ≥ 1:5 were defined as high-risk NPC group and further followed up for NPC occurrence. A non-invasive nasopharyngeal swab was used in 905 high-risk group, and 89% was detected as EBV DNA positive, while eight subjects with EBV DNA load higher than the cut-off value, had EBV-DNA load elevation, and seven of them developed early-stage NPC. More than 95% subjects that had lower cut-off value which indicated low NPC occurrence, were excluded from follow up. Their finding suggested that EBV DNA was not only able to detect the early stage of NPC but also filter out the high risk individuals in priority and ease the burden of public health. The sensitivity, specificity, PPV, negative prediction value are 87.5%, 98.9%, 41.2%, and 99.9%, respectively after the optimization of EBV load cut of value (means + 2SD) (4.7 × 10^5^ copies/swab) [58].

A plasma DNA screening study in Hong Kong with large cohort of 20 174 participants was conducted between July 2013 and February 2016. Only Chinese males aged 40 to 62 years were recruited. All participants underwent circulating DNA screening and participants with persistently positive result were assessed with endoscopy and MRI investigation. From the screening, 309 of participants was detected positive, while 34 of 308 participants were confirmed to develop NPC within 1 year. Proportion of stage I/II disease was 71%. One participant who had positive result had declined to follow up thus excluded from the 309-positive analysis, but the participant developed advanced NPC within 32 months. Three participants who were screened positive had negative outcome in the initial nasal endoscopic evaluation and were detected with tumor in MRI examination. The results have highlighted that EBV DNA plasma load is a promising screening tool in detecting early asymptomatic NPC, with PPV, sensitivity, and specificity of 11%, 97.1% and 98.6%, respectively, and patients with NPC detected in this manner had significantly longer progression-free survival (PFS) [1]. The extensive study for plasma DNA profile of the cohort was reviewed by the same team. They perform quantitative analysis and size profiling of the circulating tumor DNA (ctDNA) via real-time polymerase chain reaction (PCR) and revealed that the amount of plasma EBV DNA is higher and the fragment lengths of plasma viral molecules is longer in NPC patients when compared with non-NPC patients. This has enhanced the PPV to facilitate the single time-point testing for NPC screening [64]. Apart from combination of dual serological biomarkers, Leung and their team combined both most sensitive markers with two different mechanisms: IgA-VCA and plasma EBV DNA. The study has demonstrated a high accuracy result with 99% sensitivity and 96–98% specificity [59]. Compared to other markers, EBV DNA has become a promising marker in clinical setting and was recommended in clinical practice guides. It aimed to NPC’s early diagnosis and prognosis surveillance. Several studies have proven combination assay of markers able to increase their sensitivity and specificity. However, false results may occur due to biases pathogenic of individuals, clinical setting and disease management of authorities. Since early detection would improve treatment outcome, establish more effectiveness molecular biomarkers and advance technologies may resolves the problems.

Of note, increasing implementation of New Genome Sequencing (NGS) has enhanced genomic, transcriptomic, proteomic, and metabolomic research. Several types of NPC-associated genomic biomarkers have been identified for early diagnosis and prognosis determination [25, 65]. EBV tumorigenesis, genomic alteration, or somatic variants including single-nucleotide polymorphisms (SNPs) and copy number variations (CNVs) has played a role in the NPC development. Crucial factors of epigenetic alterations and DNA methylations are also documented for this complicated malignancy.

Polymorphism in HLA genes is well associated with NPC susceptibility; HLA-A, -B, -C, -DRB1, and -DQB1 loci were determined as common and well-documented alleles in Chinese population. Genome-wide Association Study (GWAS) mapping within the MHC region of chromosome 6p21 from Taiwan study had identified the association of HLA-A*0207 homozygous allele and the rs29232 (GABBR1) allele in high risk NPC. Strong association of HLA-A allele HLA-A*11:01 and NPC had been identified among Malaysian Chinese population. Interestingly, HLA alleles and haplotypes had varied association in different geographical areas. HLA-A2 and HLA-B46 have higher frequency in high risk incidence Chinese area, while HLA-A10 and HLA-B18 were reported to have higher frequency in Tunisian and Moroccans. The authors suggested the conductance of an extensive study for the relationship of EBV peptides and environmental factors to genetic predisposition for NPC development [66–68].

Aberrant DNA methylation in NPC is commonly associated with epigenetic alteration. Sun et al. detected high frequency of CDH13 methylation which was significantly showed in NPC biopsies with NP swab specificity of 81% and 0% of false positive [15]. RERG, ZNF671, ITGA4, and SHISA3 plasma circulating cell-free DNA (ccfDNA) samples showed significant higher methylation rate, where combination of RERG and ZNF671 gave a 88.5% accuracy by Restriction Digestion and Real-Time PCR (qAMP) [23]. Another study of patients’ noninvasive tissue and brushing sample from Ye et al., revealed that methylation of the RAS association domain family protein 1A (RASSF1A) promoter methylation was found significantly higher in later stage of NPC (T3–T4), suggested RASSF1A promoter methylation which could be a promising diagnostic biomarker [69].

Bruce et al. demonstrated a whole genome profiling, has revealed 90% of NPC undergone constitutive NF-κB activation, either of somatic alteration or interaction of viral LMP1 oncogene, implicated that NF-κB signalling pathway is the hallmark of NPC. From the mapping of this study shown there were 32–34% of NPC exhibited homozygous MAT2A deletion, which observed is pharmacologically vulnerability to MAT2A inhibitors. Thus, identification of MAT2A inhibitors in MTAP-deleted NPC perhaps can be used as future precision therapy trial [70].

For future studies of NPC, comprehensive omics technology can be used to delineate EBV tumogenesis and NPC development, which will be beneficial to the algorithm for precision diagnostic and therapeutic, as well as to facilitate clinical demonstration.

### Existing NPC treatment and its limitation

#### Radiotherapy

A recommended current treatment guideline for NPC is shown in Table 2. Due to the high radiosensitivity of NPC (especially those with EBV infection), its anatomical location, and structure, radiotherapy is the main standard treatment for NPC, but its success rate is depending on the cancer stage. Radiotherapy works effectively in stage I and II NPC patients, with good prognosis achieving 5-year OS of 90% and 84%, respectively [71]. However, for stage III and IV NPC patients, 5-year OS was reported at 55% and 30%, respectively [72]. Therefore, radiotherapy is only effective for early stage and non-metastatic NPC [73, 74]. Moreover, because the nasopharynx is located in close proximity with many critical organs and structures e.g. nerves, optic, parotid glands, brainstem, and temporomandibular joints structures, radiation in this area is associated with toxicities that significantly affects the patient’s quality of life [75–77]. Hence, a more precise approach is necessary to deliver the beam without affecting normal tissues.

**Table 2:** Current treatment guideline for NPC. Source: Referred to NPC clinical practice guideline [42]

Stage	Treatment plan
I	IMRT
II	IMRT, IMRT + CT
III	IMRT + CT, ICT + IMRT/CT, IMRT/CT + AC
IV	ICT + IMRT/CT, IMRT/CT + AC, IMRT/CT
Recurrence	IMRT + surgery, IMRT + CT
Metastatic (newly diagnosed)	CT
Metastatic (not newly diagnosed)	CT + RT

*AC* adjuvant chemotherapy, *CT* chemotherapy, *ICT* induction chemotherapy, *IMRT* intensity-modulated radiotherapy, *RT* radiotherapy

In recent decades, a more precise radiotherapy technique, intensity-modulated radiotherapy (IMRT), is widely used for NPC treatment. It allows the delivery of high radiation dose to complex tumor volume which is located close to the critical structure and low dose to the nearby healthy tissues. With the very satisfactory clinical outcome and minimized treatment-induced toxicities, IMRT is currently being applied as monotherapy particularly in early stage of NPC [78]. IMRT showed excellence results in both locoregional control (90–100%) and OS for all stages of non-metastatic NPC [77, 79]. Although IMRT improved the toxicities and quality of life, there were 10–15% of these patients whose tumors developed radioresistance, distant metastases, and disease recurrence. In addition, reirradiation using IMRT with or without concurrent chemotherapy was recommended as the most effective treatment for recurrent NPC, which reported a 5-year OS of 41% and a long term survival can be achieved in local recurrence [80]. Unfortunately, reirradiation induces severe toxicities especially endangering to adjacent mucosal and neural structure, which lead to 50% of treatment-related mortality [80]. Of particular note in numerous studies, salvage IMRT could induce critical adverse toxicities and fatalities. Therefore, this has limited its application.

In-depth studies of radioresistance mechanisms in NPC are important for developing therapeutics that enhance radiosensitivity, which can facilitate tumor regression, minimize the radiation doses, achieve tumor control and ultimately improve patient survival rates and quality of life. Several mechanisms of radioresistnace in NPC have been reported, including abnormal of genes expression [RNA-binding motif protein 3 (RBM3)], gene mutation and aberrant activation of signalling pathways (NF-κB signalling) [81, 82]. For instance, RBM3 promote radioresistance by supressing the apoptotic response via PI3K/Akt/Bcl-2 signalling [81]. Aberrant activation of NF-κB signalling has also reported to contribute to radioresistance in NPC [82]. RBM3 and abnormal NF-κB signalling could serve as potential biomarker to predict the radiosensitivity, and as target for targeted therapy or immunotherapy to develop its corresponding inhibitor for radiosensitization. Moreover, a study has reported that the combination of targeted therapy, nimotuzumab with IMRT significantly improved the PFS (83.29%) and OS (97.6%) and was associated with lower acute toxicity [83]. However, more randomized clinical trials are needed to verify the clinical efficacy of this treatment. With this encouraging study, future endeavours could focus on exploring combination of targeted therapy or immunotherapy with IMRT to enhance radiosensitivity and reduce adverse toxicities, thus improving the IMRT treatment outcomes. Furthermore, identify molecular markers or genetic signatures associated with radioresistance in NPC patients, allowing for more personalized treatment strategies. Also, exploring advanced imaging methods like functional MRI or PET scans, to better delineate tumor boundaries and critical structures facilitate in precision of radiotherapy delivery.

#### Chemotherapy

Favorably, NPC is also highly sensitive to chemotherapy. Studies on cisplatin-based chemotherapy were reported with response rate up to 80% [84, 85]. For intermediate to advanced stages of NPC, radiotherapy alone is not sufficient. Concurrent chemoradiotherapy (CCRT) is recommended by National Comprehensive Cancer Network (NCCN) as a primary treatment for locoregionally advanced NPC. Cisplatin is the only chemo-drug recommended by NCCN clinical guidelines to be used in CCRT. Concurrent treatment with cisplatin-based chemotherapy and IMRT were reported with 5-year OS of up to 90% [86, 87]. Currently, more intensive systemic treatments such as induction therapy preceding chemoradiotherapy and adjuvant therapy after chemoradiotherapy are required for stage II–IVA NPC. A clinical trial revealed that as compared to chemoradiotherapy alone, improved OS and PFS were achieved when docetaxel, cisplatin, and 5-fluorouracil (5-FU) were given prior to chemoradiotherapy [88]. Contrastingly, there was another study reporting the lack of significant difference of OS in using induction therapy, thus long-term follow-up study is needed to confirm the clinical efficacy [89]. Meanwhile, the efficacy of adjuvant chemotherapy is also arguable [90, 91]. Moreover, complete administration of adjuvant chemotherapy is difficult to execute as approximately 40% of patients could not complete the treatment cycles and reduction of the planned dose was required [91]. A phase III trial showed no significant difference in survival benefit with the addition of cisplatin and 5-FU as the adjuvant therapy after chemoradiotherapy in advanced stage of NPC [90]. In contrast, a cohort study revealed that an improved OS was achieved when combination of cisplatin and 5-FU, cisplatin and docetaxel, or cisplatin, 5-FU, and docetaxel were given after chemotherapy in locoregionally advanced NPC [92]. In addition, study also demonstrated that up to 15% of these patients have developed distant metastasis and recurrence [75]. Therefore, the consensus on whether induction or adjuvant therapy provides better survival with no increased toxicities has yet to be achieved. For patient selection criteria, individual patient characteristic or genetic and tumor profile should take into account when selecting the patient for treatment response evaluation.

Currently, platinum doublet of gemcitabine and cisplatin chemotherapy has taken over the old standard 5-FU plus cisplatin treatment as the first line therapy for recurrent and metastatic NPC [93]. Compared to 5-fluorouracil plus cisplatin treatment, a phase III clinical trial demonstrated that overall response rate (ORR) and PFS were significantly improved when treated with gemcitabine and cisplatin [93]. There are numerous studies reported high response rate of using poly-drugs chemotherapy for recurrent NPC [26, 94]. However, it is associated with high toxicities such as leucopenia, neutropenia, mucosities and thrombocytopenia [94]. For early metastatic NPC, combining locoregional radiotherapy with chemotherapy could improve the OS. Nevertheless, there is no standard second-line treatment for metastatic NPC.

Despite that, recent study has demonstrated that drug responses are well correlated with corresponding individual genomic and tumor profile [95]. Therefore, patient with same stage of cancer may respond differently to the chemotherapy. To address this limitation, future researches should focus on studying the heterogeneity of NPC tumor, identifying the prognostic biomarker for specific chemotherapy regimens, as well as developing personalized treatment approaches.

#### Surgery

Surgery is generally used to remove the lymph node metastases in the neck or tumor from the nasopharynx, which requires an incision on the roof of patient’s mouth in order to reach the nasopharynx to remove the tumor. Due to the anatomic localization adjacent to key neurovascular structures, surgery is not the upfront treatment in NPC, however, it plays a significant role in treating post radiation residual, NPC recurrence and neck nodes with or without combination of radiotherapy or CCRT [75]. Salvage radical neck dissection was suggested as one of the treatment choice for persistent or recuurence NPC following chemoradiotherapy [41]. With the advancement of technologies, minimally invasive approach has emerged such as endoscopic nasopharyngectomy, which is commonly used in Asia countries. Endoscopic nasopharyngectomy is a less destructive surgical for resection of early-stage recurrent NPC and locoregional residual, with disease free survival (DFS) and OS of 90% and 100% respectively [96]. Another study also demonstrated endoscopic nasopharyngectomy in recurrent NPC with 56.1% of them were T1 recurrence, the recurrence-free survival and OS were achieved up to 85.8% and 82.9%, respectively [96]. Compared to reirradiation with IMRT in recurrent NPC, endoscopic nasopharyngectomy has resulted in better survival rate and reduced late treatment complication. Therefore, salvage nasopharyngectomy for recurrent NPC is highly promising. Moreover, the addition of adjuvant reirradiation prior to surgery for recurrent NPC has shown better OS (63% vs. 39%) compared to surgery alone [97].

More recent studies have also reported that¸ compared to IMRT, stage I patients treated with endoscopic nasopharyngectomy have shown improved 5-year OS (100% vs. 99.1%), local relapse-free survival (100% vs. 97.7%), regional relapse-free survival (100% vs. 99%) and distant metastasis-free survival (DMFS) (100% vs. 97.4%) [98]. This has supported the fact that endoscopic nasopharyngectomy for early stage of NPC can improve clinical outcome, provide better quality of life, and depress medical cost when compared to the IMRT procedures [96, 99]. Therefore, endoscopic nasopharyngectomy is suggested as an alternative treatment for stage I NPC patients. Nevertheless, the success of surgical excision is varying with the tumor size and extent of the recurrent [41].

To overcome this constraint, forthcoming research should emphasize the enhancement of surgical techniques and patient selection. This is especially crucial for individuals with larger tumors or extensive recurrences, aiming to enhance treatment effectiveness while minimizing complications. Further verification studies are also required to compare surgery to re-irradition with or without combination with systemic therapy in recurrent or staged I NPC patients, and to cover variable factors such as tumor size and patient status could provide insights in discover optimal treatment method. Investigating combination therapies that integrate surgery with other modalities such as immunotherapy, presents a promising path for future research aimed at improving treatment results. Furthermore, exploring the long-term survival benefits and advancement in quality of life among patient treated with endoscopic nasopharyngectomy compared to traditional treatment methods, may offer valuable insights to shape forthcoming treatment guidelines.

Summaries from above NPC treatments: although current treatments in early stage and locoregional advanced NPC generally showed good prognosis and improved OS, but the outcome for those with recurrent and metastatic NPC remains poor. There were about 20–30% of patients with locoregionally advanced NPC died due to development of distant metastasis. Furthermore, the emergence of radioresistance, locoregional recurrence, and distant metastasis often happened after radiotherapy or chemotherapy, especially those with advanced stage. Since the benefits of concurrent chemotherapy in NPC have reached the plateau, thus a new precise treatment is urgently needed. Recently, immunotherapy which comprises immune checkpoint inhibitors combined with precision radiotherapy with or without chemotherapy has also been suggested as promising alternative to the current treatment in NPC.

### Existing NPC prevention and its limitation

NPC is highly associated with EBV infection with the prevalence of 95%, mainly in undifferentiated NPC [100–102]. Since EBV is a highly relevant risk factor of NPC, scientists put effort on the vaccine to trigger human immune response against EBV infection since decades ago. However, there is no licensed prophylactic EBV vaccine so far.

EBV establishes latent infection in B cells and lytic infection in epithelial cells. While EBV primarily targets the human B-cells, a cell-to-cell infection is the dominant transmission of EBV virus. Five viral membrane envelope glycoproteins have involved in this mechanism: gp350, gB, gH, gL, and gp42; gp350 is the dominant glycoprotein expressed on EBV extracellular envelope [103]. EBV virions infects B-cells through gp350 by attaching themselves to CD21. By blocking the infection of B-cell, vaccination of gp350 is targeted as a potential candidate to prevent EBV-associated diseases. A gp350 vaccination phase II clinical trial which was performed on 181 EBV-seronegative subjects aged 16–25 years was the largest gp350 vaccination cohort by far. Three doses of recombinant gp350 vaccine with monophosphoryl lipid A (MPL) adjuvant system [aluminum hydroxide and 3-*O*-desacyl-4′-monophosphoryl lipid A (AS04)] was given in the double-blind, placebo-controlled randomized trial. This study showed that the vaccine was immunogenic and it is capable of preventing NPC induced by EBV infection with 78% efficacy and successful induction of neutralizing antibodies for a period up to 18 months. However, it failed in preventing the asymptomatic EBV infection (Table 3) [104].

**Table 3:** Summaries of EBV vaccine trials

Study	Vaccine/(adjuvant)	Target subjects	Clinical trial	Results
[104]	Monomeric gp350/(MPL)	181 EBV-seronegative young adults	Phase II	Efficacy: 78%; neutralizing antibodies was induced up to 18 months, but no prevention against asymptomatic EBV infection
[105]	Monomeric gp350/(alhydrogel)	16 EBV-negative children with CKD, candidates of renal transplantation	Phase I	13 recipients had IgG response; only 4 recipients induced neutralizing antibodies but immune response declined rapidly. Poor immunogenicity against PTLD protection
[106]	Trimeric gH/gL and gB/(alum + CpG-ODN)	Rabbit	–	Potent EBV-neutralizing titers induced, neutralizing titre were > 100-fold than monomeric gp350 > 20-fold than monomeric gh/gL > 18-fold than trimeric gB, and > fourfold than tetrameric gp350
[107]	Chimeric VLP: EBV gp350/220	BALB/c mice	–	Long-term neutralizing antibodies was induced
[108]	Packaging cell line VLP: modified EBV genome with deletion of TR	Epithelial cell line	–	Able to target B-cell in vitro, unwanted recombinant DNA was performed
[109]	Packaging cell line VLP: modified EBV genome with inactivation of six viral genes	293-VII + producer cell line, and BALB/c mice	–	High immunogenicity, induced potent neutralizing polyvalent antibodies and T-cells responses in vitro and in vivo models
[110]	Latent protein as vaccine candidates: CD8^+^ T-cell peptide Epitope-based/(water-in-oil emulsion)	14 HLA B*0801 positive, EBV-seronegative adults	Phase I	1/2 placebo recipients acquired developed IM 4/4 peptide recipients acquired asymptomatic EBV infection

Another gp350 vaccination trial was performed to prevent immunosuppressed patients from acquiring EBV infection. To study the ability of vaccine to lower the risk of posttransplant lymphoproliferative disease (PTLD), 16 EBV-negative children with chronic kidney (CDK) disease were recruited in a phase I trial for gp350/alum vaccination. The vaccine was immunogenic, all of the 13 recipients had IgG response although 12 recipients received three doses vaccination and one received two doses vaccination. The neutralizing antibodies were only detected in four recipients. However, immune response declined rapidly before transplantation surgery which resulted in poor immunogenicity to protect patients from developing PTLD [105].

Beside the monomeric gp350 vaccine, a novel trimeric EBV gH/gL, and gB vaccine was studied in rabbit model. gH/gL and gB are EBV envelope glycoproteins contributing to fusion machinery during the viral entry to B-cells and epithelial cells [111, 112]. Multimeric vaccine was expected to elicit potent neutralizing antibodies. The result was encouraging with neutralizing titer detected > 100-fold, 20-fold, 18-fold, and fourfold higher than monomeric gp350 vaccine, if compared to recombinant trimeric and monomeric gH/gL, trimeric gB, and tetrameric gp350 respectively. This had indicated that multimeric vaccine might be a better vaccine type for preventing EBV infection in B-cells and epithelial cells [106]. However, it still needs clinical demonstration to validate the vaccine efficacy and safety for human use.

One of the EBV vaccine strategy is focused on EBV Virus-like particles (VLPs). VLPs comprises viral capsid polymer, envelope, and tegument proteins, assembling the characteristic of native EBV but usually lack of viral DNA and thus, it is not pathogenic to host. One study has designed a chimeric VLP, the fusion of EBV gp350/220 ectodomain to Newcastle disease virus (NDV) F protein. The chimeric VLP was immunized in BALB/c mice without any adjuvant and long-term neutralizing antibodies was induced. However, the titer was lower if compared to the control UV-EBV; the authors addressed that it may due to misfolding of protein domain [107]. Delecluse et al. constructed the first generation of virus-like packaging cell line VLP. This system contains modified EBV genome with deletion of terminal repeat (TR), and is able to target B-cell and epithelial cells to induce immune response, but unwanted recombination of helper virus genome and gene vector DNA was found [108]. To further improve the safety issue, Ruiss et. al introduced the second generation packaging cell line VLP, with inactivation of six viral genes [EBNA2, latent membrane protein (LMP) 1, EBNA3A,-B, and -C, BZLF1]. The packaging system elicited potent neutralizing polyvalent antibodies and strong CD8^+^ and CD4^+^ T cell response in immunized BALB/c mice [109]. This VLP system was able to deliver large amount of genome and was highly immugenic, hence may be the potential alternative to human vaccination.

Another approach was the latent protein vaccine trial. A single blind, randomized, placebo-controlled, single-center phase I clinical trial for 14 human leukocyte antigen (HLA)-B*0801 positive, EBV-seronegative adults was conducted, where ten were peptides vaccine recipients and four were placebo recipients. CD8^+^ T-cell peptide epitope with HLA-B*0801 restricted peptide epitope FLRGR AYGL and tetanus toxoid vaccine, adjuvant with water-in-oil emulsion was conducted. After vaccination, EBV-specific T-cell responses were detected in eight of nine recipients. None of the recipients developed Infectious Mononucleosis (IM). After 2–12 years follow-up, one of two placebo developed IM, while four of ten recipients who completed two doses vaccinations had asymptomatic EBV infection. The outcome was similar to monomeric gp350 trial, which denotes that there is still a challenge for developing vaccine to prevent EBV infection [110].

mRNA vaccine had been studied since 1960’s. mRNA vaccines were widely introduced for its high efficacy, rapid development, and cost effectiveness during the COVID-19 pandemic. Therefore, it has served as promising alternative to conventional vaccine approaches. By using lipid nanoparticles as a media, synthetic mRNA that corresponds to a viral protein has been introduced to human body. After cells translation machinery, viral protein was produced and subsequently triggering our immune response to create antibodies to fight against the virus infection [113]. This new type of vaccine has shed light to prophylactic EBV vaccines, with the aims to reduce the spread of contagious EBV infection and its global prevalence. It showed that mRNA vaccine can be a potential tool for preventing EBV-associated NPC. Of note, one of the pioneering mRNA vaccine companies, Moderna, is recruiting 18–30-year-old healthy adults to a phase I (NCT05164094) clinical trial for mRNA-1189 EBV vaccination with four glycoproteins gH, gL, gp4, and gp220 as candidates. The other mRNA-1195 (NCT05831111) also being developed by the company to fight against long-term EBV sequelae for preventing EBV reactivation in human body [114]. Nevertheless, the safety and efficacy of the mRNA vaccine need to be measured for prophylactic and preventive use.

## Molecular aberrations exploitation in NPC for precision medicine

NPC carcinogenesis comprises a complex process of genetic and epigenetic alterations which are driven by diverse oncogenic molecular events. For example, oncogenic DNA methylation, histone modification, abnormal activation or miRNAs/lncRNAs silencing are the epigenetic mechanisms that are involved in NPC pathogenesis [115]. Besides that, hyperactivation of cellular signalling pathways such as prosurvival pathways, PI3K/Akt, NF-κB, mitogen-activated protein kinase (MAPK)/extracellular signal-regulated kinase (ERK), signal transducer and activator of transcription (STAT) 3, Wingless-related integration site (Wnt)/β-catenin] and abnormal negative regulation of proapoptopsis pathways (p53, endoplasmic reticulum stress), and tumor necrosis factor (TNF) signalling pathway are associated with NPC pathogenesis [116] (Table 4). EBV infection is usually the main driver of these molecular alteration in non-keratinizing NPC. Different molecular aberrations across the individual has led to different response towards similar treatment. Therefore, specifically targeting certain aberrant molecules that participate in NPC tumorigenesis could potentially improve the treatment response. In this section, we will describe commonly recurring molecular aberrations in NPC that could potentially be targeted and help segregating individual patient for a more precise disease management.

**Table 4:** Signalling molecules targeted for therapeutic exploitation

Signalling pathway	Target	Oncogenic role	Potential therapeutic approach	Source
PI3K/Akt/mTOR	PI3K	NPC progression, CSC properties metastasis, radioresistance, cisplatin resistance and cytoskeleton dynamic	Inhibit PI3K by 2–4-morpholinyl-8-phenlchromone, omipalisib, gedatolisib, euscaphic acid or FOXO1	[36, 38, 117, 118]
	FGF2	Proliferation, migration, and invasion	Inhibited by miR-16	[119]
	Flot-2	Metastasis and cell proliferation	Silencing Flot-2	[120]
	DNMT1	Methylated miR152, downregulation of PTEN, activating Akt, inhibiting the miR-142-3p/Zinc-finger E-box binding homeobox 2 (ZEB2) axis (Invasion, migration, EMT and metastasis)	Downregulation	[121]
	SREBP1	Lipid synthesis promote cell proliferation and invasion	Downregulation	[122]
	EpCAM	CSC, metastasis	Downregulate by MK2206 and rapamycin	[123]
	HIF-1α	VM formation	Downregulation	[124]
	COL1A1	Radioresistance	Downregulating by MiR-29a	[125]
	RBM3	Radioresistance	Downregulation RBM3	[81]
	p85	Radioresistance and tumourigenesis	Interacting with leucine zipper tumor suppressor 2 (LZTS2)	[126]
	MK2	EMT, cisplatin resistance	downregulating by miR-296-3p	[127]
	VPS33B	Proliferation and chemoresistance	Upregulated by disrupting interaction with NESG1	[128]
	Cyclin D1(CCND1)	EMT	Downregulating miR-374a	[57]
	MDK	Angiogenesis	Downregulating of by MiR-9	[129]
TP53	MDM2	Chemoresistance	Competitive binding with Nutlin-3 and Idasanutlin	[130, 131]
	COX-2	Chemoresistance	Downregulation	[132]
	FOXO1	Chemoresistance, CSC and EMT	Upregulated by CB	[133]
	miR-125a and b	Anti-apoptosis	Downregulation	[134]
NF-κB	miR-203	EMT and metastasis	Upregulated by aspirin	[135]
	Pim 1	Cell proliferation	Suppressed by quercetagetin	[136]
	IKK	Anti-apoptosis	Downregulate by flavonoid glycoside vitexin	[137]
	VEGF	Angiogenesis	Suppressed by andrographolide	[138]
	ICAM	Apoptosis, cell proliferation		
	MMP-9	Invasion and metastasis		
	IκBα	Apoptosis and cell proliferation	Suppressed by simvastatin	[139]
	BST2	Chemoresistance	Downregulation	[140]
	SIRT6	Metastasis and anti-apoptosis	Upregulation	[141]
	NEAT1	Cell proliferation and anti-apoptosis	Knockout	[142]
	DLC-1	EMT	Upregulation	[143]
NF-κB	RERG	Angiogenesis cytokines, colony formation, invasion and migration	Demethylation	[144]
	NF-κB p65	Cancer progression	Inhibited by EGCG	[145]
MAPK/ERK/JNK	Amyloid β precursor	Migration, invasion and EMT	Knockdown	[146]
	PAK1	Apoptosis	Inhibited by IVM	[147]
	MAP2K6	ROS and apoptosis	Inhibited by HI-TOPK-032	[148]
	MNK1	Apoptosis	Inhibited by compound 12dj	[149]
	MET	Cell proliferation and radioresistance	Inhibited by PHA-665752	[150]
	BLU	Cell cycle and apoptosis	Upregulation	[151]
	miR-124	TGF-β-induced migration, invasion and cell proliferation	Upregulation	[152]
	miR-483-5p	Colony formation, radioresistance and DNA damage	Downregulation	[153]
	CTAR1	Malignant transformation and cell survival	Inhibition	[154]
	PIN 1	Cell proliferation	Inhibited by juglone	[155]
JAK/STAT	STAT	Angiogenesis, migration, EMT, anti-apoptosis, cell proliferation and metastasis	Directly inhibited by ovatodiolide	[156]
	RKIP	Migration, invasion, metastasis and EMT	Upregulation	[157]
	miR-29a	Cell proliferation, anti-apoptosis and drug resistance (taxol)	Upregulation	[158]
	miR124-3p	Cell proliferation and anti-apoptosis	Upregulated by sulforaphane or knockout of UCA1 gene	[159, 160]
Wnt/β-catenin	CBP	CSC	Inhibited by foscenvivint	[161]
	β-catenin	CSC and radioresistance	Inhibited by binding of Chibby (Cby) to C-terminal of β-catenin • Inhibited it nuclear translocation by 14-3-3 adaptor proteins • Upregulation of miR-34c	[162, 163]
	Capn4	Proliferation and invasion	Inhibited by upregulation of miR-124	[164]
	LHX2	Radioresistance	Inhibited by upregulation of miR-506	[165]
	FOXO3a	Radioresistance	Upregulation	[166]
	Lgr5	Chemoresistance and EMT	Downregulation	[167]

*Akt* serine/threonine kinase 2, *BLU* zinc finger, MYNDtype containing 10, *BST2* bone marrow stromal cell antigen 2, *Capn4* calpain small subunit 1, *CBP* CREB binding protein, *COL1A* collagen type I alpha 1 chain, *COX-2* cyclooxygenase-2, *CSC* cancer stem cell, *CTAR1* carboxy-terminal activating region 1, *DLC-1* deleted in liver cancer-1, *D1(CCND1)* cyclin D1, *EGCG* epigallocatechin-3-gallate, *EMT* mesenchymal transition, *EpCAM* epithelial cell adhesion molecule, *FGF2* fibroblast growth factor 2, *Flot-2* flotillin 2, *FOXO1* forkhead box protein O1, *FOXO3a* forkhead box O3a, *HIF-1α* hypoxia-inducible factor 1-alpha, *HI-TOPK-032* T-LAK-cell-originated protein kinase (TOPK) inhibitor, *ICAM* intercellular adhesion molecule 1, *IKK* IκB kinase, *IκBα* nuclear factor-kappa B inhibitor alpha, *IVM* ivermectin, *Lgr5* G protein-coupled receptor 5, *LHX2* LIM homeobox 2, *MAP2K6* mitogen-activated protein kinase 6, *MDK* midkine, *MDM2* murine double minute 2, *MET* mesenchymal epithelial transition, *MK2* mitogen-activated protein kinase-activated protein kinase 2, *MMP-9* matrix metalloproteinase, *MNK1* mitogen activated protein kinase interacting kinases, *mTOR* mammalian target of rapamycin, *NEAT1* nuclear enriched abundant transcript 1, *NESG1* nasopharyngeal epithelium specific protein 1, *NF-κB p65* nuclear factor kappa-light-chain-enhancer of activated B cells p65, *NPC* nasopharyngeal carcinoma, *PAK1* P21 (RAC1) activated kinase 1, *Pim 1* moloney murine leukemia virus-1, *PIN 1* peptidyl-prolyl *cis*–*trans* isomerase NIMA-interacting 1, *PI3K* phosphoinositide 3-kinase, *RBM3* RNA binding motif protein 3, *RERG* ras-like estrogen-regulated growth inhibitor, *RKIP* Raf kinase inhibitory protein, *SIRT6* Sirtuin 6, *STAT* janus kinase, *UCA1* urothelial cancer associated 1, *VEGF* vascular endothelial growth factor, *VM* vasculogenic mimicry, *VPS33B* vacuolar protein sorting 33B

### EBV driver molecular aberration

EBV is a γ-herpes virus that establishes lifelong asymptomatic infection in human and is responsible for up to 95% of NPC incidence in endemic areas [9]. It has actively contributed in the multiple steps of oncogenesis in many cancer types especially in NPC. Circulating EBV DNA was recognized as the prognostic biomarker complement to the current TNM classification and is used as the precise treatment decision guide for EBV-positive NPC patients [168, 169]. However, the measurement of the plasma EBV DNA is not standardized. EBV exhibits as type II latent infection in NPC which comprises the expression of oncoviral protein, latent membrane proteins (LMP1 and LMP2A or B), EBNA1, Epstein–Barr virus BamHI-A rightward frame 1 (BARF1), small nuclear RNA, EBV-encoded small RNAs (EBERs) and microRNAs, and miR-BamHI-A rightwards transcripts (BARTs) [170]. The heterogeneous interplay between the EBV oncogenic proteins, tumor genetic, and immune microenvironment has led to different subset of NPC patients [171]. These oncogenic products, especially LMP1, aberrantly induced different intracellular signalling pathways such as PI3K/Akt/mTOR, MAPK/EGFR (epidermal growth factor receptor), mitogen-activated protein kinase (MEK)/ERK, c-Jun N-terminal kinases (JNK/c-JUN), VEGF, NF-κB, Wnt/β-catenin, and janus kinase (JAK)/STAT to promote survival, angiogenesis, invasiveness, migration, and metastatic potential. It also assists in immune evasion by modulating the cytokine and chemokine productions [172].

Despite that, the transcriptional level of LMP1 was found in more than 70% of NPC patients [173]. The frequent expression of LMP1 and its role in mediating numerous oncogenic signalling have shown its critical role as a key effector viral oncoprotein in NPC pathogenesis. In recent years, molecular agents targeting EBNA1 and LMP1 have been investigated for NPC treatments. Briefly, LMP1-targeted DNAzymes were used to suppress the expression of LMP1; inhibition of cell proliferation and enhanced radiosensitivity in EBV-positive NPC patients by repressing of LMP1/JNKs/hypoxia-inducible factor 1 (HIF-1)/VEGF-mediated angiogenesis and supressing the LMP1/Akt-induced telomerase activity [174–177]. Moreover, early phase clinical trial have reported that intratumoral administration of EBV-LMP1 targeted DNAzyme with radiotherapy has resulted in significant reduction in tumor regression and associated with low toxicity [178, 179]. Besides that, a theragnostic agent was constructed using the fluorophore L2 and the EBNA1-specific binding peptide P4, which disrupt EBNA1 homodimerization, and up to 93% of EBV-positive xenograft showed growth suppression [180]. With this encouraged studies, further investigations on the combination of therapies targeting EBV-associated proteins with other targeted agents or immune checkpoint inhibitors for NPC treatment are anticipated. In addition, further research could also delve into the intricate interplay between oncogenic proteins of EBV and the immune microenvironment. This exploration might able to uncover novel possibilities for therapeutic intervention.

#### Aberration in lncRNA/miRNA

Distinct long non-coding RNA (lncRNA) and microRNA (miRNA) have been demonstrated to play significant role in NPC pathogenesis [181]. Upregulation of lncRNA/miRNA such as antidifferentiation non-coding RNA (ANCR), metastasis associated lung adenocarcinoma transcript 1 (MALAT1), nuclear enriched abundant transcript 1 (NEAT1), and miR-504 were reported to promote radioresistance via regulating the expression of phosphatase and tensin homolog (PTEN), miR-1/zinc finger protein SNAI2 (slug) axis, remodelling and spacing factor 1 (Rsf-1)/rat sarcoma virus (Ras)-MAPK/let-7a-5p axis, and inhibiting nuclear respiratory factor 1 (NRF1) [182–185]. Besides that, upregulation of lncRNA such as antisense noncoding RNA in the INK4 locus (ANRIL), urothelial cancer associated 1 (UCA1), cancer susceptibility candidate 9 (CASC9), and actin fiber-associated protein 1-antisense RNA1 (AFAP1-AS1) have been revealed to promote cell proliferation, epithelial to mesenchymal transition (EMT), metastasis, and inhibit apoptosis. In addition, upregulation of miR-149 and downregulation of miR-422a were also reported to associate with tumorigenesis [186–190].

Personalized modulation of certain miRNAs expression in individual NPC patient could be an alternative approach for NPC treatment. For example, upregulation of miRNAs including miR-7, miR17-5p, miR-20a-5p, miR-26b, miR29-c, miR-93, miR-101, miR-148b, miR-150, miR-185-3p, miR-205, miR-212, miR-324-3p or miR-432 were associated with tumor suppression by targeting associated genes or signalling as depicted in Table 5. In contrast, downregulation of certain lncRNA or miRNAs could also exert the anti-cancer effect (Table 5). Targeted oncogenic lncRNA or miRNAs can be suppressed by interfering 3′-untranslated regions (3′-UTR) region to inhibit transcription and translation of its oncogenic protein. For instance, downregulation of lncRNA maternally expressed gene 3 (MEG3) and plasmacytoma variant translocation 1 (PVT1) has retarded the NPC progression by inducing cell cycle arrest and apoptosis, and also inhibiting colony formation and cell proliferation [191, 192]. Regulating the lncRNA cancer susceptibility 2 (CASC2)/miR-18a-5p/retinoblastoma binding protein 8 (RBBP8) axis could also suppress the tumor development [193]. Another recent study also revealed that lncRNA, family with sequence similarity 225 member B (FAM225B) was found upregulated in NPC to promote cell proliferation and invasion via overexpression of integrin β3 (ITGB3) and activation of focal adhesion kinase (FAK)/PI3K/Akt pathways [194]. In addition, knockdown of lncRNA forkhead box protein D3-antisense RNA 1 (FOXD3-AS1) has repressed the colony formation, invasion, and migration in NPC by regulating miR-185-3p/FOXD3 axis [195]. Hence, lncRNA NKILA, FAM225A, downregulated RNA in cancer (DRAIC), and FOXD3-AS1 may serve as potential personalized therapeutic targets for NPC.

**Table 5:** LncRNAs and miRNAs targeting for NPC therapeutic

LncRNAs/miRNAs	Potential therapeutic approaches	Anti-tumor effect	Source
miR-7	Upregulated by Curcumin	Triggered cell cycle arrest and apoptosis, retarded cell proliferation, migration and invasion by inhibiting Skp2	[196]
miR17-5p	Downregulation	Suppressed tumour proliferation via regulation of p21 protein	[197, 198]
miR-20a-5p	Downregulation	Enhance radiosensitivity via regulation of *neuronal PAS domain protein 2* (*NPAS2*) gene	[199]
miR-26b	Upregulation	Enhance chemosensitivity (Cisplatin) by inhibiting JAG1 expression	[200]
miR29-c	Upregulated by knockdown of lncRNA X inactive-specific transcript (XIST) or HMG-box transcription factor 1 (HBP1)	Suppressed cell proliferation and enhance radiosensitive via reduce level of cyclin D1 and cyclin D3	[201, 202]
miR-93	Downregulation	Suppressed tumour growth and migration	[203]
miR-101	Upregulation	Suppressed metastasis and angiogenesis by negative regulation of integrin subunit alpha 3 (ITGA3) or Stathmin 1 (STMN1)	[190, 204]
miR-148b	Upregulation	Suppressed invasion, and metastasis by inhibiting *metastasis-related gene 2* (*MTA2*)	[205]
miR-149	Downregulation	Suppressed proliferation, invasion and metastasis via the upregulation of plakophilin3 (PKP3) expression	[187]
miR-150	Upregulation	Suppressed cell proliferation and G1/S transition by targeting CCND1, CCND2, CDK2 and CCNE2	[206]
miR-185-3p	Upregulation	Enhance radiosensitivity through inhibition of mothers against decapentaplegic homolog 7 (SMAD7)	[207]
miR-205	Downregulation	Suppressed tumour growth and migration	[203]
miR-212	Upregulation	Suppressed invasion and migration by targeting SRY-Box Transcription Factor 4 (SOX4)	[208]
miR-324-3p	Upregulation	Suppressed invasion, cell proliferation, apoptosis by negative regulation of *GLI Family Zinc Finger 3* (*GLI3*) gene	[209]
miR-422a	Upregulation	Suppressed EMT and metastasis by targeting FOXQ1	[186]
miR-432	Upregulation	Suppressed invasion and migration by regulation E2F transcription factor 3 (E2F3) expression	[210]
miR-504	Downregulation	Enhance radiosensitivity via upregulation of NRF1	[185]
LncRN UCA1	Downregulation	Suppressed invasion and cell proliferation via miR-145/A disintegrin and metalloprotease 17 (ADAM17) axis	[188]
LncRN CASC9	Downregulation	Suppressed the cancer progression by destabilising the HIF1α	[189]
LncRN AFAP1-AS1	Downregulation	Prevent to be co-expressed with PD-1 to promote immune escape	[211]
LncRN MEG3	Upregulation	Induced cell cycle arrest, inhibited cell proliferation and colony formation	[192]
LncRN PVT1	Downregulation	Enhance radiosensitivity via regulation of DNA damage repair pathway	[191]
LncRN ANCR	Downregulation	Enhance radiosensitivity via regulation of PTEN expression	[183]
LncRN MALAT1	Downregulation	Enhance radiosensitivity via regulation of miR-1/slug axis	[184]
LncRN NEAT1	Downregulation	Suppressed tumourigenesis and enhance chmeosensitivity (cisplatin) via regulation of miR-124 and Rsf-1/Ras-MAPK/let-7a-5p axis	[182]
LncRNA LINC00460	Downregulation	Suppressed EMT via regulation of miR-30a-3p/Ras-related protein 1A (Rap1A) axis	[212]
LncRNA NKILA	Upregulation	Suppressed carcinogenesis through the inhibition of NF-κB signalling	[213]
LncRNA FAM225A	Downregulation	Suppressed cell proliferation and invasion by regulation of ITGB3 and FAK/PI3K/Akt pathways	[194]
LncRNA DRAIC	Downregulation	Suppressed invasion and migration by miR-122/SATB1 axis	[214]
LncRNA TP73-AS1	Downregulation	Suppressed migration and invasion via regulation of miR-495/JAM-A axis	[215]
LncRNA FOXD3-AS1	Downregulation	Suppressed colony formation, invasion and migration via regulation of miR-185-3p/FOXD3 axis	[195]

*AFAP1-AS* actin fiber-associated protein 1-antisense RNA1, *Akt* serine/threonine kinase 2, *ANCR* antidifferentiation non-coding RNA, *CASC9* cancer susceptibility candidate 9, *CCND 1 or 2* cyclin D1 or 2, *CCNE2* cyclin E2, *CDK2* cyclin dependent kinase 2, *DRAIC* downregulated RNA in cancer, *FAK* focal adhesion kinase, *FAM225A* family with sequence similarity 225 member A, *FOXD3* forkhead box protein D3, *FOXD3-AS1* forkhead box protein D3-antisense RNA 1, *FOXQ* forkhead box protein Q1, *ITGB3* integrin β3, *JAG1* jagged 1, *JAM-A* junctional adhesion molecule-A, *LINC00460* long intergenic non-protein coding RNA 460, *LncRNA* long non-coding RNA, *NF-κB* nuclear factor kappa B, *MALAT1* metastasis associated lung adenocarcinoma transcript 1, *MEG3* maternally expressed gene 3, *NKILA* NF-kappabeta-interacting long noncoding RNA, *PD-1* programmed cell death 1, *PI3K* phosphoinositide 3-Kinase, *PTEN* phosphatase and tensin homolog, *PVT1* plasmacytoma variant translocation 1, *p21* cyclin-dependent kinase inhibitory protein-1, *Ras-MAPK* rat sacrcoma-mitogen-activated protein kinase, *Rsf* remodelling and spacing factor 1, *SATB* special AT-rich sequence-binding protein-1, *Skp2* S-phase kinase-associated protein 2, *slug* zinc finger protein SNAI2, *SRY-Box* sex-determining region Y protein-Box, *TP73-AS1* P73 antisense RNA 1T, *UCA1* urothelial cancer associated 1

#### Aberration in signalling pathways

- Targeting PI3K/Akt/mTOR
  PI3K/Akt/mTOR pathway is constitutively activated in EBV positive NPC by EBV oncoproteins (LMP1 and LMP2A) or through the infrequent mutations in its regulators such as phosphatidylinositol-4,5-bisphosphate 3-kinase catalytic subunit alpha (*PIK3CA*), *PTEN*, phosphoinositide-3-kinase regulatory subunit 1 (*PIK3R1*), Akt serine/threonine kinase 2 (*AKT2*), and *mTOR* [216, 217]. The overexpression of PI3K was found in more than 40% of NPC [218]. LMP1 and LMP2A activate the anti-TNF-related apoptosis ligand (TRAIL) activity, stimulate EMT, and promote the maintenance of cancer stem cell (CSC) phenotype, migration, and invasion via the PI3K pathways [219, 220]. Other than that, LMP1-activated PI3K/Akt/mTOR through carboxy-terminal activating region (CTAR)1 has promoted resistance in NPC and defected the DNA repair system by modulating the expression of human miR-21 and inhibiting the activity of forkhead box O3a (FOXO3a), respectively [221, 222]. In the view of PI3K playing key role in microtubule polymerization in mitosis, LMP1 could, through this pathway, promote the microtubule polymerization by enhancing the interaction of cell division control protein 2 (cdc2) with microtubule regulator, oncoprotein 18 (Op18/stathmin) [223]. Furthermore, mTOR pathway activated by LMP1 could also promote cell survival and invasion by inducing lipid synthesis [122]. Despite that, LMP2A-activated PI3K/Akt/mTOR/hypoxia-inducible factor 1-alpha (HIF-1α) also stimulated vasculogenic mimicry formation, which is usually found in advanced stage of tumor [124]. Hence, EBV-mediated PI3K activation is able to promote NPC progression, metastasis, radioresistance, microtubule dynamic, and vasculogenic mimicry.
  Nevertheless, the aberrant PI3K signalling can be abolished by using its therapeutic inhibitor. Suppression of PI3K/Akt/mTOR pathway has been found to successfully inhibit metastasis in NPC via mesenchymal epithelial transition (MET) [116]. For example, PI3K inhibitors such as 2-4-morpholinyl-8-phenlchromone, omipalisib, and gedatolisib are able to retard the cell survival and induce apoptosis in NPC cells [36, 38]. Cell cycle arrest and apoptosis were triggered when the PI3K/Akt/mTOR signalling was suppressed with euscaphic acid [117]. Besides direct targeting PI3K/Akt/mTOR, indirect suppression of its signalling as shown in Table 4 is another approach that can be utilized. Invasion, metastasis or CSC triggered by PI3K can be suppressed by targeting its upstream molecules, such as fibroblast growth factor 2 (FGF2), Flotillin 2 (Flot-2), sterol regulatory element-binding transcription factor 1 (SREBP1), DNA methyltransferase (DNMT) 1, epithelial cell adhesion molecule (EpCAM), and HIF-1α. Besides that, therapeutic resistance and EMT induced by PI3K can potentially be inhibited via targeting the collagen type I alpha 1 (COL1A), RBM3, regulator subunit p85, mitogen-activated protein kinase-activated protein kinase 2 (MK2), and vacuolar protein sorting 33B (VPS33B). Lately, several promising PI3K inhibitors, such as buparlisib (NCT0152787, NCT01737450), alpelisib (NCT01602315), and sonolisib, are currently undergoing active evaluation in clinical trials for advanced cases of head and neck cancer [224, 225]. In a Phase I clinical study, buparlisib exhibited a clinical response rate of 58.6% among patients with metastatic breast cancer [226]. Alpelisib is a PI3K inhibitor that specifically targets the α isoform, displaying strong antitumor efficacy while maintaining acceptable levels of toxicity in both in vitro and in vivo study [227]. Notably, it received Food and Drug Administration (FDA) approval in 2019 for its application in treating breast cancer [227]. The encouraging outcomes observed in this Phase I clinical study provide a strong basis for pursuing further investigation in the context of NPC. Continuing investigations are currently underway to explore the synergistic effects of these inhibitors in combination with other therapies for advanced NPC treatment. Illustratively, there are several ongoing Phase I clinical trials that examine combinations such as alpelisib with cetuximab and IMRT (NCT02282371), buparlisib with cetuximab (NCT01816984) or cisplatin (NCT02113878), as well as sonolisib with cetuximab (NCT01252628) for advanced NPC treatment. In a Phase I clinical trial, the combination of sonolisib and cetuximab demonstrated a well-tolerated toxicity profile. Additionally, it showcased promising anticancer efficacy, achieving a response rate of 44.4%, maintaining stable disease in 44.4% of cases, and observing disease progression in 11.1% of instances [224]. Moreover, an alternate study demonstrated an amplified radiosensitivity within a xenograft model when treated with a combination of PI3K and mTOR inhibitors (vistusertib with buparlisib or alpelisib) [228, 229]. Worth noting, although the PI3K/Akt/mTOR is typically reported in promoting tumorigenesis, but there are also few studies that demonstrated its activation have reduced the radioresistance and is negatively related to cancer progression [230, 231]. Therefore, further study exploring the role of PI3K/Akt/mTOR in NPC radioresistance and tumorigenesis is required.
- Targeting TP53
  TP53 (tumor protein P53) is a tumor suppressor gene (TSG) which plays a significant role in regulating cell cycle and apoptosis [232]. Mutated *TP53* disturbed the cell cycle and is associated with cancer progression, CSC phenotype, and EMT. This mutation is frequently found in other type of cancers but not in NPC [217]. Nevertheless, mutations in TP53 have been detected up to 70% cases of head and neck cancer [233]. Overexpression of TP53 in NPC has induced the activation of glycolysis and apoptosis regulator (TIGAR), which led to cell proliferation and invasion [14]. Its aberrant regulation was also linked to cisplatin resistance. Cisplatin resistance in NPC could be triggered after chemotherapy inhibiting the TP53 apoptotic signalling through the upregulation of miR-125a and miR-125b [134]. Apoptotic signalling of TP53 can be directly inhibited by interfering its mRNA 3′UTR with EBV miR-BHRF-1 and miR-BART5-3p [234, 235]. However, this TP53 aberration in NPC is also indirectly induced by LMP1 via the activation of NF-kB and activator protein 1 (AP-1) signalling, or the overexpression of its negative regulator, E3 ubiquitin-protein ligase murine double minute 2 (MDM2) [131, 232].
  TP53 was proposed as another target for NPC treatment. Upregulation of a TSG, a Pin2 telomeric repeat factor 1-interacting telomerase inhibitor 1 (*PinX1*), was found to activate the TP53/miRNA-200 axis, which in turn suppressed the EMT of CSC in NPC [133]. A recent study also demonstrated that the upregulation of miR-4270 in NPC has inhibited TP53 signalling [236]. In addition, cyclooxygenase-2 (COX-2)’s interaction with TP53 has been known to induce chemoresistance by inhibiting chemotherapeutic-induced cellular senescence [132]. Therefore, interrupting this interaction could restore the effectiveness of chemotherapy in inducing the cellular senescence. The oncogenic effect induced by TP53 can potentially be suppressed by targeting the molecules as shown in Table 5.
  Although numerous TP53 targeted therapies have been developed and examined in pre-clinical model over the past few decades, but none of them have been approved by FDA as of today. To date, only a few TP53 targeted therapies have been approved for clinical trials, such as nutlin-3, COTI-2, and idasanutlin. Nutlin-3 and idasanutlin are the small-molecules developed to compete with MDM2 for binding to the p53-binding pocket of MDM2. This allows the activation of TP53 and enhances the chemo-sensitivity [130, 131]. Preliminary result of treatment with combination of nutlin, cisplatin and deocetaxel in phase I clinical trial (NCT02508246) demonstrated promising antitumor efficacy in advanced head and neck cancer [237]. When tested in NPC cell line, C666-1 cells using combination of nutlin-3 with cisplatin, a growth inhibition and higher apoptosis in C666-1 cells were observed [238]. Besides that, idasanutlin has been suggested as second generation of nutlin with enhanced drug efficacy and reached clinical phase I/II stage testing in acute myeloid leukemia (NCT04029688) [239, 240]. The efficacy of idasanutlin in NPC has been demonstrated in preclinical models. This includes the activation of the TP53-dependent pathway, inhibition of cell growth, and the acceleration of apoptosis in an NPC xenograft model. [130]. Another TP53 targeted therapy using COTI-2 has illustrated growth inhibition effect in xenograft model by restoring the p53 conformation [241]. COTI-2 has reached phase I clinical trial (NCT02433626) as monotherapy or combination with cisplatin tested in head and neck cancer, however the result is currently unknown [242]. Collectively, the findings from these studies could support further clinical investigation as a potential therapy for NPC. Furthermore, novel immunotherapies could be discovered by studying the crosstalk between TP53 and the immune microenvironment in NPC.
- Targeting NF-κB
  NF-κB is constitutively activated in 90% of NPC cases through the somatic or frameshifts mutation in its negative regulators including NF-κB inhibitor alpha (*NFKBIA*), Cylindromatosis lysine 63 deubiquitinase (*CYLD*), tumor necrosis factor receptor-associated factor 3 (*TRAF3*), tumor necrosis factor-alpha-induced protein 3 (*TNFAIP3*), and NOD-like receptor family CARD domain containing 5 (*NLRC5*) [12, 70, 243]. Whole-exome, genome, and targeted DNA sequencing studies conducted on both primary and recurrent NPC cases have unveiled an upregulation of NF-κB in 40% of the instances [7, 217, 244, 245]. Generally, NF-κB signalling regulates various genes that are involved in cytokines and chemokines production. Hence, it plays a major role in inflammation, immune response, and cell proliferation. Aberrantly activated NF-κB could affect the TME, allow the immortalization of nasopharyngeal epithelial cells, maintain the CSC phenotypes, promote immunosuppression, and metabolic reprogramming [246]. The NF-κB signalling can be activated in an LMP1-dependent manner. LMP1-mediated NF-κB has promoted the NPC immortalization through the interaction between NF-κB subunit, p65, and human telomerase reverse transcriptase (hTERT), as well as downregulated the telomerase inhibitor, PINX1 [247–249]. In contrast to LMP1, LMP2A promoted the immunosuppression through the inhibition of NF-κB inflammation pathway [250]. The regulatory effect of both latent membrane EBV oncoproteins in NF-κB activity balances immunosuppression and sustained proliferation to enable the immortalization of NPC. LMP1-mediated NF-κB activation has promoted cell proliferation through the upregulation of provirus integration site for moloney murine leukemia virus 1 (Pim1). Furthermore, the NF-κB signalling can also be activated by caspase-12 (Casp12) through the activation of IκB kinase (IKK) (NF-kappa-B essential modulator) [251]. All of these strengthened the role of NF-κB in NPC pathogenesis. Hence, therapeutic targeting its positive regulators is imperative to enhance the therapeutic sensitivity.
  LMP1-mediated NF-κB has also downregulated the miR-203, which is important for EMT inhibition, invasion, and metastasis [135]. Aspirin, an NF-κB inhibitor was reported with its function to upregulate the miR-203 expression, in turn promoting MET [135]. Moreover, NF-κB is also indirectly associated with cisplatin resistance through the overexpression of bone marrow stromal cell antigen 2 (BST2) [140]. Upregulation of Sirtuin 6 (SIRT6) was reported to inhibit NF-κB [141]. Therefore, downregulating NF-κB through inhibition of BST2 and activation of SIRT6 could enhance the sensitivity to cisplatin. In addition, an inhibitor, andrographolide can be used to suppress the NF-κB target genes including *VEGF*, intercellular adhesion molecule 1 (*ICAM*), and matrix metalloproteinase (*MMP-9*) [138]. Besides that, NF-κB signalling can be directly inhibited by a statin, namely simvastatin [139]. Additionally, the knockout of cancer-related lncRNA such as NEAT1 has been reported as another therapeutic interest since it suppressed the miR-124 expression and upregulated NF-κB axis to promote proliferation and anti-apoptosis [142]. Upregulation of GTPase-activating protein (GAP) and deleted in liver cancer-1 (DLC-1) were shown to inhibit EMT and induce apoptosis through the suppression of EGFR/Akt/NF-κB cascade pathway [143]. Demethylation of ras-like estrogen-regulated growth inhibitor (RERG) has suppressed the ERK/NF-κB pathway and retarded the NPC progression by inhibiting the migration, invasion, and angiogenesis [144]. Targeting its upstream pathway or molecules such as EGFR/MEK/ERK/IKK/mammalian target of rapamycin complex 1 (mTORC1) could be another area of interest to focus [252]. As far as our understanding goes, there are scarce pre-clinical investigations and no clinical studies that have specifically aimed at targeting the NF-κB pathway as a therapeutic strategy in NPC. However, it's worth noting that there are small molecule inhibitors designed to target NF-κB and its upstream IKK complex, which have progressed to the clinical trial stage for the treatment of head and neck cancer. As abovementioned, IKK complex (comprising IKKα, IKKβ and IKKγ) mediates the dimerization of NF-κB. Notably, IKKβ serves as a pivotal catalytic subunit within this IKK complex [253]. Comparative study in NPC and nasopharyngitis revealed IKKβ positive expression rate is significantly higher in NPC compare to nasopharyngitis (56.7% vs. 33.3%) and is associated with shorted DFS rate (69.2% vs. 90.6%) [253]. IKKβ/NF-κB pathway has been suggested as one of the potential targets for NPC treatment. Acalabrutinib, a specific inhibitor of bruton tyrosine kinase that targets the upstream IKK Complex within the NF-κB pathway, has progressed to phase II of clinical trials (NCT02454179) [254]. In the phase II clinical trial, the combination of acalabrutinib and pembrolizumab was investigated in advanced head and neck cancer. However, the trial was terminated due to the lack of significant clinical benefit. When compared to pembrolizumab alone, the combination showed no substantial improvement, with median PFS at 2.7 months vs. 1.7 months and an overall ORR of 18% vs. 14% [254]. Nonetheless, conclusive findings could not be reached due to the restricted size of the sample population. Furthermore, an analysis of immune subsets within a similar clinical trial has demonstrated that acalabrutinib resulted in an improved immune response. This improvement encompassed augmented CD45^+^ leukocyte infiltration, alleviation of CD8^+^ T cell suppression, and an increase in memory response [254]. Additional comprehensive research is required to delve into the immune-related impacts of combining pembrolizumab and acalabrutinib in the context of TME. Another inhibitor targeted at NF-κB is xevinapant, a small molecule apoptosis antagonist. Its mechanism involves enhancing apoptosis by regulating the NF-κB signalling pathway [255]. Despite that, xevinapant also serves a role in re-establishing numerous caspase activities through the inhibition of various apoptosis inhibitors [256]. Recently, notable phase II results from a clinical study investigating the combination of xevinapant with cisplatin and IMRT in head and neck cancer have led to the designation of breakthrough therapy status [257]. Noteworthy enhancements were observed in outcomes with the inclusion of xevinapant, showcasing an ORR of 67% compared to 48% without, and a complete response rate of 52% compared to 38% without it [257]. With the encouraging data from this clinical trial, exploring the efficacy of acalabrutinib and xevinapant in NPC is anticipated. Moroever, a remarkable enhancement in survival and a substantial inhibition of tumor growth were observed with the combination of xevinapant and an anti-programmed cell death protein 1 (PD-1) [258]. Therefore, combinating these NF-κB targeted therapies with immunotherapy is another focus in NPC treatment.
- Targeting MAPK/ERK/MEK/JNK
  MAPK, ERK, and JNK pathways are abnormally activated in the tumors of certain NPC patients. These pathways play significant roles in various cellular processes and communications. It has been proposed that the increased expression of these signalling pathways plays a role in the onset of NPC. Research findings indicated that the overexpression of ERK protein in NPC is notably more pronounced compared to the levels observed in the normal nasopharyngeal mucosa (83.33% vs. 24.14%) [259]. Moreover, the expression rates of ERK, JNK, and MAPK, were found to be associated with TNM stage of NPC. Briefly, patients with N1-3 stage or M1 (83.85%, 100%) have higher expression level of ERK protein compared to N0 or M0 patients (75.00%, 82.54) [259]. Consistent finding was also observed in another study, where JNK expression was markedly higher in the advanced stages of NPC (III and IV) (89.2% ± 11.7%) compared to individuals in the early stages (I and II) (58.90% ± 4.90%) [260]. Subsequent investigations further substantiated these findings by revealing that the overexpression of JNK is more prevalent in patients with recurrence as opposed to those without recurrence. Moreover, patients showing notably lower JNK expression exhibited improved survival rates, with 1-year, 3-year, and 5-year overall survival rates of 100%, 90%, and 80%, respectively [260]. Collectively, this indicates that MAPK/ERK/MEK/JNK signalling is associated with NPC progression. Despite that, LMP1 overexpression is found to associated with dysregulation of these pathways. In LMP1-independent manner, MAPK can be deregulated by the mutation of its positive or negative regulators such as Erb-B2 receptor tyrosine kinase 3 (*ERBB3*), v-raf murine sarcoma viral oncogene homolog B1 (*BRAF1*), *FGFR2*, *FGFR3*, and neurofibromatosis 1 (*NF1*) [217]. Unusual activation of p38/MAPK has led to metastasis, angiogenesis, radioresistance, and poor prognosis [261]. Besides that, numerous studies have revealed that LMP1-mediated EMT was promoted via the activation of RAF/MEK/ERK pathway [262]. Nevertheless, LMP1-mediated JNK activation silenced the E-cadherin, inactivated p53, promoted DNA methylation, and contributed in telomerase activity [263, 264]. In addition to LMP1, LMP2A also mediated the activation of JNK pathway, thereby induced the aggressive phosphorylation of c-Jun which is commonly detected in advanced stage of NPC [260]. Taken together, the inhibition of these signalling pathways is very important for NPC therapeutic.
  Numerous studies have explored several strategies to inhibit or retard the effect of MAPK/ERK/JNK pathways activation. MAPK can be deactivated by knockdown of amyloid β precursor protein, which has been shown to retard the NPC progression [146]. Macrocyclic lactone antibiotic ivermectin (IVM) was reported to inhibit MAPK/ERK activator, P21 (RAC1) activated kinase 1 (PAK1), by preventing the phosphorylation of Raf1 and Mek [147]. Besides that, a tumor suppressor gene, zinc finger, MYNDtype containing 10 (BLU) was found to inhibit ERK signalling and its downstream effector (cyclins D1), thus promoted cell cycle arrest and apoptosis [151]. Overexpression of miR-124 inhibited the cell proliferation, migration, and invasion through regulation of transforming growth factor beta (TGF-β)/MALAT1//ERK axis [152]. Moreover, miR-483-5p also plays a role in activating ERK pathway through downregulation of death-associated protein kinase 1 (DAPK1) protein expression which resulted in increased colony formation, radioresistance, and DNA damage (broken double strand of DNA) [153]. Therefore, overexpression of miR-124, inhibition of miR-483-5p, or upregulation of BLU could increase radiosensitivity. Furthermore, since LMP1 required CTAR1 to activate ERK in order to induce EMT, interfering the signalling between CTAR1-LMP1 could inhibit the ERK activation [154].
  Currently, there are no ongoing clinical trials focusing on targeting these pathways for NPC. Instead, the majority of trials primarily concentrate on targeting the EGFR and the VEGF/VEGF receptor (VEGFR) pathways, as outlined in detail in “Recent clinical advancement of precision medicine in NPC” section. However, several MEK/MAPK inhibitors have entered clinical trial for head and neck cancers, such as cobimetinib (NCT00467779), selumetinib (NCT00085787), tremelimumab (NCT02586987), and TAK-733 (NCT00948467). Nevertheless, all of these clinical trials have failed to demonstrate therapeutic efficacy in the treatment of head and neck cancers [265, 266]. Among these endeavors, trametinib stands out with promising results. In a Phase II clinical trial (NCT01553851), trametinib administered as a neoadjuvant treatment showcased its effectiveness in significantly reducing tumor size, with reductions of up to 74% observed in all patients [267]. Furthermore, considering the intricate nature of oncogenic signalling pathways, employing combination therapies that target multiple pathways simultaneously could potentially yield improved clinical outcomes. For example, the combination of MEK inhibitor trametinib with BRAF inhibitor dabrafenib has demonstrated an enhanced therapeutic response, and the PFS was observed [268]. Trametinib is currently used to treat metastatic melanoma and serves as a combination therapy for managing advanced and metastatic solid tumors [269, 270]. These clinical findings offer crucial scientific evidence, suggesting the necessity to explore potent MEK/MAPK inhibitors in precision trials for the effective treatment of NPC. Continued investigation into the interaction between MAPK/ERK/MEK/JNK pathways and the TME is essential to formulate effective therapeutic strategies.
- Targeting JAK/STAT
  Previous studies have investigated the role of JAK/STAT3 in NPC initiation, progression, and metastasis, as well as associated with advanced stages of NPC [271, 272]. The activation of STAT3 has been detected in 70.5% of NPC patients, and LMP1 has been suggested to play role in this activation by enhancing its dimerization and DNA binding in B-cells [271, 273]. A research discovery unveiled the expression pattern of JAK/STAT3 in both NPC and chronic nasopharyngitis. Notably, the JAK/STAT3 signalling exhibited markedly elevated levels in NPC (60.2% and 70.9%), contrasting with the figures observed in chronic nasopharyngitis (12.8% and 14.1%) [274]. Moreover, high activation of this pathway is associated with poor prognosis [271, 272]. NPC patients with elevated JAK2/STAT3 expression experienced notably reduced survival times. Specifically, the median survival for patients with no expression of JAK2 or STAT3 was 58.7 ± 5.3 or 53.6 ± 13.1 months, while those with positive expression of JAK2 or STAT3 had median survival times of 33.6 ± 19.7 or 39.5 ± 20.8 months, respectively [274, 275].
  In NPC, this pathway is constitutively activated by LMP1 through CTAR3 activation and upregulation of IL-6 [276–278]. In fact, STAT is activated in a positive feedback loop manner, whereby STAT activation signalling upregulates the expression of LMP1, in turn induces the LMP1-mediated IL-6 production and results in activation of STAT [277]. Abnormal activation of this pathway contributed to aggressive characteristic of NPC including angiogenesis, migration, EMT, anti-apoptosis, cell proliferation, and metastasis [279]. Upregulation of STAT pathway was also positively linked to VEGF expression and associated with poor survival in NPC patients [274]. Among the STAT family proteins, STAT1, STAT3, and STAT5 are the most commonly associated with NPC [280, 281].
  Generally, JAK/STAT pathways can be therapeutic targeted through several strategies. For example, the upregulation of Raf kinase inhibitory protein (RKIP) can potentially supress the activation of STAT pathway [157]. Moreover, STAT pathway can be inhibited by using the regulator miRNA, such as miR-29a and miR124-3p, which can interfere the 3′UTR of STAT3 mRNA [158, 160]. With this, the expression of B-cell lymphoma 2 (BCL-2) which plays a role in promoting cell proliferation can be supressed, in turn enhanced the chemo- and apoptotic sensitivity [282]. Furthermore, lncRNA LINC00669 was found to promote the cell proliferation and invasion in NPC through the upregulation of JAK/STAT signalling [283]. Therapeutic targeting LINC0669 was proposed as a potential treatment for NPC with aberrant JAK/STAT oncogenic signalling.
  Numerous inhibitors have been developed to supress STAT3 activation, such as peptidomimetics, PM-73G, ISS-610, and tyrosine-phosphorylated peptide, phosphopeptide inhibitor (PY*LKTK) which function to interfere its DNA binding and dimerization [284]. A phase 0 clinical study (NCT00696176) on STAT3 decoy oligonucleotide observed supressed level of STAT3 expression and retarded cell proliferation in head and neck squamous cell carcinomas [285]. Stattic, a STAT inhibitor, has demonstrated encouraging outcomes in enhancing chemosensitivity and radiosensitivity across various cancer types, including NPC [286–288]. In an in vitro NPC model, the application of a combination treatment involving stattic, cisplatin, and radiation resulted in growth inhibition and heightened apoptosis [288]. However, these JAK/STAT targeted therapies are currently in the preclinical stage, and there is still an inadequate understanding of their mechanisms of action. Hence, further experimental research is necessary to gain a deeper insight into the underlying mechanisms.
  Nonetheless, it's worth noting that a few JAK/STAT inhibitors, such as ruxolitinib and danvatirsen, have progressed to the clinical trial stage for the treatment of head and neck cancer and may be beneficial for NPC treatment. Ruxolitinib, which has demonstrated growth inhibition in both two-dimensional and patient-derived xenograft models, is currently in the recruitment phase (NCT03153982) to assess its effectiveness in treating head and neck cancer [289]. Furthermore, ruxolitinib has also gained the approval for myelofibrosis treatment [290]. Apart from that, danvartisen, an antisense oligonucleotide molecule that targets STAT3 by intefering mRNA translation, is being examined in clinical trials (NCT02499328) involving cases of metastatic head and neck cancer. This trial aims to explore its potential as a monotherapy or in combination with MED14736, an immunotherapy that disrupts the interaction between PD-1 and PD-L1 [291]. The clinical investigation has shown elevated anticancer activity resulting from the combination of a PD-L1 inhibitor with a STAT3 inhibitor, outperforming the effectiveness of PD-L1 monotherapy [291]. Therefore, further research is warranted to investigate the potential of these JAK1,2/STAT3 inhibitors and their combination with PD-L1 immunotherapy as a treatment strategy for NPC. In addition, delving into the correlation between JAK/STAT activation and metabolic reprogramming in NPC could reveal potential metabolic targets that could be harnessed for therapeutic purposes in NPC treatment.
- Targeting Wnt/β-catenin pathway
  NPC patients’ tumors with aberrant Wnt/β-catenin signalling are associated with CSC phenotype and radioresistance [292]. The aberrant upregulation of Wnt/β-catenin signalling was observed in NPC through the examination of the expression of canonical WNT ligands, such as WNT8B. In the study, a substantial 75.6% of NPC cases were exhibited high expression of WNT8B [293]. Subsequent observations of NPC patients with overexpressed WNT8B revealed a correlation with shorter survival durations. Among patients displaying overexpression of WNT8B, the median survival was 37.0 ± 3 months, whereas those with no expression of WNT8B had a median survival of 52.3 ± 7 months [293]. A comparable pattern was also reported in another study, wherein 60.4% of NPC patients showed upregulation of β-catenin expression. Among individuals with β-catenin overexpression, there was a decrease in rates for OS, distant metastasis-free survival (DMFS), locoregional recurrence-free survival (LRFS), and DFS in contrast to the group with low expression (49.2% vs. 48.7%, 64.2% vs. 65%, 62.5% vs. 62.4%, and 37.5% vs. 39.3%, respectively) [294]. Moreover, within the same study, among NPC patients exhibiting overexpression of β-catenin expression, 39% of them experienced mortality attributed to factors such as local recurrence (43.8%), distant metastasis (46.9%), or other causes (9.4%) [294]. A comprehensive meta-analysis that included eight studies has unveiled a significant association between β-catenin overexpression and adverse OS (HR = 2.45, 95% CIs 1.45–4.16, p = 0.001), as well as a reduction in DFS or PFS (HR 1.79, 95% CIs 1.29–2.49, p = 0.000) [295]. In short, the overexpression of Wnt/β-catenin signalling has been significantly associated with advanced disease stages and unfavorable survival outcomes in NPC patients.
  Similarly, Wnt/β-catenin pathway can be deregulated by EBV infection. LMP2A induces the phosphorylation of glycogen synthase kinase 3β (GSK3β) via the stimulation of metastatic tumor antigen 1 (MTA1) signalling, consequently leads to mass nuclear translocation of β-catenin [296]. Besides that, miR-150 also inhibits the GSK3β expression by targeting its 3′UTR, thereby results in accumulation of β-catenin to promote radioresistance, EMT, and invasion [297]. Further research into potential therapeutic approaches targeting the Wnt/β-catenin pathway is imperative to improve the survival prospects for individuals diagnosed with NPC. Up to date, only three drugs specifically targeting the Wnt/β-catenin signalling pathway, namely niclosamide, sulindac, and pyrvinium, have received FDA approval for the treatment of conditions such as ovarian cancer, colon cancer, and intestinal polyposis [298–300].
  Several studies have aimed to counteract the oncogenic effect in NPC which is imparted via Wnt/β-catenin pathway by targeting its carboxyl terminus or co-activator, CREB binding protein (CBP). Transcription and nuclear translocation of β-catenin can be repressed by the binding of a small nuclear protein, Chibby (Cby) to its c-terminal and 14-3-3 adaptor proteins [163]. An antagonist of CBP/β-catenin, referred to as ICG-001, has been created to inhibit the transcriptional activity of CREB binding proteins. This inhibition is accomplished by ICG-001 binding to cyclic AMP response element-binding protein [301, 302]. Consequently, this approach has resulted in the suppression of gene transcription that encourages the proliferation and self-renewal of stem cells, leading to a reduction in the population of CSC-like entities, which are implicated in drug resistance. Thus, ICG-001 also holds the potential to augment drug sensitivity. The synergistic effect of combining ICG-001 with the chemotherapy drug cisplatin was investigated in both an in vitro three-dimensional model of EBV-positive C666-1 and an in vivo xenograft model. This combination demonstrated a notable suppression of tumor growth in both models [161]. In contrast to the effects of cisplatin alone, the combination of cisplatin with ICG-001 exhibited an intensified tumor suppression effect and improved overall health condition. These findings serve as evidence of the efficacy of ICG-001 as a potential drug targeting CSCs and highlight the synergistic benefits of combining ICG-001 with conventional therapies, ultimately enhancing treatment outcomes in NPC [161]. These promising findings may justify the need for further clinical studies involving NPC patients. Currently, ICG-001 has progressed to a phase I clinical trial (NCT01302405) involving patients with advanced solid tumors. Another CBP/β-catenin antagonist, known as PRI-724, has been developed with a potency 20 times greater than that of ICG-001. Currently, PRI-724 is undergoing investigation in a variety of clinical trials. Briefly, there is a phase Ia clinical study (NCT01302405) assessing the toxicity profile of PRI-724 in advanced solid tumors, a phase Ib trial (NCT01764477) examining its efficacy in combination with gemcitabine in refractory pancreatic cancer treatment, and a phase I/II trial (NCT01606579) studying the safety and efficacy in leukemia treatment. Both pre-clinical and clinical evidence substantiates the safety and effectiveness of CBP/β-catenin antagonists when combined with conventional drug treatments for cancer [161, 303]. Hence, future clinical investigations focusing on the combination of PRI-724 with cisplatin could offer potential benefits for NPC patients, potentially leading to improved treatment outcomes. Other molecules that have shown efficacy in intefering with β-catenin complex or impeding it transcription, such as vantictumab, XAV939, WNT974, JW55, and iCRT3 in other cancer types, could also be explored for their potential effectiveness in treating NPC [304–310].
  Apart from that, numerous fundamental studies have been conducted to target Wnt/β-catenin pathway in NPC. Leucine-rich repeat-containing G protein-coupled receptor 5 (Lgr5) which acts as Wnt receptor activates the Wnt/β-catenin signalling to promote the EMT and chemoresistance [167]. Hence, Lgr5 was suggested as another interesting candidate to suppress the Wnt/β-catenin signalling. Other than that, targeting miRNA which is involved in the regulation of Wnt/β-catenin pathway including miR-124, miR-506, and miR-34c have been explored. Downregulation of miR-124 was observed in NPC, while the upregulation of miR-124 has suppressed the proliferation and invasion in NPC cells via the inhibition of Wnt/β-catenin signalling by targeting Calpain small subunit 1 (Capn4) [164]. Activation of LIM Homeobox 2 (LHX2) through the Wnt/β-catenin signalling is required for NPC metastasis and EMT, whereas upregulation of miR-506 has inactivated the Wnt/β-catenin signalling and led to inhibition of LHX2 [165]. Study showed that Wnt/β-catenin-conferred radioresistance could be reversed by external delivery of miR-34c which suppressed the β-catenin expression [162]. Moreover, silencing of FOXO3a in NPC was also associated with Wnt/β-catenin mediated radioresistance, thereby its activation could enhance the radiosensitivity in NPC patients [166]. Further investigations into these therapies targeting the Wnt/β-catenin pathway in NPC patients are warranted. Conducting comprehensive research into the interaction between the Wnt/β-catenin pathway and the immune microenvironment has the potential to yield valuable insights, which could in turn advance immunotherapy strategies for NPC.

#### Aberration in DNA methylation and histone modification

Distinct epigenetic alterations derived by EBV infection such as DNA methylation and histone modification which contributed to NPC pathogenesis were reported. Overall, common methods employed for assessing demethylation and gene re-expression include methylation-specific polymerase chain reaction, bisulfite genomic sequencing, immunohistochemistry, and chromatin immunoprecipitation.

Aberrant DNA methylation downregulates the TSGs that play important role in various biological processes. In NPC, TSGs such as Ras association domain family member 1 (*RASSF1A*), cyclin-dependent kinase inhibitor 2A (*CDKN2A*), *BLU,* and deleted in lung and esophageal cancer protein 1 (*DLEC1*) which are involved in regulating the DNA repair and cell proliferation were silenced by promoter hypermethylation [25, 311, 312]. Besides that, checkpoint with forkhead and ring finger (CHFR) which regulates the mitotic checkpoint and MMP-19, promotes anti-cancer effect and suppresses angiogenesis; it was also found to be inhibited by aberrant promoter hypermethylation in NPC [313, 314]. The downregulation of MMP-19, which is known to contribute to NPC tumorigenesis through enhancing invasion and metastasis, was observed in up to 70% of early NPC [313]. However, the upregulation of ten–eleven translocation methylcytosine dioxygenase 1 (TET1) has been reported to suppress tumor cell survival, migration, and invasion via the activation of Wnt antagonist, dishevelled binding antagonist of beta catenin 2 (DACT2), and SFRP2. Nevertheless, in NPC, TET1 expression was inhibited by methylation [315].

Recent developments in next-generation genome sequencing (NGS) and array-based methylome studies have revealed significant changes in the host cell methylome in NPC, characterized by cytosine-phosphate-guanine (CpG) hypermethylation [316, 317]. EBV latent protein LMP1 has also been found to involve in modulating the epigenetic modification through its interaction with DNA methyltransferases (DNMTs) or demethylases. For example, research has provided evidence that LMP1 triggered hypermethylation and silencing of the tumor suppressor phosphatase and tensin homologue (PTEN). This occurs through the activation of DNA methyltransferase 3 beta (DNMT3b) via the NF-κB signalling pathway, where the NF-κB p65 subunit binds to the DNMT3b promoter [318]. Moreover, the LMP1 transfected EBV-negative NPC cells have showed an elevation of LMP1 mediated DNMT3b expression [318]. Another study have showed that LMP1 inducing hypermethylation in *E-cadherin* genes through the activation of DNMT1 [264]. Activation of DNMT1 was found to be initiated by LMP1-mediated AP-1/JUN signalling [264, 319]. Additionally, further investigation has demonstrated that in the presence of LMP1, the levels of all three DNMT enzymes (DNMT1, DNMT3a, and DNMT3b) are increased [320]. Moreover, there are various genes in the aforementioned cellular signalling pathways that were also interfered by promoter hypermethylation and contributed in NPC tumorigenesis. In a gene expression profiling study of NPC initial tumor, the promoter of Wnt signalling inhibitor including secreted frizzled related protein 1 (*SFRP1*), *SFRP2*, *SFRP4*, *SFRP5*, Wnt inhibitory factor 1 (*WIF1*), and dickkopf WNT signalling pathway inhibitor 1 (*DKK1*) were turned off by DNA methylation, thus leading to abnormal activation of Wnt signalling and its downstream components [321]. Consistent findings from another study have reported that 67–95% of 40 NPC cases had methylation in *SFRP1*, *2*, *4* and* 5*, and *DKK2* [317]. In addition, these promoter methylations often occur in numerous chromosomal regions such as 3p21.3, 9p21, and 6p21.3. Apart from that, a more comprehensive analysis of 9 studies on methylation in NPC showed that p16 hypermethylation occurs in 41.1% of NPC [322]. Furthermore, *RAFFS1*, multiple genes including zinc finger MYND-type containing 10 (*ZMYND10*), leucyl-TRNA synthetase 2, mitochondrial (*LARS2*), MutL Homolog 1 (*MLH1*), lactotransferrin (*LTF*), and *DLEC1* in 3p21.3 region were promoter methylated in the tumors of certain NPC patients [316]. *CDKN2A* and *CDKN2B* located in 9p21 were also inactivated by promoter methylation [25]. The transcriptional silencing of *CDKN2A* via promoter hypermethylation has been identified in over 80% of NPC cases. This phenomenon is suggested to be a significant mechanism that interferes normal cell cycle regulation and facilitates persistent EBV infection in NPC [323]. Besides that, *BLU* and *DLEC1* were detected in 40–70% of NPC, and methylation in both gene has led to dysregulation of cell cycle, stress-response, and STAT3 pathways [324–326].

Histone modification involves the modification of histones N-terminal tails by several post-translational mechanisms including ubiquitination, methylation, acetylation, sulfonylation, and phosphorylation. This modification often occurs in side chain of lysine residues on histone H3. Histone acetylation and deacetylation are important in mediating the expression of host gene and cellular processes [327]. Dysregulation of histone bivalent switch including H3K4me3 (activation mark) and H3K27me3 (suppression mark) has immortalized the NPC cells. In NPC, histone modification suppresses the DNA repair gene and regulates the expression of EBV genes, especially histone deacetylation (HDAC) which is involved in regulating EBV latency and EBV-associated tumorigenesis [328]. Study showed that enhanced level of histone 3 lysine 9 trimethylation (H3K9me3) was associated with NPC metastasis, and chemo- and radio-resistance [329]. Besides that, LMP1 was found to upregulate the H3K27ac signal, in turn upregulated the oncogene translocation-Ets-leukemia virus and activated NFκB pathway [330]. Studies also proposed that EBNA1 has mediated this aberrant histone modification by reducing the activation of H3K4me3 and promoting the activation of H3K27me3 in DNA damage repair gene [331, 332]. In addition to these two bivalent switches, aberrations in other histone marks such as H3K27ac, H3K26me3, and H3K9me3 were also suggested for their synergetic role in abnormal regulation of the host genes expression [332].

Recent study has demonstrated that P53 and p21 were typically methylated in NPC, and restoration of its expression via demethylation of DNMT3B was shown to reverse EMT and induce apoptosis [333]. Furthermore, protocadherin 17 (PCDH17) was methylated in 100% of NPC cell lines, while demethylation of PCDH17 or knockout of DNMT has inhibited the NPC oncogenesis [334]. Moreover, CCAAT enhancer binding protein alpha (CEBPA) expression that was required to inhibit TGF-β-mediated EMT was downregulated in NPC by LMP1 through the activation of STAT5 and suppression of histone acetylation via recruitment of HDAC to CEBPA locus [335]. CEBPA was also found silenced in LMP1 negative NPC [335]. Study conducted by Xie’s team has reported that EMT can be reversed by restoring CEBPA expression through the inhibition of HDAC [335]. In addition, inhibition of HDAC could also potentially suppress the oncogenic-activated NF-κB pathway in NPC [336].

The oncogenic epigenetic alterations observed in NPC tumors have the potential to be reversed by employing specific inhibitors tailored to target these changes. For example, small molecule inhibitors can be developed to target the epigenetic enzyme involved in histone modification and hypermethylation, such as histone acetyl transferases (HATs), HDACs, and DMNT1. Recently, HDAC inhibitors like romidepsin, vorinostat, and chidamide have received clinical approval for the treatment of conditions such as cutaneous T-cell lymphoma, peripheral T-cell lymphoma, and multiple myeloma [337–340]. Agents that target DNMTs, such as 5‐azacytidine and decitabine, have also been developed. Numerous pre-clinical and clinical studies have provided evidence of the efficacy of HDACs and DNMTs inhibitors in NPC.

The DNMT inhibitor, 5‐azacytidine, has shown its efficacy in restoring the expression of E-cadherin and inhibiting cell migration in NPC [320]. A significant inhibition of tumor growth was observed when a combination of 5-azacytidine and radiotherapy was administered [341]. These findings indicated that 5-azacytidine enhances the radiosensitivity of NPC cell lines and xenograft models, leading to an increased apoptosis in irradiated cells [341]. The combination of 5-azacytidine with radiotherapy may represent an attractive alternative strategy for NPC treatment. An orally bioavailable formulation of 5-azacytidine, known as CC-486, has been developed and has demonstrated the potential to enhance treatment outcomes when combined with radiotherapy in pre-clinical NPC models. This formulation is currently undergoing clinical trials for the treatment of NPC. In a phase I clinical trial (NCT01478685), CC-486 was administered alone and in combination with carboplatin or nab-paclitaxel, resulting in three partial responses and four cases of stable disease among eight NPC patients [342]. However, combining CC-486 with cytotoxic chemotherapy did not significantly enhance response rates [342]. The clinical activity of CC-486 as a monotherapy in NPC has provided the foundation for advancing to a phase II clinical trial. However, the results of the phase II clinical trial (NCT02269943), which included 36 patients with locally advanced or metastatic NPC, did not demonstrate significant clinical benefits. The trial reported an objective response rate (ORR) of 12% and a median progression-free survival (PFS) of 4.7 months [343]. Although the response rates were low, it is worth noting that among the 25 evaluable patients, 19 of them (76%) exhibited some degree of response [343]. The disease control rate (DCR) of 52% was actually better than expected, especially considering that the patient population consisted mainly of individuals with heavily pretreated metastatic NPC [343]. These findings suggest the possibility that CC-486 primarily exerts its effects by stabilizing the disease rather than inducing substantial tumor regression. Moreover, the toxicity of CC-486 was overall well tolerated. Decitabine, another DNMT inhibitor, is also undergoing clinical investigation in a phase I/II clinical trial (NCT03701451), where 30 NPC patients were received treatment with decitabine in combination with cisplatin-induced chemotherapy, followed by concurrent chemoradiotherapy.

HDAC inhibitors, specifically Vorinostat and Romidepsin, have been found to effectively suppress NPC proliferation both in vitro and in vivo [335, 344, 345]. Synergistic cytotoxic effects were observed when combining HDAC inhibitor with chemo-drugs or radiation [346]. The combination of Abexinosta with either cisplatin or irradiation could potentially reduce treatment resistance in preclinical NPC model [346]. Study has also examined HDAC inhibitor in NPC metastasis in vivo. The increased expression of deoxynucleotidyltransferase terminal-interacting protein 1 (DNTTIP1) has been shown to facilitate NPC tumorigenesis by inhibiting the expression of the *dual specificity phosphatase 2* (*DUSP2*) gene. This inhibition occurs through the recruitment of HDAC1 to the promoter region of *DUSP2*, thereby preserving a deacetylated state of histone H3K27 [347]. The decreased expression of DUSP2 leads to the abnormal activation of the ERK signalling pathway and increased levels of MMP2, which in turn facilitate the metastasis of NPC. Research has reported that the class I HDAC inhibitor Chidamide plays a role in increasing the levels of H3K27-Ac and DUSP2 while decreasing the levels of DNTTIP1, p-ERK, and MMP2. This action inhibits the proliferation, migration, and invasion of NPC cells in both in vitro and in vivo models [347]. These initial findings indicate that HDAC inhibitors, particularly in combination with chemotherapeutic agents, hold a great promise for the treatment of NPC. A phase I clinical trial (NCT00336063) is currently assessing the impact of combining vorinostat and 5-azacitidine in recurrent or metastatic NPC patients, but the results of this trial have not been reported yet. Moreover, further clinical investigations into the use of other HDAC inhibitors in the treatment of NPC are warranted.

Likewise, the use of DNMT and HDAC inhibitors is believed to reactivate the host immune response by reversing the methylation-induced gene silencing. This effect is particularly significant in EBV-associated tumors, as it can reactivate silenced immunodominant antigens in infected cells [348]. Such an approach could potentially create a more conducive microenvironment for immunotherapies by improving the presentation of tumor-specific antigens. This, in turn, could lead to the expansion of both primary and adaptive immune responses against the tumor. In a phase I study, changes in several EBV promoters were investigated at the molecular level before and after 5-azacitidine treatment in patients with NPC. The study revealed varying degrees of demethylation in all latent and early lytic EBV promoters following treatment [349]. Given the potential clinical benefits observed with CC-486 as monotherapy in clinical studies, exploring the combination of CC-486 with immune checkpoint inhibitors holds promise as an area for further clinical investigation. Therefore, further investigation into the crosstalk between different epigenetic modifications, such as H3K9me3, H3K27ac, and H3K26me3, and their collaborative impact on the control of host gene expression may pave the way for more accurate and effective epigenetic-targeted treatments.

#### Aberration in immunomodulatory components

The immune environment of NPC is characterized by the intense filtration of tumor-infiltrating immune cells (TIICs). Despite that, there is a supressed immune response, and the presence of immunosuppressive infiltrates such as regulatory T-cells (Tregs), M2 macrophages, and myeloid-derived suppressor cells, leads to immunotolerance, thereby promoting tumor progression [350–352]. The detailed discussion of each immunomodulatory component in NPC contributing to immune evasion can be found in our previous review [353].

Immune evasion is the crucial key for tumor cells to survive. In EBV-positive NPC, many EBV oncoproteins have assisted in immune evasion by suppressing the immune cells through the modulation of cytokines, immune checkpoint regulators as well as tumor associated exosomes in TME [354, 355]. As aforementioned, the overexpression of LMP1 is able to activate several cellular signalling pathways including NF-κB and STAT3 to induce inflammatory responses. Hence, due to its immunomodulatory properties, LMP1 was suggested as the main EBV oncoprotein that can promote immune evasion as well as NPC tumorigenesis.

Studies reported that EBV-positive NPC patients released the LMP1-derived exosome which was able to exert its immunosuppressive and oncogenic effect to the surrounding stromal cells in TME [356–358]. Therefore, LMP1 is capable of modulating the stromal cells and tumor infiltrating leukocytes (TILs) response in TME to promote the immune evasion and NPC progression by several strategies, including (1) suppressing natural killer- and T-cells by inducing IL-18 and IP-10 [359]; (2) mediating programmed cell death protein 1 (PD-1)/programmed death-ligand 1 (PD-L1) signalling through NF-κB, STAT3, and AP-1 pathways to suppress the TILs action [360]; (3) promoting differentiation and expansion of myeloid-derived suppressor cells by upregulating the production of IL-1β, IL-6, IL-18, and granulocyte–macrophage colony-stimulating factor (GM-CSF) via the activation of aerobic glycolysis [361]; (4) suppressing the innate immune response by sumoylation of interferon regulatory factor 7 (IRF7) [362]; (5) affecting the cell–cell interaction by regulating the cell adhesion molecules ICAM-1, CD18, and lymphocyte function associated antigen (LFA) [359]; (6) disturbing the antigen processing and presentation by modulating the expression of transporter associated with antigen processing (TAP), major histocompatibility complex (MHC) class I and II molecules [363]; (7) creating chronic inflammatory infiltrate by regulating expression of immunomodulatory cytokines [359]; (8) regulating the expression of COX-2, VEGF, EGFR as well as HIF-1α [124, 355]; and (9) inducing apoptosis in T-helper-type 1 (Th1) cells via the regulation of galectin-9 (Gal-9) expression in NPC exosome [364]. In addition, LMP2A and B also inhibited the anti-tumor immunity by suppressing the interferon (IFN) signalling [365]. Furthermore, exosomes derived from LMP1 have the capability to upregulate the MAPK/Akt signalling pathway, thereby promoting cell proliferation and inhibiting the differentiation of B cells into antibody-secreting cells. Exosomes derived from NPC containing C15 have the ability to convert CD4^+^CD25^−^ T lymphocytes into Treg cells, promoting Treg cell aggregation and immunosuppressive effects. Consequently, this allows tumor cells to evade immune clearance [366].

Besides EBV oncoproteins, its miRNAs including miR-BART and miR-BHRF are also participating in immune evasion in NPC. MiR-BART6-3p and miR-BART16-5p were found to suppress the type-1 IFN signalling by interfering the retinoic acid-inducible gene I (RIG-I) mRNA and CREB-binding protein mRNA, respectively [367, 368]. MiR-BART1, miR-BART2, and miR-BART22 have also been shown to suppress the maturation of T-cells, and reduce the activation of cytotoxic T lymphocytes (CTLs) and natural killer (NK) cells by downregulating the production of interleukin (IL)-2 [369]. Moreover, miR-BART2-5p repressed the expression of stress-induced NK cell ligand, MHC class I polypeptide-related sequence B (MICB), thus enabling the EBV infected cells to escape from immune recognition [370]. Besides that, the antigen processing and presentation are also disturbed by miR-BART17 and miR-BHRF1-3 via targeting the antigen processing protein, Cathepsin B and antigen peptide transporter 2 (TAP2) [371]. Worth noting, EBV EBER1 is also capable of upregulating the anti-inflammatory and growth promoting IL-10 through the binding with RIG-I receptor and activation of interferon regulatory factor 3 (IRF-3) [372]. Furthermore, the anti-tumor immune response was also found to be repressed by the overexpression of TAP inhibitor, EBV BNLF2a. Uncovering the distinct mechanism of immune evasion among the NPC patients may help to explore the NPC therapeutic strategies through the restoration of anti-tumor immune response. For example, EBER-induced upregulation of IL-10 secretion in NPC can be blocked through indirect or direct knockdown of IRF-3 or IL-10, small interfering RNA (siRNA), respectively [373, 374]. Both reverse transcription PCR and enzyme-linked immunosorbent assay (ELISA) results have shown that the knockdown of RIG-I led to a decrease level of IL-10 [373, 374].

Nowadays, PD-1/PD-L1 checkpoint is the most common immunecheckpoint axis observed in NPC, with up to 80% of NPC are observed with PD-L1 expression [375–378]. A study has reported that EBV-positive NPC exhibited higher levels of CD3, CD4 and CD8^+^ TILs compared to EBV-negative NPC [379]. Additionally, these CD8^+^ cells often expressed high levels of PD-L1. Other immune-suppressive cell types, including forkhead box P (Foxp) 3^+^ Treg and CD68^+^ tumour-associated macrophages (TAMs), also exhibited higher levels in EBV-positive NPC [380, 381]. Aside from LMP1, research has revealed that interferon-gamma (IFNγ), such as IFNβ, plays a role in stimulating the expression of PD-L1 and PD-L2 in NPC cells [382]. Hence, therapeutic targeting of the PD-1/PD-L1 axis in EBV-positive NPC could enhance cytotoxic responses, potentially reversing T-cell exhaustion and restoring their anti-tumor functions. To date, a wide array of immunotherapies targeting the PD-1/PD-L1 axis has been explored in the treatment of various types of neoplasms. These therapies include anti-PD-1 antibodies like nivolumab, cemiplimab, pembrolizumab, and anti-PD-L1 antibodies such as atezolizumab, durvalumab, and avelumab [383, 384]. Anti-PD-1 antibodies, including nivolumab and pembrolizumab, have shown promising antitumor efficacy in individuals with recurrent and metastatic NPC [385, 386]. Among patients who received nivolumab treatment, an ORR of 20.5% was observed, along with a one-year OS rate that exceeded historical data for comparable patient groups [386]. Moreover pembrolizumab was recently evaluated in a clinical trial (NCT02339558), with the results showing a deceased in lesion size in 66% of the patients and a DCR of 77.8% [387]. In a single-arm and multicenter phase II study (CAPTAIN study) assessing the safety and efficacy of camrelizumab [244], recurrent and metastatic NPC patients achieved an ORR of 34.00%, a DCR of 59.00%, and a median PFS of 5.6 months. The most frequently encountered adverse effects of this treatment are stomatitis, anemia, and abnormal liver function. Additionally, two randomized clinical trials, NCT02605967 and the KEYNOTE-122 study, were performed to assess the efficacy and safety of immune checkpoint inhibitor monotherapy compared to chemotherapy [388, 389]. Nevertheless, there were no significant differences observed in ORR and DCR, indicating that the moderate anti-tumor efficacy of PD-1 antibody monotherapy is comparable to that of chemotherapy. However, there was a notable reduction in the toxicity profile when utilizing PD-1 antibody monotherapy. Nonetheless, there are numerous limitations of using PD-1/PD-L1 inhibitors as monotherapy, primarily the limitation of anti-tumor response rate [390, 391]. The emergence of resistance to PD-1/PD-L1 inhibitors is another significant concern. A study revealed that approximately 30% of patients initially respond well to treatment but eventually develop acquired resistance, leading to a loss of response to this form of immunotherapy [392, 393]. Therefore, other immune checkpoint such as lymphocyte activating 3 (LAG3), T-cell immunoglobulin mucin-3 (TIM3), and T cell immunoreceptor with Ig and ITIM domains (TIGIT) and cytotoxic T lymphocyte-associated protein 4 (CTLA4) were also studied as therapeutic target in NPC. Studies showed that some CD8^+^ T-cells expressed high level of genes encoding immunosuppressive checkpoint proteins TIM-3 and LAG3, as well as populations of CD4^+^ T-cells expressing genes encoding TIGIT and CTLA4 [394]. The Galactin-9/TIM-3 axis was also found to contribute to an immune-suppressive TME in NPC, as Galectin-9 is highly expressed in tumor cells and higher levels of Tim-3 Foxp3^+^ lymphocytes are found in paired NPC primary and recurrent tissues. Inhibition of TIM3 ligand, Galectin-9, was found to revigorate infiltrating T lymphocytes through interfering with the interaction between PD-1 and TIM3 [395]. Hence, this was suggested as alternative approach to overcome the resistance to the PD-1/PD-L1 inhibitors.

The EBV oncoproteins and miRNAs, especially LMP1, that contributed to numerous oncogenic effects in EBV-NPC patients can be suppressed by blocking these EBV-associated signalling. Consistent studies have demonstrated that knockdown of LMP1 using DNAzymes such as DZ509 and DZ1 has successfully inhibited the NPC cells growth and restored the anti-tumor effect [178, 396]. Hence, direct targeting of the viral oncoproteins or miRNAs in EBV-positive NPC patients was proposed as an attractive target for therapeutic development for EBV-positive NPC.

Meanwhile, immunotherapy that precisely targets EBV has been proposed as the most promising approach for NPC treatment. This includes (1) therapeutic vaccine constituted of EBV latent antigens; (2) infusion of EBV processed CTLs; and (3) antibodies targeting EBV latent antigens such as human antibody Fab conjugated with mitomycin C (HLFAFab-MCC) targeting extracellular domain of LMP [101, 397, 398]. Adoptive therapy, a therapeutic strategy that uses immune cells including CTLs and cytokine induced killer cells that directly and preferentially target cancer cells, has been explored in NPC treatment. Considering that nearly all EBV-positive NPC cases encode LMP1, a clinical trial employed the adenovirus-DeltaLMP1-LMP2 gene to transduce dendritic cells in patients with advanced NPC, yielding promising results [399]. Several studies have also suggested the safety and efficacy of EBV-specific cytotoxic T cell infusion, which targets EBV antigens including EBNA1, LMP1 and LMP2 (NCT01447056, NCT02065362) [400]. On the other hand, another therapeutic strategy, active immunotherapy, which utilizes tumor vaccines to enhance immune recognition, has also been explored in NPC treatment. These strategies encompass LMP2-expressing dendritic cells (DCs) and the use of a recombinant modified vaccinia Ankara vaccine (MVA-EL). Both approaches have demonstrated safety and good tolerability. Evidence from studies supported the efficacy of directly administering the MVA-EL vaccine in inducing LMP/EBNA1-specific CTL responses in patients [401, 402]. This recombinant vaccinia virus-based vaccine, encoding a functionally inactive fusion protein comprising the CD4 epitope-rich C-terminal half of EBNA1 and the CD8 epitope-rich LMP2A, demonstrated the ability to induce a T-cell response in 80% of patients [403]. In certain cases, it even enhanced responses involving both CD4^+^ and CD8^+^ mediated immunity against EBNA1 and/or LMP2. This vaccine is currently undergoing evaluation in a phase II trial that includes patients with detectable plasma EBV DNA levels after radiotherapy or those who have achieved an optimal response to palliative chemotherapy (NCT01094405). Besides that, LMP2 expressing DCs was also assessed in a phase II clinical trial with an adenovirus-DeltaLMP1-LMP2 vector [399]. However, there was no improvement observed in the levels of LMP1/2-specific T cells [399]. Immunotherapy alone may have limited effects on certain cancers. Therefore, combining therapeutic strategies may be necessary to enhance the therapeutic response.

The combination of PD-1/PD-L1 blockade with other immune checkpoint inhibitors, like anti-CTLA-4 or anti-angiogenic agents such as anti-VEGFR inhibitors, represents an enhanced therapeutic strategies for NPC. The efficacy of the combination of ipilimumab and nivolumab in NPC has been investigate in phase II clinical trial (NCT03097939) [404]. Preliminary data showed a partial response rate of 35% with a median response duration of 5.9 months, demonstrating a synergistic enhancement in the efficacy of PD-1 monotherapy. There is an ongoing phase I/II clinical trial (NCT02460224) evaluating the safety and efficacy of an anti LAG3 antibody, ieramilimab, as a standalone treatment and in combination with spartalizumab, an anti-PD1 antibody, for patients with advanced malignancies [405]. Ieramilimab demonstrated good tolerability when administered as a monotherapy and in combination with spartalizumab. The toxicity profile of ieramilimab in combination with spartalizumab was found similar to that of spartalizumab alone [405]. Additionally, combining PD-L1/PD-1 inhibitors with chemotherapy or radiotherapy has also been proposed as an alternative approach to enhance treatment outcomes. The combination of chemoradiation and PD-1/PD-L1 inhibitors has shown the potential to promote synergistic anti-tumor immunity through enhancing immune recognition, cytotoxic activity, and inhibiting T-cell apoptosis. Recent studies have reported the approval of camrelizumab or toripalimab combined with gemcitabine plus cisplatin regimen as first-line setting for recurrent and metastatic NPC treatment [244, 406]. For instance, a phase I trial on anti-PD1 antibody, camrelizumab achieved an ORR of 34% [244, 406]. In contrast, a combination of camrelizumab with gemcitabine and cisplatin achieved an impressive ORR of 91% (CAPTAIN study) [244, 406]. Furthermore, the addition of toripalimab to gemcitabine plus cisplatin chemotherapy for recurrent and metastatic NPC patients demonstrated impressive results, with a median PFS of 11.7 months (JUPITER-02 study) [407]. More recently, a phase III clinical trial (NCT03924986) demonstrated that the combination of tislelizumab with gemcitabine and cisplatin significantly extended the median PFS of recurrent or metastatic NPC patients. This improvement was notable, with a median PFS of 13.9 months, following a follow-up period of 15.5 months, when compared to placebo plus gemcitabine and cisplatin. Apart from this, combining PD-1 antibodies with chemotherapy to co-targeting the PD-1 axis with anti-VEGFR represents the third most frequently used clinical strategy in oncology treatment. There are ongoing clinical trials propose the combination of PD-1/PD-L1 blockade with other immune checkpoint inhibitors such as anti-CTLA-4 or anti-angiogenic agents such as anti-VEGFR inhibitors as recommended therapeutic approach for NPC [408, 409]. In a phase II clinical trial (NCT04586088), the combination of apatinib and camrelizumab has demonstrated promising antitumor activity in patients with refractory, recurrent, or metastatic NPC who had previously failed first-line therapy [410]. The trial reported an ORR of 65.50%, a DCR of 86.20% and a median PFS of 10.4 months [410]. Another combinational strategy involves the combination of cytotoxic T-cell (CTL) adoptive therapy with chemotherapy. In a phase II study, the application of a combination of chemotherapy with engineered EBV-specific CTLs (adoptive immunotherapy) as a first-line treatment for metastatic or recurrent NPC patients resulted in a favorable outcome, achieving a 2-year Overall Survival (OS) rate of 62.9% [40]. In an ongoing phase III trial (NCT02578641), a treatment regimen consisting of chemotherapy (gemcitabine and carboplatin) followed by autologous, in vitro-expanded EBV-specific cytotoxic T cells for recurrent and metastatic NPC patients treatment has yielded promising efficacy with an overall response rate of 71.4% [40]. Similar studies are underway, exploring adoptive cellular-based immunotherapies using EBV-T-cell receptor-T cell therapy (NCT03648697) and LMP-, BARF1-, and EBNA1-specific CTLs (NCT02287311).

Future studies focusing on exploring the crosstalk between various immune evasion mechanisms and their collective influence on the immune landscape within the TME could pave the way for more comprehensive therapeutic strategies. The heterogeneity of NPC tumor cell population within TME has enabled it to display diverse phenotypes for cancer progression. The interaction among these cells in TME is through secretion of cytokines, chemokines, membrane contact, and exosomes, in turn aberrantly regulating the biological process and immune evasion [411]. Therefore, uncovering the aberrantly regulated molecules in TME could help to explore the potential NPC therapeutic to block the immunosuppressive and tumor growth promoting effect. Furthermore, in order to achieve personalized NPC therapeutic, more international collaboration studies of oncogenic genetic and epigenetic alteration occurrence in NPC are required to further investigate the different subset of NPC patients.

## Recent clinical advancement of precision medicine in NPC

As we have comprehensively described the currently practiced treatment modalities and highlighted the limitations therein such as toxicities, therapeutic resistance, and limited applicability due to high cost, the unique physiological location of tumor, and the lack of efficacy in certain patients, more effort is warranted to pursue new therapeutic strategy for NPC patients. Knowing how tumor heterogeneity contributes to variable treatment outcomes in patients, precision medicine becomes the terminal avenue for seeking full remission. Below, we will summarize the recent clinical advancement in developing treatment strategies to meet a true precision medicinal practice that addresses the limitations listed above.

### Cytotoxic chemotherapy

Cytotoxic drugs have been the integral part of therapies for NPC patients due to their efficacies. However, the side effects commonly experienced by the recipients of such treatment options are the downsides that warrant a search for newer cytotoxic chemotherapy that is safe and efficacious.

A phase II clinical trial performing prospective randomized trial to compare the efficacy between CCRT plus S-1 (oral fluoropyrimidine) treatment regimen and CCRT plus cisplatin control regimen in patients with locoregionally advanced NPC [412]. Based upon Karnofsky performance status (KPS) ≥ 60%, adequate organ function, and null systemic metastasis, 105 stage III–IV (M0) NPC patients with no prior cancer history were assigned randomly into groups receiving chemoradiotherapy with 400 mg oral S-1 (twice/day, 7 days/week for 4 weeks) or chemoradiotherapy with 40 mg/m^2^ cisplatin (weekly for 7 weeks). Radiotherapy was given at 66–76 Gy/7–8 weeks to the primary tumor, 60–66 Gy/6–7 weeks to the positive neck region, and 50–55 Gy/5–6 weeks to the negative neck region. The study with a median follow up of 28.4 months (interquartile range (IQR), 9–50 months) found that the two regimens (CCRT plus S-1 vs. CCRT plus cisplatin) were not different significantly in terms of their efficacy measures measuring complete response (67.3% vs. 54%, respectively, *P* = 0.235), partial response (23.6% vs. 26%, respectively, *P* = 0.779), 2-year PFS (81.3% vs. 65.8%, respectively, *P* = 0.090), and OS 2-year OS (86.2% vs. 82.5%, respectively, *P* = 0.103). However, the treatment regimen consisting of CCRT plus S-1 was able to tone down some grade 3–4 toxicities, such as leukopenia (5.5% vs. 22%, respectively, *P* = 0.013), mucositis (20% vs. 46%, respectively, *P* = 0.004), dermatitis (14.5% vs. 36%, respectively, *P* = 0.011), and nausea (9.1% vs. 40%, respectively, *P* = 0.000) when compared to control regimen. This suggests that by replacing cisplatin with S-1 in current standard treatment regimen, toxicities may potentially be minimized when treating locoregionally advanced NPC patients. A larger trial with longer follow-up should be established to support the findings.

Another oral fluoropyrimidine cytotoxic drug called capecitabine was also assessed for its efficacy as adjuvant by combining with cisplatin compared to the efficacy when cisplatin was used alone [413]. Chemo- and radiotherapy-naïve patients aged ≥ 18 years old with locally advanced (stage III–IVa) NPC, and no other tumor were enrolled. A total of 136 patients with KPS ≥ 70%; adequate organ function; complete medical history and physical examination; complete hematologic and biochemical analyses; and complete imageological examination like MRI, CT, or PET–CT were assigned into two groups consisting of 66 patients in group A and 70 patients in group B. Three cycles of treatment were given consisting of 1 week rest after 2 weeks of treatment in each cycle. In the group of patients receiving intravenous drip of 20 mg/m^2^ cisplatin alone (group A, 5 days/week), the effective rate was 60.6% with 21 patients (31.8%) having complete response, 19 patients (28.8%) having partial response, 12 patients (18.2%) having stable disease, and 14 patients (21.2%) having progressive disease. In the group receiving 1000 mg/m^2^ adjuvant drug capecitabine in addition to group A’s cisplatin dosing strategy (group B, twice/day), the effective rate was 81.4% with 34 patients (48.6%) having complete response, 23 patients (32.9%) having partial response, 7 patients (10%) having stable disease, and 6 patients (8.6%) having progressive disease. The 3-year OS for the two groups of patients were 77.3% vs. 85.7% (*P* = 0.039), respectively. Lower recurrence and metastasis rates were observed in group of patients treated with capecitabine and cisplatin compared to patients treated with cisplatin alone (7.1% vs. 19.7, *P* = 0.030, respectively and 10% vs. 22.7%, *P* = 0.044, respectively). Interestingly, the total patients experiencing toxicities were fewer in the patients treated with cisplatin and capecitabine than in the patients treated with cisplatin alone (10% vs. 24.2%, respectively, *P* = 0.026). This may suggest that adding capecitabine into cisplatin treatment regimen may reduce toxicities in NPC patients. The benefit of adding capecitabine as an adjuvant still need further confirmation such as through randomized controlled trials that recruit larger sample size and follow-up for longer term.

Although S-1 and capecitabine appear to have more favorable toxicity profiles when compared to regimen that includes chemoradiotherapy or chemotherapy alone, respectively, other notable adverse effect such as cardiotoxicities including events like chest pain, coronary syndrom/infarction, arrhythmia, heart failure/cardiomyopathy, cardiac arrest, and malignant hypertension might be a problem, despite occurring at a much lower frequency and of lower grade of adverse event when using S-1 [414]. Hand–foot syndrome is also a common side-effect of S-1 and capecitabine treatments, and again, it is less likely to occur and of lower severity in S-1 treatments [415]. It would be insightful to study the molecular mechanisms of S-1 and capecitabine’s apparent reduced toxicity when used together with other treatment modalities in order to figure out how to best apply these two drugs and their potential synergies with other available therapies such as targeted therapy and immunotherapy. Furthemore, utilizing a technology like RNA-sequencing to obtain gene expression profiles of best responding subjects before and after treatment would unravel potential biomarkers that can predict treatment response, hence helping in guiding biomarkers-based personalized treatment. Overall, trial recruiting larger patient sample size with diverse patient population and longer follow-up should be established to firmly decide on whether S-1 should replace cisplatin and capecitabine can be added into cisplatin treatment regimen for safer NPC management.

### Targeted therapy

With the increasing understandings of the aberrations in genetics, epigenetics, and signalling pathways in NPC tumors, the need to develop targeted therapy to inhibit potential drivers for CSCs transformation becomes apparent. The testing of such small molecule or antibody-based inhibitors has already been reported in several clinical trials summarized below.

An AKT inhibitor, MK-2206, was tested in multicenter phase II clinical trial enrolling patients with local and/or distant sites metastatic and/or recurrent non-keratinizing NPC [127]. Patients were selected to have disease progression within 24 months of receiving one or two prior lines of chemotherapy for recurrent disease with at least one of the regimens containing platinum agents. The patients should also have Eastern Cooperative Oncology Group (ECOG) performance status of 0 to 2, adequate organ, and bone marrow function. MK-2206 was given at 200 mg once a week on days 1, 8, 15, and 22 in a 28-day interval until disease progression, development of intolerable toxicity, or refusal by patient. Out of 21 patients analyzed, one patient (5%) had partial response and 11 patients (52%) had stable disease. The median PFS was 3.5 months (IQR, 0.9–7.3 months) and the 6-month PFS rate was 43%. On the other hand, the median OS was 10.0 months (IQR, 5.9–20.0 months) and 6-month OS rate was 70%. Nine patients (42.9%) were progression-free at 6 months and two patients (10%) were progression-free beyond 12 months. Some grade 3 toxicities were observed including macular–papular rash, hyperglycemia, and fatigue. Although the study was terminated prematurely due to limited activity observed, the study observed a benefit from inhibiting AKT for a patient with NPC possessing *PIK3CA* gene amplification as the patient with the aforementioned amplification in primary tumor was observed to maintain stable disease condition over 12 months period. The author emphasized the need to developed predictive biomarkers of response to inhibitors of AKT and the need to use combinatorial approach to tackle the activation of compensatory pathways in NPC. Testing of MK-2206 in a specific population of patients harboring *PIK3CA* gene amplification should be performed in larger and longer term randomized trials to demonstrate its clinical efficacy in this particular subgroup of patients.

In an attempt to address amplified *EGFR* gene and overexpressed EGFR protein of in NPC, tyrosine kinase inhibitors (TKIs) are also used to treat NPC patients. A phase I clinical trial employing a novel highly selective EGFR-targeting TKI drug icotinib attempted to study the combination of the EGFR TKI drug with IMRT in NPC patients [416]. NPC patients aged 18–70 years old without distant metastasis with KPS ≥ 70; adequate renal, hepatic, and bone marrow function were recruited. Oral icotinib was given until the completion of radiotherapy; IMRT was implemented 3–6 h after icotinib treatment. The dose-escalation study was started at 125 mg/day, escalated by increasing the frequency of treatment (one, twice, to thrice daily). If less than two patients suffered dose-limiting toxicity, i.e., grade 4 mucositis and skin toxicity, grade 3 mucositis with delay of more than 1 week due to toxicity treatment, or any other grade 3 non-hematologic toxicity (except nausea and vomiting) that was considered dose-limiting, the escalation continued, otherwise the escalation was stopped. At least 4 weeks of observation was required after IMRT within each icotinib dose level before moving onto the next level. The study concluded a dose at 125 mg/day of icotinib to have an acceptable safety profile and was well-tolerated when combined with IMRT in NPC patients, whereas the dose at 250 mg/day induced mucositis as the dose-limiting toxicity in half of the enrolled patients in that particular cohort. Patients with *EGFR* mutation (four patients) appeared to suffer more serious adverse effects than the patient without. Further study needs to investigate whether there is an association between the presence of mutation of *EGFR* in exon 18 and 20 with the adverse event. Better biomarkers than EGFR are also needed to better predict the response of TKI treatment in this case. Since this was a phase I study, phase II trial recruiting more patients with longer term follow-up is necessary to further demonstrate its safety and potential efficacy.

The EGFR-targeting TKI drug cetuximab had also been evaluated for efficacy and toxicity in several studies. In a multicenter phase II clinical study, cetuximab was assessed in combination with carboplatin in patients with recurrent or metastatic NPC who had disease progression on or within 12 months after the end of platinum-based chemotherapy for their recurrent or metastatic diseases [417]. More specifically, the patients had to have EGFR expression in the NPC tumor, aged ≥ 18 years old, KPS ≥ 60, and adequate organ function. Cetuximab was given at 400 mg/m^2^ over 120 min initially, and then continued with weekly dose of 250 mg/m^2^ over 60 min. Carboplatin with a targeted area under the curve (AUC) of 5 (according to the Calvert formula) was administered after the cetuximab infusion on day 1 of a 21-day treatment cycle. The combination was given till a maximum of eight cycles or progression disease or patients suffered unacceptable toxicity. Cetuximab dose was modified to 200 and then 150 mg/m^2^, respectively if the treatment was interrupted for up to two consecutive infusions on the second and third occurrence due to grade 3 skin toxicity. Cetuximab was discontinued on the fourth of such an occurrence. Fifty-nine patients’ response was assessable out of the 60 enrolled patients. Seven patients (11.7%) had partial response, 29 patients (48.3%) had stable disease, whereas 23 patients (38.3%) suffered progressive disease. The median time to progression was 81 days and median OS time was 233 days in all patients. Six patients (10%) suffered serious treatment-related adverse events and 31 patients (51.7%) suffered grade 3/4 toxicities, from which 19 patients (31.7%) were claimed to have toxicity related to cetuximab. Since the patients were heavily pretreated, the occurrence of many adverse events was expected. It was clear that the combination was tolerable as there were no dose modifications necessary. Future trials should aim to enroll more patients and with longer follow-up time.

Cetuximab was also evaluated when used in combination with CCRT in locoregionally advanced NPC patients [418]. The analyses included 225 patients from the enrolled 681 newly-diagnosed locoregionally advanced (stage III–IVB) non-keratinizing NPC patients who received CCRT with (75 patients) or without (150 patients) cetuximab. IMRT was given with the prescribed doses of 66–75 Gy at 2.10–2.25 Gy/fraction to the planning target volume (PTV) of the primary gross tumor volume, 64–72 Gy per 28–33 fractions to the PTV of the involved lymph nodes volume, 60–62 Gy per 28–31 fractions to the PTV of the high-risk clinical target volume, and 50–52 Gy per 25–30 fractions to the PTV of the low-risk clinical target volume. The group with the addition of cetuximab received concurrent chemotherapy regimen consisting of paclitaxel and nedaplatin, whereas the concurrent chemotherapy regimen for the group without the addition of cetuximab could vary between docetaxel (70 mg/m^2^ on day 1) with cisplatin (60 mg/m^2^ on day 1–3), or 3-weekly cisplatin (80 mg/m^2^ on day 1–3)/nedaplatin (80 mg/m^2^ on day 2–4). Cetuximab was initially given at 400 mg/m^2^ by intravenous infusion over 120 min and subsequently 250 mg/m^2^ over 60 min on a weekly basis. The median follow-up time was 43.6 months (IQR, 5.6–75.3 months) for CCRT only group, wheras the median follow-up time was 41.0 months (IQR, 6.0–71.2 months) for CCRT plus cetuximab group. Statistically, significant improvement in 3-year PFS (83.7% vs. 71.9%, respectively, *P* = 0.036), locoregional failure free-survival (LRFS) (98.6% vs. 90.2%, respectively, *P* = 0.034) but not OS (91.4% vs. 85.4%, respectively, *P* = 0.117) was observed in group of patients treated with CCRT combined with cetuximab compared to CCRT alone. Specific subgroup of patients with T4 and/or N3 category was seen to benefit from combination of cetuximab with CCRT with significant prolonged 3-year PFS (81.0% vs. 61.4%, respectively, *P* = 0.022) and longer 3-year OS (88.0% vs. 77.9%, respectively, *P* = 0.086), whereas there were no significant differences observed for PFS, LRFS, OS, and distant metastasis-free survival (DMFS) rates in general for stage III patients. Adverse events such as acute oral and oropharyngeal mucositis were more frequently seen in group with cetuximab addition, although late toxicities were similar in both arms. There were several limitations highlighted by the authors of the present study. The fact that data were obtained from patients of a single institution limited the generalizability of the results. There was limited toxicity statistics obtained in the relatively short period of follow-up to better justify the proposed combinatorial treatment and there was potential bias in choosing the patients data being presented in this study. Hence, future multicenter prospective studies with longer period of follow-up should be conducted to better generalize the results from this study.

Recently, the long-term survival of patients with chemotherapy-naïve metastatic NPC was assessed with the inclusion of cetuximab in docetaxel and cisplatin treatment regimen [419]. Patients aged 18–65 years old with newly diagnosed metastatic NPC and patients with first metastatic NPC relapse after radiotherapy were eligible for the study. Patients had to have ECOG performance status of 0 or 1; had not received previous treatment with any investigational drug, surgery, irradiation or other anticancer therapies within the prior 4 weeks; had no known central nervous system metastases; had adequate organ function; had no uncontrolled cardiac or other disease with life expectancy of 3 months to be eligible. All patients had EGFR^+^ NPC. Induction chemotherapy consisting of 75 mg/m^2^ intravenous docetaxel on day 1, 25 mg/m^2^ cisplatin on days 1, 2, and 3, and 250 mg/m^2^ cetuximab on days 0, 7, and 14 with initial dose of 400 mg/m^2^ was given to all patients, followed by CCRT consisting of IMRT plus concomitant 75 mg/m^2^ cisplatin (every 3 weeks for two cycles) and cetuximab (every week for six cycles) for patients with objective remission, and followed by maintenance therapy consisting of 1000 mg/m^2^ capecitabine twice daily on days 1 through 14 (every 21 days). IMRT was given at 68–70 Gy (30 daily fractions over 6 weeks) to the PTV of existing primary tumor in 17 patients with newly diagnosed metastatic disease and at 64–66 Gy in 26 patients with first relapse-metastases after curative radiotherapy. The latter group was given additional dose of 62–66 Gy over 30 fractions to metastatic regional neck node if indicated. Induction therapy was given for a maximum of six cycles, until disease progression, development of intolerable toxicity, or refusal by patient. Patients receiving conventional therapy, i.e., CCRT served as control group. The median follow-up time was 89 months (IQR, 32–135 months). The overall median OS and PFS time were 32.9 months (IQR, 18.2–47.5 months) and 18.3 months (IQR, 10.6–26.0 months), respectively. The 5-year OS and PFS rates for all patients were 34.9% and 30%, respectively. Patients with newly diagnosed metastatic NPC seemed to benefit from the regimen more than those who previously received radiotherapy and then experienced relapse with metastases with 5-year OS rate of 58.8% vs. 19.2% and 5-year PFS rate of 52.9% vs. 19.2%, respectively. These patients when compared to control patients (5-year OS, 10.9%) had significantly more long-term survivors. Importantly, there were only few patients sufferring grade 3 skin reactions with no treatment-related mortalities in the present study. The most common grade 3/4 side effects of the induction therapy were leucopenia (39.5%), acne-like rash (11.6%), febrile neutropenia (14%), and thrombocytopenia (9.3%). About 10% of the patients were susceptible to grade 3/4 toxicities during CCRT, including oral mucositis (39.1%), dermatitis (in-field) (26.1%), leukopenia (17.4%), acne-like rash (13%), and thrombocytopenia (13%). Grade 3/4 adverse events were rare during maintenance therapy. Due to its safety, cost effectiveness, and convenience, capecitabine was selected as the maintenance therapy. As a result, one-third of patients experienced progression disease. Hence, the role of targeted therapy such as cetuximab or even immunotherapy can be assessed in this setting. Nevertheless, the regimen assessed in this study conferred long-term survival for 15 patients (34.9%), 12 of which were still alive with no evidence of disease at 60–135 months of follow-up. Future studies should focus on recruiting more patients with diverse background to confirm the versatility of the regimen to wider population.

To summarize, cetuximab may be beneficial for a specific subgroup of patients such as those with T4 and/or N3 category and those who never receive chemotherapy and radiotherapy after diagnosis.

An alternative TKI drug for targeting EGFR called nimotuzumab was also explored to treat NPC patients. In a retrospective study, 42 locally advanced NPC patients who receiving nimotuzumab combined with CCRT were studied and analyzed [35]. Four to six cycles of cisplatin-based concurrent chemotherapy was received by all patients; 27 received concurrent chemotherapy only, 7 received induction plus concurrent chemotherapy, and 8 received induction, concurrent plus adjuvant chemotherapy. All patients also were administered with nimotuzumab for four to seven cycles; 13 patients received 100 mg/week and 29 patients received 200 mg/week. The study reported that patients who received the aforementioned regimen had complete response and partial response rate of 90.5% and 9.5%, respectively. The 2-year LRFS, DMFS, and OS rates were 96.4%, 93.1%, and 96.6%, respectively. At the last follow-up, four patients (9.5%) were reported to have progressive disease and two patients (4.8%) died of distant metastasis. Mucositis and hematology toxicities were the most common adverse events and some grade 3/4 toxicities in few cases. From the report, it was not clear how the patients were assigned to each group of CCRT treatment, what were the constituent of the CCRT regimen, and what doses were used for respective constituent. Furthermore, the data were generated from a short follow-up and small sample size. Further randomized trial with larger sample size and longer follow-up is required to confirm the conclusion of the study.

Nimotuzumab was recently evaluated for its best mode of application whether it is best applied along with induction chemotherapy or along with CCRT in a regimen that includes induction chemotherapy and chemoradiotherapy [420]. The enrollment criteria were: age 17–73 years old; KPS ≥ 70; adequate organ function; no known allergies; no history of mental illness; no drug abuse or other unhealthy habits; no metastasis or malignancy in other organ; no history of other malignancy, participation in other clinical trials, severe allergies, pregnancies, lactations, treatment of anti-EGFR; and tolerance to therapy. One hundred eighty patients were categorized into group A consisting of 120 patients receiving induction chemotherapy followed by CCRT, group B consisting of 30 patients receiving nimotuzumab at the beginning of induction chemotherapy followed by CCRT, and group C consisting of 30 patients receiving induction chemotherapy followed by nimotuzumab applied at the beginning of radiotherapy plus CCRT. Induction chemotherapy regimens were given for two to three cycles consisting of docetaxel plus cisplatin plus 5-FU, docetaxel plus cisplatin, or cisplatin plus 5-FU. Docetaxel was at 70 mg/m^2^ on day 1 over 1 h, cisplatin was at 75 mg/m^2^ on day 1–3, and 5-FU was at 500 mg/m^2^ from day 1–4 as an intravenous infusion every 3 weeks. Concurrent chemotherapy was given for one to three cycles with 80 mg/m^2^ cisplatin that was evenly distributed across 3 days (every 3 weeks). Nimotuzumab was given weekly at 200 mg in 250 mL of intravenous infusion within physiological saline administered over 60 min. IMRT (66–69.75 Gy) or three-dimensional conformal radiotherapy (68–73 Gy) was used for radiotherapy of gross tumor volume. For lymph node, 60–70 Gy was delivered, whereas for clinical tumor volume, 50–62 Gy was delivered. In this prospective non-randomized study with 168 assessable advanced NPC patient data, the median follow-up time was 61.4 months (IQR, 17–96.5 months). The, addition of nimotuzumab regardless of the mode of application resulted to significant improvement of 5-year OS (87.0 ± 4.6%) vs. without nimotuzumab (74.8 ± 4.1%, *P* = 0.043) but not 5-year PFS (83.1 ± 5.1% vs. 72.7 ± 4.3%, respectively, *P* = 0.243). In the course of follow-up, disease progression occurred in 34 and 13 patients receiving regimen without and with nimotuzumab, respectively, and the median time to progression was 26.6 and 45.3 months, respectively. When considering the mode of application, adding nimotuzumab during the induction chemotherapy stage had higher benefit for 5-year OS (93.0 ± 4.8%) vs. without nimotuzumab (74.8 ± 4.1%, *P* = 0.038) but not for 5-year PFS (89.3 ± 5.9% vs. 72.7 ± 4.3%, respectively, *P* = 0.144). No significant improvement was observed when comparing the addition of nimotuzumab during chemoradiotherapy stage and without nimotuzumab for both OS (80.4 ± 7.9% vs. 74.8 ± 4.1%, respectively, *P* = 0.257) and PFS (76.4 ± 8.5% vs. 72.7 ± 4.3%, respectively, *P* = 0.611). Notably, there was no grade 3/4 toxicities in induction chemotherapy setting. In concurrent chemotherapy setting, nausea and vomiting were common in patients receiving cisplatin. Other common side effects were oral mucositis, leukocytopenia, and skin reaction. Specific toxicity induced by the addition of nimotuzumab was a rare find. Xerostomia was the most common late adverse effect but was self-limiting. To avoid bias, future clinical trials should randomize the patients in the assignment of regimens.

Despite the improvement offered, a phase III randomized trial concluded that nimotuzumab (200 mg/week for 8 weeks) did not differ much in OS (93.5% vs. 94.8%, respectively, *P* = 0.95) and PFS (79.8% vs. 83.5%, respectively, *P* = 0.69) rate when compared to cisplatin (40 mg/m^2^/week for 5 weeks) in a combinatory regimen with radiotherapy (70 Gy, 35 fractions) after initial induction chemotherapy consisting of docetaxel, cisplatin, and 5-FU in stage III–IVB locally-advanced NPC patients [421]. However, the occurrence of grade 3/4 gastrointenstinal toxicities were significantly higher in cisplatin group compared to nimotuzumab group (33.7% vs. 4.2%, respectively, *P* < 0.001). The instance for grade 2/4 hematologic reactions was also higher in cisplatin-treated patients than in nimotuzumab-treated patients (59% vs. 9.7%, respectively, *P* < 0.001). Nonetheless, there was no difference between the two groups for the occurrence of grade 3/4 mucositis and dermatitis (41% vs. 27.8%, respectively, *P* = 0.13).

In conclusion, nimotuzumab may be given during the induction chemotherapy stage to be advantageous from the improvement of OS for advanced NPC patients.

A multikinase inhibitor called famitinib which targets stem cell growth factor receptor Kit (KIT), platelet-derived growth factor receptor (PDGFR), and VEGFR was tested in patients with recurrent and/or metastatic NPC after failing more than two previous treatment regimens [37]. Eligible patients should have ECOG performance status ≤ 2, adequate organ function, and no prior exposure to other c-Kit, PDGFR or VEGFR TKIs. The single-arm study revealed that a daily dose of 25 mg of famitinib was able to confer clinical benefit rate (CBR) of 32.8% that was maintained throughout ≥ 12 weeks period with five and 16 patients demonstrated partial response and stable disease, respectively. The median PFS was 3.2 months. Adverse events were generally mild-to-moderate and manageable, whereas the occurrence of grade 3/4 toxicity was rare. They included hematologic toxicities such as thrombocytopenia, leucopenia, and neutropenia; non-hematologic adverse events were hypertension, proteinuria, and hand-foot syndrome. The next phase trials should further demonstrate this clinical finding in trials recruiting more patients with longer follow-up.

A phase I non-randomized dose-escalation study then assessed famitinib combination with CCRT in 20 treatment-naïve patients with locoregionally advanced NPC(M0) [422]. Eligible patients had to have ECOG performance status of 0 or 1; aged 18–65 years old; have adequate renal, hepatic, and bone marrow function; have no history of or the presence other malignancy; have no history of spinal cord compression or diseases of the brain; have no hypertension, myocardial ischemia, arrhythmia, or cardiac insufficiency; have no long-term untreated wounds or fractures; no preexsisting thyroid dysfunction; have no history of psychiatric drug abuse or dysphrenia; and be not subject of hepatitis B/C or human immunodeficiency virus (HIV) infection.The oral famitinib was given in two courses consisting of neoadjuvant monotherapy for 2 weeks prior to chemoradiotherapy and then given in combination with chemoradiotherapy for 7 weeks. If dose-limiting toxicities were developed in one of three patients, three additional patients were added and escalation continued; if two of six patients faced dose-limiting toxicities, the dose was defined as above the maximum tolerable dose. The dose was started at 12.5 mg/day, then increased to 16.5, 20, and to a maximum 25 mg/day. IMRT was used at 70 Gy (33 fractions) for PTV of gross tumor volume, 60 Gy (33 fractions) for PTV of high risk clinical target volume, 54 Gy (33 fractions) for PTV of low risk clinical target volume, and 66–68 Gy for PTV of nodal gross tumor volume. Cisplatin was given at 100 mg/m^2^ on days 1, 22, and 43 of radiotherapy. If two of three patients had a dose-limiting toxicity at the intial dose of famitinib, the concurrent cisplatin dose was reduced to 80 mg/m^2^ for remaining patients. Some dose-limiting toxicities were noted including grade 3 toxicity hypertension and grade 4 toxicity thrombocytopenia in 25 mg/day cohort. Partial response was already seen at the famitinib monotherapy stage at 15%, whereas the complete response was shown at 65% and 95% after completion of treatment and 3 months after completion of treatment, respectively. The median follow-up time was 44 months and during the 3 years follow-up, five patients were found to suffer distant organ metastasis. All five patients received palliative care, two of whom died of the disease. The 3-year PFS and DMFS were 70% and 75%, respectively. This encouraging result suggested a 20 mg of famitinib in combination with CCRT (80 mg/m^2^ cisplatin) to be given to locoregionally advanced NPC patients for future phase II clinical trial. Potential limitations for the study was that the conclusion was derived from low number of patients in a non-randomized open-label study that also used a relatively new tool called contrast-enhanced ultrasound to predict early treatment response. Future study should explore the efficacy of famitinib in larger sample size of NPC patients with longer term of follow-up.

Yet another TKI drug targeting VEGFR called axitinib was evaluated in patients with recurrent or metastatic NPC in a phase II clinical trial reported by Hui et al. [423]. The selection criteria included patients having ECOG performance status 0 or 1; aged ≥ 18; adequate bone marrow, renal and hepatic reserve; disease progression following at least one line of prior platinum-based chemotherapy; no presence of local recurrence; no presence of neck lymph node or central lung lesions invading vascular structure; no history of hemoptysis or epistaxis within 4 weeks; and no preexisting uncontrolled hypertension. Axitinib was taken orally with food starting at 5 mg twice daily for 4 weeks. Tolerance towards axitinib throughout the 4 weeks treatment with no grade > 2 adverse event warranted dose escalation up to two doses (7 and 10 mg). For patients experiencing grade 3/4 adverse effect, dose reduction to 3 and 2 mg was allowed. Out of 40 enrolled patients who received a median of three lines of prior chemotherapy, 37 patients had evaluable response with CBR of 78.4% after 3 months and 43.2% after 6 months. The 1-year survival rate was 46.3%. The median OS and time to progression was 10.4 months (IQR, 6.8–19.0 months) and 5.0 months (IQR, 3.9–5.7 months), respectively. Less than 10% of the patients experienced grade 3/4 toxicities including diarrhea (5%), hypertension (8%), pain (5%), and weight loss (5%). Hemorrhagic events occurred during treatments were either grade 1 (15%) or grade 2 (3%). Other common adverse events (grade 1 or above) included hand–foot syndrome (50%), hypothyroidism (50%), and fatigue (40%). The down side of using axitinib appeared to be its effect in inducing hypothyroidism which was the second most common adverse events regardless of grade. Fatigue symptom appeared to be related with hypothyroidism as the onset of the latter was significantly associated with the former (*P* = 0.039, Fisher exact test). Thyroid function returned to normal after ceasing treatment of axitinib, indicating hypothyroidism to be temporary side-effect. Due to the immunomodulatory effect of VEGFR inhibitors and the favorable toxicity profiles of axitinib, the authors suggested combinatorial approach to enhance the depth and durability of response in future trials.

An anti-angiogenic agent called endostar (recombinant human endostatin) was also used to treat NPC patients. Patients with locally recurrent III–IVB NPC were selected based on the criteria such as age ≥ 18; KPS ≥ 70; adequate bone marrow, hepatic, and renal function; no evidence of distant metastasis; at least 6 months after the end of initial course of radiotherapy; and had received Endostar combined with radiotherapy/chemotherapy [424]. In the retrospective study, 22 patients with relapsed NPC underwent treatment with neoadjuvant platinum-based chemotherapy and salvage radiotherapy combined with endostar. All patients received IMRT with median prescribed dose of 64 Gy (60–68 Gy)/32 fractions (28–33 fractions) and median fractionated prescribed dose of 2.10 Gy (1.88–2.29 Gy). Among the 22 patients, 18 of them was administered with cisplatin-based neoadjuvant chemotherapy. On the other hand, endostar was administered at 105 mg/m^2^/day for 14 days in a 21-day cycle. With a median follow-up time of 13 months, 20 patients had complete response and 2 patients had partial response. The 1-year and 2-year survival statistics were OS: 93.3% vs. 66.4%, respectively; LRFS: 89.3% vs. 78.1%, respectively; DMFS: 90% vs. 78.8%, respectively; and PFS: 92.3% vs. 52.7%, respectively. Some grade 3–5 late adverse events included radiation injury (50%) and nasopharyngeal mucosal necrosis (31.8%). Other toxicities were cardiotoxicity (5%), mucositis (14%), and myelosuppression (14%). Four fatalities were encountered due to tumor metastases (two patients), nasopharyngeal hemorrhage (one patient), and radiation temporal lobe necrosis (one patient). Metastasis occurred in multi-sites in one case and liver in another. Despite the drawbacks, the authors urged for the conductance of prospective trials recruiting more patients and with longer follow-up term for confirmation of the encouraging efficacies.

One case study reporting three patients with refractory NPC receiving continuous infusion of endostar (15 mg/m^2^/day, 30-day cycle) combined with chemoradiotherapy consisting of intravenous drip of nedaplatin (80 mg/m^2^/day on days 1 and day 28) plus continuous low-dose intravenous infusion of 5-FU (200 mg/m^2^/day, 30-day cycle) plus concurrent low dose radiation (100 to 120 cGy, at first cycle) saw that the two-cycle regimen could decrease the load of EBV DNA in plasma and regress tumor; two of which had complete response and the other had partial response [425]. Efficacy should be replicated in trials enrolling larger sample size with longer follow-up duration.

Overall, the usage of targeted therapy is flexible; it can be administered along with induction chemotherapy, along with radiotherapy, along with chemoradiotherapy, or as a single agent. However, the above studies suggested that combinatorial approach demonstrated higher efficacies for patients with the correct mode of application. It is often able to reverse resistance (hence its usage), however it also tends to induce additional toxicities. Therefore, the effort to optimize dose, selection of targeted therapy, and selection of the right population of patients to be treated are crucial in order to minimize adverse effects during treatment. The targets of therapy, e.g., EGFR, did not seem to always guarantee efficacy. The study of icotinib found a worse scenario where *EGFR* mutation predicted for more serious adverse effects. Clearly, there is a need to find alternative biomarkers to more definitively predict response of targeted therapy. This can simply be done by assessing the molecular profiles of patients who perform exceptionally well to the targeted therapy. Applying multiple targeted therapies after finding out the potential resistance mechanisms and compensatory pathways might confer better clinical efficacy. Immunotherapy can also be a clear combinatorial candidate to harness the adaptive capability of the immune system.

### Radiotherapy

In response to the observed sensitivity of NPC to radiotherapy as well as the looking-to-improve toxicity imposed by widely used photon-based radiotherapy, i.e., IMRT, a better technique of radiotherapy is currently considered. The proton radiotherapy uses proton beam that is characterized by significant dose reduction after hitting the tumor as the initial target and thus leaving the normal tissue located behind the tumor that is along the beam path minimally afflicted [426].

Usage of proton radiotherapy has been evaluated clinically in several studies. A retrospective analysis of proton-reirradiation was conducted from the data of 17 patients with recurrent NPC [427]. Patients were recruited based on the criteria that they had no metastatic disease with ECOG performance status of ≤ 2 and the absence of severe comorbidities. The median dose of reirradiation using proton therapy was 60 Gy with a median reirradiation fractionation dose of 2 Gy. Most patients (53%) received proton therapy plus chemotherapy; one (5.9%) receiving induction chemotherapy, one (5.9%) receiving induction plus concomitant chemotherapy, three (17.6%) receiving concomitant cisplatin, and four (23.5%) receiving concomitant carboplatin. Most patients (64.7%) received IMRT before proton therapy reirradiation. The median follow-up time was 10 months (IQR, 2–41 months). The results suggested that the 18-month OS and local control rates were 54.4% and 66.6%, respectively. Of seven patients who died during the follow-up, four of them died of local cancer progression. A fatal bleeding case was identified with uncertain cause in one patient, which could be due to tumor recurrence or carotid blowout. Acute grade 2 mucositis only occurred in three patients (17.6%). Grade 2 soft tissue necrosis occurred as a late adverse effect in two patients (11.8%) whereasgrade 3 and 4 late event toxicities occurred in four patients (23.5%) with the most frequent event being hearing impairment (17.6%). Further comparative studies with larger sample size and longer follow-up are still necessary to confirm whether proton therapy is really more superior than IMRT in offering survival and toxicity benefits in this reirradiation setting.

Consistently, another cohort of 21 patients suffering locally advanced NPC had also evaluated the feasibility of proton therapy treatment as the replacement of the standard care [428]. The 16-month median follow-up revealed that OS, DMFS, and local control rates were 90%, 90% and 95%, respectively with only one patient suffering local failure, two patients having distant metastases, and some patients developing acute grade 3 toxicities including mucositis (66.7%) and dermatitis (42.9%). Late event toxicities included grade 2 xerostomia (10%) and hearing loss (14.3%). To clearly show the superiority of proton therapy over IMRT in treating locally advanced NPC patients, comparative study in larger trials with longer follow-up is necessary.

Another retrospective study was done by collecting data from 77 patients with newly diagnosed nonmetastatic NPC who underwent IMRT or intensity-modulated proton therapy (IMPT) [429]. The analysis included data from patients that fulfilled the criteria such as patients who were ≥ 18 years old, treated with chemoradiotherapy or radiotherapy alone with curative intent, and having follow-up data after the completion of treatment. To the gross tumor volume, high-risk anatomic sites, and low-risk anatomic sites, radiation doses were delivered at 69.96, 56–59.4, and 54.12 Gray equivalent (GyE), respectively, in 33 fractions or 70, 59–63, and 56 GyE, respectively, in 35 fractions. Cisplatin was administered as the concurrent chemotherapy at 40 mg/m^2^ weekly for seven cycles or 100 mg/m^2^ every 3 weeks for three cycles. From 77 patients, 7.8% received radiotherapy alone, whereas 92.2% received chemoradiotherapy. Twenty eight patients (36.4%) were treated with IMPT and 49 patients (63.6%) were treated with IMRT. The median follow-up time for the entire cohort, IMPT group, and IMRT group was 30.3 months (IQR, 17.9–41.5 months), 18.7 months (IQR, 13.5–30.0 months), and 37.0 months (IQR, 26.0–44.0 months), respectively. After accounting for the loss of follow-up for two patients in the IMPT group, the median follow-up time increased to 23.0 months (14.6–30.2 months). Improved toxicity outcomes were observed in IMPT when compared to IMRT based on multivariable logistic regression analysis with lower grade 2 or worse acute adverse events occurrence in IMPT group (67.9% vs. 93.9%, respectively, *P* = 0.01) Those specific acute adverse events included dysphagia, fatigue, xerostomia, dysgeusia, oral mucositis, weight loss, and hoarseness. Acute grade 3 adverse events occurred in three patients (10.7%) who received IMPT, whereas 11 patients (22.4%) who received IMRT suffered the same toxic effects which included dysphagia, oral mucositis, weight loss, and nausea. Chronic grade 3 adverse events occurred in one case (3.8%) of IMPT group, i.e., dysphagia, compared to eight cases (16.3%) of IMRT group, i.e., severe dysphagia, severe hearing impairment, severe weight loss, severe oral pain (multivariable logistic regression analysis, *P* = 0.11). Judging by the radiation delivery to organ at risk, IMPT was associated with significantly lower median of mean oral cavity dose (15.4 vs. 32.8 GyE, respectively, *P* < 0.001), lower median of mean larynx dose (16.0 vs. 29.6 GyE, respectively, *P* < 0.001), and lower median of mean parotid gland dose (22.5 vs. 25.2 GyE, respectively, *P* = 0.01) compared to IMRT. The2-year LRFS was 100% vs. 86.2%, *P* = 0.08; 2-year PFS was 95.7% vs. 76.7%, *P* = 0.14; and 3-year OS was 100% vs. 94.1%, *P* = 0.42 in IMPT group and IMRT group, respectively. Although there was not statistically significant difference between the two methods when looking at the survival statistics, IMPT clearly conferred better survival outcomes with huge benefit in reducing toxicities. Owing to the retrospective nature, low sample size, and imbalanced median follow-up time of the study, the conclusion limits the comparative strength between the two methods. Prospective studies with larger sample size and sufficient follow-up time should clearly justify the use of IMPT in the future.

These studies suggest that IMPT may be further employed to further improve the toxicity induced and clinical outcomes in IMRT-treated NPC patients, potentially replacing IMRT as standard care to minimize toxicity altogether. Because late toxicity imposed by radiotherapy is also a concern, long-term outcomes of IMPT should be pursued by observing its effects in larger patient cohorts to further balance its benefits and potential risks. Comparative studies comparing not only IMRT, but also other radiotherapy technique like stereotactic body radiation therapy (SBRT) could provide a better informed decision in the selection of the right technique for the right situation with tolerable toxicities and expected efficacies. Finally, with further exploration of optimal dose, patient selection criteria, and potential combinatorial regimen with targeted therapy and immunotherapy, proton therapy may shape a personalized treatment that is safe even for heavily treated NPC patients.

### Immunotherapy

The inherent ability of the human’s body immune system to prevent and eliminate tumorigenic growth can be exploited to achieve a more sustainable cancer-free remission through immunotherapy. Hitherto, immunotherapy has used several methods to induce a more active immune-dependent cancer clearance, including immune checkpoint inhibition, adoptive immunotherapy, and active immunotherapy [2].

#### Immune checkpoint inhibitor

Nivolumab, a recombinant, humanized monoclonal antibody designed to binds to PD1—an immune checkpoint molecule, had been studied for its therapeutic effect in a couple of trials enrolling NPC patients. In a multicenter trial studying nivolumab’s antitumor activity in 44 patients with recurrent and metastatic NPC were recruited based on the criteria that they had at least one prior line of platinum-based chemotherapy treatment for relapse, were untreatable for curative intent, and they had adequate organ function [385]. Intravenous 3 mg/kg nivolumab was administered every 2 weeks for 4-week cycle until disease progression. The median follow-up was 12.5 months (IQR, 2.2–22.0 months). The ORR was 20.5% with one patient (2.3%) having complete response, eight patients (18.2%) having partial response, 15 patients (34.1%) having stable disease, 18 patients (40.9%) having disease progression, and two patients (4.5%) were not assessed for response. The median OS and PFS were 17.1 months (IQR, 10.9 months–not reached) and 2.8 months (IQR, 1.8–7.4 months), respectively. The 1-year OS and PFS rates were 59% and 19.3%, respectively. Thirteen patients (29.5%) were still on the nivolumab treatment 6 months beyond enrollment and nine patients (20.5%) received it for > 12 months. Majority of patients (69.2%) dropped out of the trial due to disease progression and some cases (10.3%) due to adverse events. Grade 3 or higher adverse events were noted in around 22% of patients, including colitis, diarrhea, fatigue, increased aspartate transaminase (AST) or alanine transaminase (ALT) levels, neutropenia, hyponatremia, and lymphopenia. One patient died of pulmonary tuberculosis during treatment.

The correlation between the response of nivolumab and the presence of some biomarkers was also assessed in this study. Although there was not statistically significant association between ORR and biomarker’s levels (EBV DNA, PD-L1, HLA-A, and HLA-B), the descriptive analysis showed that there were more patients (33%) having PD-L1^+^ tumors (> 1% expression) responded to treatment than those (13%) with PD-L1^−^ tumors. In addition, the loss of expression of one or both HLA was associated with better PFS than when both HLA were expressed (1-year PFS, 30.9% vs. 5.6%, respectively, log-rank *P* = 0.01). The median PFS was 4.8 months (IQR, 2.7–14.0 months) and 1.8 months (IQR, 1.7–7.4 months), respectively. Interesting finding among the responders was that the plasma EBV of seven patients out of eight responders were detectable before treatment and already showed a decreasing trend during the first month of nivolumab treatment. Future study should confirm the observations in this study by recruiting larger cohort.

The safety of nivolumab was particularly assessed in a phase I/II clinical study enrolling patients with previously treated advanced or recurrent NPC and other solid tumors [430]. Patients were recruited if they fulfilled the criteria, including age ≥ 18 years old, ECOG performance status of 0 or 1, having progression disease after at least one prior line of systemic therapy, no central nervous system metastases, no prior malignancy with complete remission < 2 years, no autoimmune disease, no prior immunotherapy, no active tuberculosis infection, no pregnancy, and no immunosuppressive agent treatment. Nivolumab was given at 3 mg/kg once every 2 weeks for 8 weeks initially to 15 patients. With the absence of dose-limiting toxicity, the study proceed to cohort expansion that enrolled patients into three different cohorts with their respective cohort nivolumab regimens consisting of 3 mg/kg once every 2 weeks, 240 mg once every 2 weeks, and 360 mg once every 3 weeks, given in a 8-week cycle until disease progression or unacceptable toxicity for a maximum of 2 years.Nivolumab was demonstrated to be well-tolerated with 76% (out of total 46 evaluable safety) only experiencing grade 1–2 adverse events and only one patient discontinued due to toxicity associated to nivolumab. Three patients stopped the treatment due to adverse effects unrelated to nivolumab, whereas 35 other patients (76%) quit due to disease progression. More specifically for the NPC patients, the median follow-up time was 7.5 months (IQR, 0.8–24.7 months). Out of 32 NPC patients, 15 patients (47%) had reduction of tumor burden, four patients (13%) had partial response, and 17 patients (53%) had stable disease. Notably, six patients (19%) received the treatment for > 1 year. The median PFS was 3.5 months (IQR, 1.8–5.5 months), whereas the median OS was not reached. The 3-month OS and PFS were 87.5% and 64.2%, respectively. This study suggested that the flat doses of 240 mg and 360 mg were tolerable with minimal grade 1/2 toxic effects. This encouraging results need to be replicated in trials recruiting larger NPC patient population.

A clinical retrospective study reporting nivolumab efficacy in recurrent and metastatic NPC patients was conducted by Sato et al. [431]. Twelve patients were evaluated based on the inclusion criteria that the patients had distant metastasis or unresectable lesion after receiving radiotherapy alone or with concurrent chemotherapy; had distant metastasis at visit and had received chemotherapy; or had received platinum-based chemotherapy regimen. Nivolumab was given at 30 mg/kg or 240 mg by intravenous drip every 2 weeks until disease progression or unacceptable toxicity. The median follow-up was 11.9 months (IQR, 2.8–21.7 months). The study concluded that the 1-year OS and PFS were 75.8% and 33.3%, respectively with the median PFS of 2.8 months (IQR, 0.5–5.0 months). Of the 12 patients, two patients (16.7%) died of primary disease while one patient was lost to follow-up. The ORR was 16.7% with two patients (16.7%) having complete response, three patients (25%) having stable disease, and seven patients (58.3%) having progressive disease. There were several grade 1/2 adverse events observed in the patients (50%), including liver dysfunction, interstitial lung disease, anemia, hypothyroidism, arthritis, dermatitis, and myositis. Only one patient suffered grade 3 liver dysfunction, therefore nivolumab treatment was stopped promptly for this patient. Being yet another restrospective study analyzing low number of patients, the conclusion’s validity needs to be demonstrated further in prospective studies enrolling larger cohort.

In a single arm phase II trial nivolumab was combined with ipilimumab that targets cytotoxic T-lymphocyte protein 4 (CTLA4) in patients with recurrent or metastatic NPC. Eligible patients had measurable EBV DNA, had no more than one prior line of chemotherapy, ECOG performance status of 0–1, and adequate organ function. Nivolumab was administered at 3 mg/kg every 2 weeks and ipilimumab at 1 mg/kg every 6 weeks.,From the, 40 patients analyzed, 12 patients (30%) had best of response of partial response. The median OS and PFS were 17.6 months (IQR, 13.1–30.0 months) and 5.3 months (IQR, 3.0–6.4 months), respectively from a median follow-up of 17.3 months [432]. Treatment related adverse events such as maculopapular rash and hypothyroidism were commonly found from 34 patients 85% who suffered adverse events. Among those, 10% suffered grade 3/4 toxicities, such as hypocortisolism, pneumonia, myasthenia, gravis, and raised lipase.

In summary, the use of nivolumab is useful in treating previously treated recurrent and metastatic NPC patients for prolonging their survival, hence can be used as the second-line systemic treatment.

Also an antibody targeting PD-1, pembrolizumab has been assessed for its safety and antitumor activity in KEYNOTE-028 phase Ib trial treating patients with PD-L1^+^unresectable or metastatic NPC [386]. Patients were recruited based on eligible criteria such as those having tumor with PD-L1^+^ NPC, those who did or did not receive prior standard therapy or was deemed ineffective or inappopriate, those aged ≥ 18 years old, those with ECOG performance status of 0 or 1, and those with adequate organ function. Exclusion criteria included diagnosis of immunodeficiency or used of systemic corticosteroid; prior use of monoclonal antibody therapy, cancer therapy, or other immune checkpoint inhibitors; had active autoimmune disease, interstitial lung disease, other malignancy, or active central nervous system metastases. Pembrolizumab was administered at 10 mg/kg intravenously once every 2 weeks for 24 months or until disease progression, unacceptable toxicity, or refusal by patients. After the intial delay of treatment due to toxicity, treatment can be resumed if the adverse effect is reduced to grade 0 or 1 within 12 weeks of the last infusion, otherwise the treatment was discontinued. Among the 47 patients screened, 44 patients were evaluable for PD-L1. PD-L1 expression was confirmed in 41 patients (93.2%). Only 27 patients met enrollment criteria, hence were given pembrolizumab. Only three from these patients finished the 2-year treatment. The majority of patients (48.1%) withdrew due to progressive disease. Other reasons included adverse effects (18.5%), physician decision (7.4%), and patient decision (7.4%). The ORR was 25.9% with seven patients having partial response, 14 patients (51.9%) having stable disease, and six patients (22.2%) having progressive disease. The median follow-up time was 20 months (IQR, 2.2–26.8 months). The median time to response, duration of response (DOR), and duration of stable disease were 1.9 months (IQR, 1.4–16.4 months), 17.1 months (IQR, 4.8 to ≥ 22.1+ months), and 5.6 months (IQR, ≥ 1.7+ to  ≥ 12.9+ months), respectively. The median PFS was 6.5 months (IQR, 3.6–13.4 months). The 6- and 12-month PFS rates were 50% and 34.4%. The median OS, 6- and 12-month rates were 16.5 months (IQR, 10.1 months–not reached), 85.2%, and 63.0%, respectively. Twenty patients (74.1%) suffered adverse events with eight patients (29.6%) suffered grade ≥ 3 toxicities. The most common adverse events included rash (25.9%), pruritus (25.9%), pain (22.2%), hypothyroidism (18.5%), and fatigue (18.5%). Grade 3–5 adverse events were grade 3: pneumonitis (7.4%), proteniuria, anemia, hepatitis, and facial pain (3.7% each); grade 4: hepatitis and increased blood creatine phosphokinase level (3.7% each); and grade 5: sepsis (3.7%). The patient who suffered sepsis succumbed to drug-related death. Dose interruption with successful resolution occurred due to grade 1 upper respiratory tract congestion, cough, autoimmune hepatitis, and diplopia; grade 2 fatigue, arthritis, and herpes zoster; and grade 3 hepatitis. Immune-related adverse effects included hepatitis (14.8%), hypothyroidism (7.4%), and pneumonitis (7.4%). Immune-related adverse events also resulted to study discontinuation in patients, including proteinuria, penumonitis, hepatitis (3.7% each), and increased blood creatine phosphokinase level (7.4%). Interestingly, all seven patients having partial response after pembrolizumab treatment had PD-L1 expression that was found only in tumor, although there were 18 other patients who also had PD-L1 only positive in tumor. Overall, pembrolizumab was also effective in managing patients who were previously heavily treated. Despite the observed adverse events, pembrolizumab along with nivolumab were suggested to be safer in general than other PD-1 blockers in a comparative study of safety and efficacy [391].

Toripalimab as an alternative therapeutic agent for PD1-binding monoclonal antibody had been tested in several studies. In POLARIS-02 phase II clinical trial, 190 patients with recurrent and metastatic NPC were successfully recruited based on the criteria of age ≥ 18 years old, ECOG performance status of 0 or 1, refractory to prior standard chemoradiotherapy or disease progression within 6 months after adjuvant chemotherapy/chemoradiotherapy, adequate organ function, not using anticancer monoclonal antibody, not using any other anticancer therapy, no prior immune checkpoint inhibitors use, not using systemic corticosteroid therapy, no other malignancies, and not having active central nervous system metastases [244]. Patients were given 3 mg/kg intravenous infusion of toripalimab every 2 weeks until disease progression, development of intolerable toxicity, or voluntary withdrawal. From this patient population, 92 patients (48.4%) had at least two prior lines of systemic chemotherapy. One year after the last enrollment date, 94 patients (49.5%) died, 78 patients (41.1%) stopped the treatment, and 18 patients (9.5%) were still treated. The median treatment duration was 3.7 months (IQR, 0.2–34.8 months). The study reported an ORR of 20.5% and the disease control of 40.0%. The median time to response was 1.8 months (IQR, 1.8–2.1 months) with a DOR of 12.8 months (IQR, 9.4 months–not estimable). The median PFS and OS were 1.9 months (IQR, 1.8–3.5 months) and 17.4 months (11.7–22.9 months), respectively. Specifically, for 92 patients treated by at least 2 pior lines of chemotherapy, the ORR was 23.9% and the disease control was 41.3%. The median DOR, OS, and PFS were 21.5 months (IQR, 7.7 months–not estimable), 15.1 months (IQR, 10.4–20.4 months), and 2.0 months (IQR, 1.8–3.6 months), respectively. There were no differences in ORR between patients with different status of PD-1 (PD-1^+^, 27.1% vs. PD1^−^, 19.4%, *P* = 0.31). Albeit not statistically significant, patients with high expression of PD-L1 (> 25%) had higher ORR (38.1% vs. 19.3%), better median PFS (7.2 months vs. 1.9 months), and median OS (unreached vs. 15.1 months) than patients with low PD-L1 expression. The study also observed a significantly higher ORR in patients with ≥ 50% decrease of plasma EBV DNA load than those with < 50% decrease (48.3% vs. 5.7%, *P* = 0.0001). Poor outcomes from patients possessing genomic amplification of *11q13* region (including CCND1, FGF14, FGF3, and FGF4 genes) or *ETV6* genomic alterations (including 17 amplifications) may suggest that toripalimab is not suitable for them. Notably, eight patients (4.2%) with keratinizing NPC had an especially high ORR of 62.5%. There were 141 patients (74.2%) experiencing adverse events of any grade; 27 (14.2%) of them suffered grade 3–5 toxicities. Immune-related adverse effects included hypothyroidism (23.7%), hyperthyroidism (2.6%), abnormal liver function (1.6%), interstitial lung disease (1.6%), dermatomyositis (0.5%), and autoimmune myocarditis (0.5%). Other lower grade adverse events included anemia, AST/ALT increased, asthenia, proteinuria, leukopenia, pyrexia, pruritus, rash, and neutropenia.

In JUPITER-02 randomized, double-blinded phase III clinical trial assessing sequential toripalimab combination with gemcitabine and cisplatin and monotherapy toripalimab (146 patients) vs. gemcitabine plus cisplatin only and placebo regimen (143 patients) in chemotherapy-naïve patients with recurrent or metastatic NPC [433]. Toripalimab (240 mg) or placebo was given on day 1 along with gemcitabine (1 000 mg/m^2^) on days 1 and 8, and cisplatin (80 mg/m^2^) on day 1 every 3 weeks for six cycles, followed by monotherapy with toripalimab or placebo every 3 weeks until disease progression, development of intolerable toxicity, or completion of 2 years of treatment. The median treatment duration for toripalimab and placebo arms were 39 weeks and 36 weeks, respectively. A significant improvement of 1-year PFS (49% vs. 28%, respectively *P* = 0.0003) with median PFS duration of 11.7 vs. 8 months, *P* = 0.0003, respectively were observed. The ORR and median DOR were 77.4% vs. 66.4% (*P* = 0.033) and 10 months vs. 5.7 months, respectively. Similar incidence of adverse events of grade ≥ 3, those leading to discontinuation of treatments, as well as fatal adverse events were demonstrated in both arms, i.e., 89.0% vs. 89.5%, 7.5% vs. 4.9%, 2.7% vs. 2.8%, respectively, except for immune-related ones that were more common in toripalimab arm than placebo arm (in general, 39.7% vs. 18.9% and grade ≥ 3, 7.5% vs. 0.7%, respectively), although there were still manageable.

In conclusion, toripalimab is useful in improving the survival outcomes as both first line and second line therapy. It might also potentially be useful for specific subgroups, particularly patients with keratinizing NPC. More specific trial recruiting this particular subgroups is necessary to confirm its usefulness.

Yet another PD-1-targeting antibody called camrelizumab has demonstrated promising antitumor activity in NPC patients. In CAPTAIN phase II trial enrolling patients with recurrent or metastatic NPC who had at least two prior line of chemotherapy [34]. Camrelizumab was administered at 200 mg by intravenous infusion every 2 weeks. The median follow-up time was 9.2 months (IQR, 0.7–19.1 months). The ORR was 28.2% with one patient having complete response and 43 patients having partial response. The median DOR was not reached (IQR, 7.4 months–not estimable). The 12-month DOR rate was 53.7% with median OS and PFS of 17.1 months (IQR, 15.2 months–not estimable) and 3.7 months (IQR, 2.0–3.9 months), respectively. Adverse events occurred in 96.8% of the 156 enrolled patients with 14.1% suffering grade ≥ 3 adverse events and 10.9% of patients had serious toxicities. A couple of the major grade ≥ 3 adverse events included increased gamma-glutamyl transferase (3.2%) and anemia (3.2%). Treatment interruption and discontinuation also occurred in 18 patients (11.5%) and one patient (0.6%), respectively. One fatal case was considered to be drug-related.

In a randomized, double-blind phase III trial, camrelizumab was compared to regimen consisting placebo combined with gemcitabine and cisplatin in recurrent or metastatic NPC patients [434]. Patients were eligible if they aged 18–75 years old; had primary metastatic or local recurrence after curative radiotherapy, which was not amenable to local treatments; had not received previous systemic therapy for recurrent or metastatic disease for at least 6 months before the onset of disease progression; had ECOG performance status of 0 or 1; had an estimated life expectancy of at least 12 weeks; had at least one measurable lesion; and had adequate organ function. The exclusion criteria included if they had options for curative treatment available for their condition; central nervous system metastases; other malignancies (except already cured for more than 5 years ago); previous treatment with anti-PD-1 or anti-PD-L1 antibodies or CTLA4 inhibitors; medical conditions requiring the use of steroids or other immunosuppressive medications; a history of immunodeficiency disease; a history of non-infectious pneumonitis; active heaptitis B or C infection; active tuberculosis infection; or uncontrolled cardiac disease. Camrelizumab (134 patients) or placebo (129 patients) was given intravenously at 200 mg on day 1 plus 1000 mg/m^2^ gemcitabine on days 1 and 8, and 80 mg/m^2^ cisplatin on day 1 every 3 week for four to six cycles, followed by camrelizumab monotherapy or placebo on day 1 of 3-week cycle as maintenance therapy until disease progression, development of intolerable toxicity, voluntary withdrawal, or start of new anticancer treatment. Dose reduction could happen twice in case of intolerance before deciding to discontinue the treatment. The median follow-up time was 15..6 months (IQR, 12.3–19.2 months). The median number of treatment cycles was 16 for camrelizumab and 10 for placebo. The rate of participants for the planned six cycles of chemotherapy was 69% in camrelizumab group and 66% for placebo group. Twenty percent and 22% received four or less cycles from respective groups. Disease progression or death occurred in 58% and 78%, respectively in camrelizumab group and placebo group. Camerelizumab arm had better median PFS duration of 10.8 months (IQR, 8.5–13.6 months) when compared to placebo arm that had 6.9 months (IQR, 5.9–7.9 months) of median PFS duration (*P* = 0.0002). The 12-, 15-, and 18-month PFS rate was 45.8%, 38.4%, and 34.8%, respectively in camrelizumab arm and 20.5%, 14.8%, and 12.7%, respectively in placebo arm. The ORR was higher in patients with camrelizumab treatment (87.3% vs. 80.6%, respectively). The median DOR was also longer in camrelizumab arm (8.5 months, IQR, 6.9–11.1 months) than in placebo arm (5.6 months, IQR, 5.2–6.9 months). The instances of grade 3 or worse adverse events occurred at comparable rate in between the two arms of treatment regimen, i.e., 94% in camrelizumab group vs. 91% in placebo group. Ninety three percent and 90% cases of adverse events, respectively were treatment-related.. Some common adverse events observed were leukopenia (66% vs. 70%, respectively), neutropenia (64% vs. 66%, respectively), anemia (40% vs. 44%, respectively), and thrombocytopenia (40% vs. 40%, respectively). The occurrence of serious adverse events was slightly higher in the camrelizumab arm compared to placebo arm (44% vs. 37%, respectively). Treatment-related adverse events frequency occurred at 36% vs. 29% in camrelizumab arm vs. placebo arm, respectively. Treatment-related deaths occurred at 4% vs. < 1% in camrelizumab arm vs. placebo arm, respectively. Immune-related adverse events occurred at the rate of 84% vs. 50%, respectively. The grade 3 or worse of these events occurred at a lower rate at 15% and 1% in camrelizumab group and placebo group, respectively. The most common immune-related adverse events associated with camrelizumab treatment were reactive capillary endothelial proliferation (58%), hypothyroidism (43%), and rash (25%). This study also showed that early clearance of plasma EBV DNA was associated with longer PFS in patients receiving camrelizumab regimen whose baseline EBV DNA levels were positive, compared to patients whose EBV DNA remained positive after treatment. This indicates that EBV DNA could potentially be used as a predictor of response for treatment in patients with recurrent or metastatic NPC. In a comparative study, camrelizumab was considered as most efficacious PD1 inhibitor when used as second line therapy or used in first line when combined with chemotherapy [391]. In terms of safety, camrelizumab and nivolumab were considered safer options when considering grade ≥ 3 adverse events.

In conclusion, camrelizumab consistently demonstrated that it is useful for clinical application both acting as first line and second line therapy for recurrent or metastatic NPC patients. Future trial should consider establishing a longer follow-up for demonstrating its long-term survival benefit.

One of the newest PDI inhibitor, spartalizumab, has been tested in randomized phase II trial in patients with recurrent or metastatic NPC comparing to chemotherapy regimen [389]. This open-label randomized controlled study recruited patients who aged ≥ 18 years old, had non-keratinizing recurrent or metastatic NPC and progressed on or after platinum-based chemotherapy treatment. Spartalizumab was administered intravenously in 82 patients at 400 mg every 4 weeks until disease progression, unacceptable toxicity, or discontinuation due to patient or physician’s decision. Patients treated in the chemotherapy arm (39 patients) either received monochemotherapy (69.2%) or combination of two or more chemotherapy (30.8%). All patients had received prior anticancer treatment before enrollment. Among the patients enrolled in spartilizumab arm and chemotherapy arm, majority had received radiotherapy (84.1% vs. 92.5%, respectively) and ≥ 2 prior lines of systemic therapy (80.5% vs. 77.5%, respectively). Cisplatin was the most commonly given systemic therapy in both arms (84% vs. 82.5%). The ORR of the last line of prior therapy was 28.1% and 32.5% for spartalizumab arm and chemotherapy arm, respectively. Progressive disease occurred at a rate of 34.1% vs. 20%, respectively. The median duration of treatment was 14.4 weeks (IQR, 3.1–120.1 weeks) and 19.3 weeks (IQR, 3.0–77.4 weeks), respectively for spartalizumab arm and chemotherapy arm. There were 27 patients (32.9%) and 18 patients (46.2%) in respective group receiving treatments for > 24 weeks. Patients who initially were treated with chemotherapy and moved to spartalizumab arm was referred as crossover group. This group combined with the spartalizumab group formed all-spartalizumab group. This combined group had 33 patients (30.8%) treatment exposure of > 24 weeks. The OS was assessed by an intention-to-treat analysis, therefore patients in the crossover group was considered as patients in the chemotherapy arm for this particular survival statistic. The trial demonstrated that the median OS and PFS of spartalizumab arm vs. chemotherapy arm were 25.2 months (IQR, 13.1 months–not estimable) vs. 15.5 months (IQR, 8.3–21.3 months) (*P* = 0.138) and 1.9 months (IQR, 1.8–3.6 months) vs. 6.6 months (IQR, 3.7–9.3 months) (*P* = 0.915), respectively. The median PFS of crossover group was 1.7 months (IQR, 1.6–1.9 months). The ORR were 17.1% vs. 35%, respectively with median DOR of 10.2 months (IQR, 7.4 months–not estimable) vs. 5.7 months (IQR, 3.7–7.4 months), respectively. The disease control rate was 42.7% and 70%, respectively in spartalizumab and chemotherapy groups. Most patients in all-spartalizumab group (96.3%) and chemotherapy group (94.9%) experienced adverse events. Suspected treatment-related toxicities were at 65.4% and 87.2% in the two groups, respectively. The occurrence of grade 3/4 treatment-related toxicities was lower in all-spartalizumab group (16.8%) compared to chemotherapy group (41%), and such serious adverse effects were slightly lower also in the all-spartalizumab group (11.2% vs. 17.9%, respectively). Adverse events that led to treatment discontinuation occurred at a lower rate in all-spartalizumab group compared to chemotherapy group (1.9% vs. 10.3%, respectively). There were five cases of death on treatment, but they were not treatment-related. A couple of common adverse events (mostly grade ≤ 2) observed in all spartalizumab arm were fatigue (10.3%) and pruritus (9.3%). Although the overall performance of PFS was lower, spartalizumab may be more beneficial than chemotherapy when considering its ability in prolonging OS and DOR in addition to its less toxic nature. In this study, plasma EBV DNA was also shown to be a response predictor. Both in spartalizumab and chemotherapy arms, the ORR was higher in patients with EBV DNA levels < the weighted median level that those with EBV DNA levels ≥ the weighted median level (22.2% vs. 11.1% in spartalizumab arm and 42.9% vs. 29.4% in chemotherapy arm, respectively). RNA-sequencing data also showed that there was a negative correlation between the response of spartalizumab and the expression of T cell immunoglobulin and mucin domain-containing protein 3 (*TIM-3*), lymphocyte activation gene 3 (*LAG-3*), and *IFNγ* signature gene at baseline. More study is necessary to validate this novel finding.

All of these suggested that immune checkpoint inhibitors are not only useful as monotherapy but also beneficial when used in combinatorial manner. However, a lot of works still need to be done to demonstrate long-term survival benefit as well as finding novel inhibitors that are associated with lower toxicity without compromising their efficacies, especially for the notoriously difficult to treat NPC patients with recurrence and metastatic disease.

#### Adoptive immunotherapy

Adoptive immunotherapy involves the transfer of active immune components like NK cells or CTLs to NPC patients. In an open-label non-randomized phase II trial, EBV-specific CTL was developed and transferred to patients with recurrent or metastatic NPC [40]. Such treatment setting was combined with chemotherapy consisting of gemcitabine and carboplatin for assessment of safety and efficacy. The inclusion criteria for the study included if patients had no active or severe cardiac, pulmonary, or cerebrovascular disease; had adequate organ function; and had no HIV infection. Venesection was performed on patients to take 300 mL of peripheral blood for the generation of lymphoblastoid cell line (LCL) and EBV-CTL. Patients were administered with 1 000 mg/m^2^ gemcitabine and carboplatin (AUC 2) on days 1, 8, and 15 every 4 weeks for four cycles. When necessary, two more cycles were added to allow sufficient time for CTL generation. After 2–4 weeks from the last treatment course, EBV-CTLs were administered at 1 × 10^8^ cells/m^2^ on weeks 0, 2, 8, 16, 24, and 32. Half of the recruited patients (19 patients) had distant metastatic disease, 23.7% (9 patients) had metastatic disease at locoregional sites, and 26.3% (10 patients) had both. Majority of the patients (97.4%) had type III NPC. EBV-CTL lines were successfully generated in 37 patients with the median time taken to produce and release the first dose of 13 weeks (IQR, 8–22 weeks). The EBV-CTL lines were predominantly CD8^+^ T-cells plus other T-cells such as effector memory, late effector memory, and central memory T-cells. When evaluating 35 cell lines, the cells were specific for immunodominant EBV antigens (BZLF1, BRLF1, BRMF1, or EBNAs 3A, B, C). LMP2-specific T-cells were found in 26 cell lines; LMP1-specific T-cells were found in 8 cell lines and those targeting EBNA1 in 3 cell lines. Induction chemotherapy was completed as planned in 31 patients and with additional cycles (up to six) in three patients. Four patients did not complete the chemotherapy due to either progressive disease or death on treatment. Of the 35 patients receiving EBV-CTLs, 24 patients (68.6%) completed all six cycles with the median CTL dose of 9.6 × 10^8^ cells (IQR, 6.3–10.3 × 10^8^ cells). The rest of patients did not receive fullcycle of CTL administration due to disease progression. The median follow-up duration was 29.9 months.The ORR and CBR rates of 38 patients receiving chemotherapy before the administration of CTLs were 63.2% and 94.7%, respectively with three patients (7.9%) having complete response, 21 patients (55.3%) having partial response, and 12 patients (31.6%) having stable disease. Twelve patients (31.5%) were still alive, two (5.3%) of which displayed no evidence of disease progression. One patient (2.6%) had disease progression on chemotherapy and one (2.6%) was not evaluable. Out of 35 patients who received CTLs after chemotherapy, 2 patients (5.7%) having complete response, 13 patients (31.7%) having partial response, and 7 patients (20%) having stable disease, which translates to ORR and CBR of 42.9% and 62.9%, respectively. With a median OS of 29.9 months (IQR, 20.8–39.3 months), the 1-year, 2-year and 3-year OS rates for the 35 patients who underwent chemotherapy and CTL treatments were 77.1%, 62.9% and 37.1%, respectively. The median PFS was 7.6 months (IQR, 7.4–8.4 months). At 1-year mark, there were still 25.7% patients being free of disease progression. The median PFS for CTL phase was 3.7 months (IQR, 2.0–35.3%). A subgroup of 25 patients receiving CTLs specific for LMP2 displayed better OS than 9 patients who received CTLs lacking LMP2 specificity. Grade 3 or worse toxicities occurred during the chemotherapy phase. Two out of three patients who suffered severe effects died of aspiration pneumonia and neutropenic sepsis (one patient) and bacterial meningitis due to tumor invasion to the brain (the other patient). One other patients had grade 3 epistaxis secondary to grade 3 thrombocytopenia but was resolved. CTLs were well-tolerated with no grade ≥ 3 adverse events.The most common toxicities were grade 1 and 2 fatigue, grade 1 rash, and grade 1 myalgia. In this study, the EBV DNA load was found to correate with tumor burden, but no correlation was found between the baseline EBV DNA and response to therapies. For other biomarkers such as the baseline cytokine levels of interferon gamma-induced protein 10 (IP-10) and macrophage inflammatory protein-3 alpha (MIP-3α) were shown to negatively correlate with long-term survival (*P* = 0.029 and *P* = 0.035, respectively).

Recently, a case report reported an NPC patient with metastatic disease receiving EBV-specific CTL combined with PD1 blockade therapy nivolumab [435]. The patient’s initial diagnosis was T4N2M0 poorly differentiated NPC. The patient received chemoradiotherapy consisting of volumetric modulated arc therapy (VMAT) and high dose cisplatin. After 3 months, metastasis was found and patient was given SBRT and then palliative chemotherapy consisting of carboplatin plus gemcitabine after 1 month. The patient was then enrolled to the prospective study testing EBV-specific adoptive T-cell therapy. Venesection was performed to collect peripheral blood mononuclear cells (PBMCs) to generate EBV-specific T-cells. From the 8 × 10^8^ T-cells, 22% were CD3^+^CD8^+^ cells, 21.6% of which displayed EBV-specific reactivity. Sixty eight percent were CD3^+^CD4^+^ cells. The patient received six doses of 4 × 10^7^ T-cells containing 1.9 × 10^6^ EBV-specific T-cells per dose every 2 weeks. Soon after the completion of CTL therapy, the patient was given 240 mg nivolumab every 2 weeks for 21 cycles. After the completion of four cycles of nivolumab, PET scan revealed complete resolution of active disease. Scans beyond the completion of immune checkpoint inhibitor treatment showed no evidence of relapse. The patient displayed a complete resolution of metastatic disease with relapse-free duration of 22 months. Such response correlated well with the presence of high expression of PD-L1 in tumor; 80% of tumor cells were PD-L1 positive. When looking at the load of plasma EBV DNA, its level followed the pattern of disease activity; EBV DNA load decreased following chemotherapy and was undectectable after 3 weeks after the start of nivolumab therapy. This persisted for more than 250 days after nivolumab treatment. Such favorable observation should be replicated in trials with large cohort.

NK cell transfer was also recently reported in a case study with the combination of chemoradiotherapy in a patient with recurrent NPC and intracranial metastasis [39]. The patient was diagnosed with T4N1M0 non-keratinizing undifferentiated NPC. CCRT consisting of paclitaxel and nedaplatin concurrent chemotherapy combined with nimotuzumab was used to treat the patient. Upon subsequent reduction of tumor size, the patient came back for more treatment for recurrent disease after just 2 years. The regimen prescribed was CCRT consisting of gemcitabine, cisplatin, and IMRT. In one and half a year time, intracranial metastases were found. Gemcitabine and cisplatin were again given to patient plus capecitabine for maintenance. Three months from the start of the last treatment, the patient started to receive NK cell therapy using umbilical cord blood as the source. A dose of 2 × 10^9^ CD56^+^/CD3^−^ cells was given intravenously three times a year, up to the year of report. Six months after therapy, no evidence of efficacy was shown. However, intracranial metastasis gradually decreased 31 months after the onset of treatment with partial disappearance of the metastases and the decrement continued until 42 months after the onset of treatment. The toxicities were mild, although caution should be exercised when receiving allogeneic NK cells. This strategy should be evaluated further in large clinical trial to confirm its efficacy.

In summary, the adoptive immunotherapy seemed to demonstrate delayed tumor response but was able to mitigate the disease progression even in patients who already have metastases at multi-sites. Some subgroups of patients even achieved prolonged survival with no requirement for systemic therapy for approximately 3 years. The use of immune checkpoint inhibitors along with adoptive transfer of CTL or NK cells may mount even better clinical efficacies, and also for more rapid and lasting disease resolution.

#### Active immunotherapy

The most prominent clinical study for active immunotherapy involves the development of a recombinant Modified Vaccinia Ankara (MVA) vaccine that encodes EBV target antigens. In a phase I trial, patients were vaccinated against the EBNA1 and LMP2 using the inactive fusion of those antigens (MVA-EL) in Chinese EBV-positive NPC patients [403]. Patients having poorly differentiated NPC who were enrolled into this study had to fulfill several criteria, including ≥ 12 weeks following completion of first line treatment and were in remission; aged ≥ 18 years old; free from > grade 1 toxicities; using adequate birth control; ECOG performance status of 0 or 1; life expectancy of > 4 months; adequate organ function; no active hepatitis B/C and HIV infection; no autoimmune or skin disease requiring therapy; and no active infection, severe egg allergy, splenic dysfunction, previous myeloablative or current immunosuppresive therapy. Three intra-dermal vaccinations of MVA-EL were given at 3 weeks interval. There were five sequential doses examined, i.e., 5 × 10^7^, 1 × 10^8^, 2 × 10^8^, 3.3 × 10^8^, and 5 × 10^8^ plaque forming units per vaccination across a cohort of three patients in each dose, except there were six patients in the highest dose. All patients had received prior radiotherapy and 14 of them also received chemotherapy. All patients were clinically disease-free and the median time from last treatment to the first vaccination was 20 weeks (IQR, 14–42 weeks). All patients received their planned dose and the five levels of increasing doses was executed without dose-limiting toxicity. Adverse events were only observed as grade 1 at most and rarely as grade 2 and 3. Three common side effects occurred occasionally in all dose settings were fatigue, flu-like symptoms, and arthralgia. In patients treated with dose level 4 and 5, myalgia was reported also as a side effect. Using ELIspot, the T-cell response to EBNA1 and/or LMP2 was assessed and was found increased dose-dependently after vaccinations, especially after the second cycle and with stronger response seen in the highest dose tested. The response seen in the 15 patients out of 18 patients could be mapped, in many cases, to known CD4 and CD8 epitopes in EBNA1 and/or LMP2. The study suggested benefits of using vaccination strategy on the induction of CD4^+^ and CD8^+^ T-cells to recognize the EBV antigens and demonstrated its general safe of use.

In another phase I clinical trial enrolling United Kingdom’s EBV-positive NPC patients, MVA-EL was similarly tested [402]. Patients were recruited based on the criteria that they were in complete (or unconfirmed complete) remission at least 12 weeks post-completion of first line treatment; had toxicity resolved to grade ≤ 1; aged ≥ 18 years old; were using adequate birth control; had life expectancy > 4 months; had ECOG performance status of 0 or 1; had adequate organ function; had no active hepatitis B/C or HIV infection; had no autoimmune or skin disease requiring therapy; had no active infection; had no history of allergy of egg; and had no history of myeloablative therapy, splenic dysfunction, and not undergoing immunosuppressive therapy. The same vaccine as previous study (MVA-EL) was given intradermally three times at a 3-week intervals. The same five level doses were employed with a 3 + 3 cohort in each dose. Sixteen patients who received the vaccination were previously treated either with radical locoregional radiotherapy (four patients) or chemoradiotherapy (12 patients), with some (10 patients) also received adjuvant chemotherapy. There were six strains of EBV identified across eight patients examined. All vaccinated patients suffered self-limiting injection site reaction which was mostly grade 1, except that it was grade 2 for seven cases. Due to the grade 2 advese effect, the cohort was expanded in dose level 1. Nine patients suffered systemic toxicities, five cases of which were grade 2 regional pain and lymphadenopathy, fatigue, and flu-like symptoms. One patient withdrew from the study. Patients who had residual disease and received more than one line of prior therapy had a detectable plasma EBV DNA either before or 4 weeks after vaccination. Of those, two patients had sustained rises of EBV DNA due to progressive disease and one patient had the level rose and fell transiently during the course of vaccination but became stabilized and went below baseline later before disease relapse became apparent at 25 months after the onset of vaccination. Vaccination did not change the counts of lymphocytes much. The proportions of CD3^+^CD4^+^, CD3^+^CD8^+^, and CD4^+^ forkhead box P3 (FOXP3)^+^ remained comparable to those of healthy individuals. The ELIspot assays demonstrated that there was low level of recognition of antigen or epitope of EBNA1 and LMP2 in 11 patients (73%) and 10 patients (66%) pre-vaccination, respectively, but more than twofold increase of recognition after vaccination in 7 patients (50%) and 6 patients (43%), respectively. The vaccination also improved the functional diversification of both CD4^+^ and CD8^+^ T-cells indicated by the increased of polyfunctional T-cells producing TNFα, INFγ, and IL-2, and/or monofunctional T-cells producing macrophage inflammatory protein-1 beta (MIP-1β) in specific populations targeting EBNA1 and LMP2. Importantly, the developed MVA-EL vaccine was also shown to provide immunogenicity to individuals from diverse ethnic backgrounds, hence is favorable for worldwide deployment.

Although both trials were performed in patients who were in remission, the studies noted modulation of immune response needed for cancer clearance, including fluctuation of circulating EBV genome level and the magnitude of induction of the effector cytokines of T-cell response.

Immunotherapy has been useful in prolonging the survival outcome of patients, especially those with recurrence and metastatic disease. However, the consequence of messing with the immune system is that it can react back to our own body resulting to the observed immune-related adverse events. Because the exact mechanisms of immunotherapy, especially the immune checkpoint inhibitors is still not fully understood, the underlying mechanism of the immune-related adverse events should be further studied and checkup of potential toxicities should be regular, so that appropriate strategy can be implemented in time [436]. This can ensure the patients to tolerate and continue to benefit from immunotherapy. In response to the need to achieve a long-lasting response of immunotherapy, combinatorial approach can be done between immunotherapies or with other form of therapies such as targeted therapy and other novel therapies not mentioned in this review, i.e., oncolytic virus and nanoparticles. Data demonstrating long-term durability, resistance development, and potential late toxicities are still lacking for immunotherapy. Hence, further studies should conduct longer duration of follow-up in view of establishing efficacy and toxicity record for immunotherapy in NPC patients.

The above completed studies indicate that clinical studies are actively testing drugs to improve treatments for NPC patients by employing different type of therapies, assessing different doses, assigning different regimens, and implementing all of these in different patient populations. Some recruiting and ongoing clinical trials are already established to further study the promising drugs listed above in NPC patients, including axitinib, camerelizumab, capecitabine, cetuximab, endostar, nimotuzumab, nivolumab, pembrolizumab, proton therapy, and toripalimab (Table 6).

**Table 6:** New clinical studies evaluating cancer therapeutics for nasopharyngeal cancer patients

Drug	Identifier no	Trial status	Trial phase	Control arm	Experiment arm	Patient enrolled
Axitinib	NCT04562441	Recruiting	II	n.a	Axitinib + avelumab	R/M NPC
Camerelizumab	NCT04944914	Recruiting	III	Camrelizumab	Camrelizumab + SBRT	Metastatic NPC
	NCT03707509	Ongoing	III	Placebos + gemcitabine + cisplatin	Camrelizumab + gemcitabine + cisplatin	R/M NPC chemo-naïve
	NCT05011227	Recruiting	II	n.a	Camrelizumab + chemotherapy + endoscopic surgery	Recurrent NPC
	NCT04586088	Recruiting	II	n.a	Camrelizumab + apatinib	R/M NPC
	NCT04453826	Recruiting	III	IC (gemcitabine + cisplatin) + CCRT (cisplatin + IMRT)	Camrelizumab + IC (gemcitabine + cisplatin) + CCRT (cisplatin + IMRT)	High risk NPC
	NCT04782765	Recruiting	II	n.a	IC (camrelizumab + cisplatin) + CCRT + maintenance therapy (camrelizumab)	NPC with no distant metastasis
	NCT03427827	Recruiting	III	Chemoradiotherapy + best supportive care	Chemoradiotherapy + camerelizumab	Locoregionally advanced NPC
Capecitabine	NCT02958111	Ongoing	III	No intervention	capecitabine	Locoregionally advanced NPC
	NCT02973386	Recruiting	III	CCRT (IMRT + cisplatin)	CCRT (IMRT + cisplatin) + capecitabine	Locally advanced high risk NPC
	NCT04220528	Recruiting	II	n.a	IC (gemcitabine) + CCRT (IMRT + nidapatin) + capecitabine/teggio	N3 NPC
Cetuximab	NCT02633176	Recruiting	III	IC (cisplatin + docetaxel) + CCRT (cisplatin) + maintenance therapy (capecitabine)	IC (cetuximab + cisplatin + docetaxel) + CCRT (cetuximab + cisplatin) + maintenance therapy (capecitabine)	Untreated metastatic NPC
Endostar	NCT02636231	Ongoing	II	IMRT	Endostar + IMRT	Locally recurrent NPC
Nimotuzumab	NCT03666221	Recruiting	II	n.a	Nimotuzumab + IMRT	Recurrent NPC
	NCT04456322	Recruiting	III	IC (paclitaxel) + CCRT (cisplatin + IMRT)	IC (paclitaxel) + CCRT (nimotuzumab + RT)	Locoregionally advanced low risk NPC
	NCT04223024	Ongoing	II	IC (paclitaxel) + CCRT (cisplatin + IMRT)	IC (paclitaxel) + CCRT (cisplatin + IMRT + nimotuzumab)	Advanced high risk NPC
	NCT03915132	Recruiting	II	n.a	Nimotuzumab + VMAT (IMRT)	Elderly NPC
	NCT03708822	Recruiting	II	n.a	Docetaxel + cisplatin + nimotuzumab	R/M NPC
	NCT03837808	Recruiting	III	CCRT (IMRT + cisplatin)	IMRT + concurrent nimotuzumab	Stage II and III NPC
Nivolumab	NCT03097939	Recruiting	II	n.a	Nivolumab + ipilimumab	EBV-driven NPC
	NCT03267498	Recruiting	II	n.a	Nivolumab + CCRT (cisplatin)	Stage II–IVB NPC
Pembrolizumab	NCT03734809	Ongoing	II	n.a	IC (pembrolizumab + gemcitabine + cisplatin) + CCRT (cisplatin + pembrolizumab) + maintenance therapy (pembrolizumab)	Untreated stage IVA NPC
	NCT02538510	Ongoing	I, II	n.a	Pembrolizumab + vorinostat	R/M NPC
	NCT03849469	Ongoing	I	XmAb22841	XmAb22841 + pembrolizumab	Advanced NPC
	NCT03674567	Recruiting	I, II	FLX475	FLX475 + pembrolizumab	Advanced NPC
Proton therapy	NCT04870840	Recruiting	I	n.a	Image-guided hyper-fractioned proton therapy	Locally Advanced NPC
Toripalimab	NCT04398056	Ongoing	II	n.a	Chemoradiotherapy (fluorouracil + cisplatin + IMRT) + toripalimab	De novo metastatic NPC
	NCT04376866	Recruiting	III	CCRT (cisplatin + IMRT)	CCRT (toripalimab + cisplatin + IMRT) + toripalimab	Locoregionally recurrent NPC
	NCT04517214	Recruiting	II	Gemcitabine + cisplatin + IMRT + maintenance therapy (capecitabine)	Toripalimab + gemcitabine + cisplatin + IMRT + maintenance therapy (capecitabine + toripalimab)	Metastatic NPC
	NCT04992988	Recruiting	II	n.a	Toripalimab + CCRT (cisplatin)	Locoregionally recurrent NPC
	NCT04778956	Recruiting	III	Salvage surgery	Salvage surgery + toripalimab	Resectable locally recurrent NPC
	NCT03925090	Recruiting	II	CCRT (cisplatin)	CCRT (cisplatin + IMRT + toripalimab) + toripalimab	Locoregionally advanced high risk NPC
	NCT04405622	Ongoing	II	n.a	Toripalimab + gemcitabine + maintenance therapy (toripalimab)	R/M NPC
	NCT04453813	Recruiting	III	CCRT (cisplatin + IMRT)	CCRT (toripalimab + cisplatin + IMRT) + toripalimab	Unresectable locally recurrent NPC
	NCT03854838	Ongoing	II	n.a	IMRT + toripalimab	Unresectable locally recurrent NPC
	NCT02915432	Ongoing	I, II	n.a	First line chemotherapy + toripalimab	Advanced NPC
	NCT03907826	Recruiting	III	Chemoradiotherapy (gemcitabine + cisplatin + IMRT)	Toripalimab + chemoradiotherapy (gemcitabine + cisplatin + IMRT)	Recurrent NPC
Toripalimab	NCT03930498	Recruiting	II	n.a	Toripalimab + chemoradiotherapy (gemcitabine + cisplatin + IMRT)	Recurrent high risk NPC
	NCT03474640	Ongoing	I	n.a	Toripalimab	Advanced NPC

*CCRT* concurrent chemoradiotherapy, *EBV* Epstein–Barr virus, *IC* induction chemotherapy, *IMRT* intensity-modulated radiotherapy, *R/M NPC* recurrent or metastatic nasopharyngeal cancer, *RT* radiotherapy, *SBRT* stereotactic body radiation therapy, *VMAT* volumetric modulated arc therapy

Based on the studies above, treatment decision seems to be still being guided by solely the past performance of treatment modalities/regimens and disease status of the patients. With some patient stratification such as by disease stage and treatment status, targeted therapy drugs like those inhibiting EGFR is used in combination for the purpose of preventing resistance development in general. This motivation makes sense because heavily treated patients inevitably face progressive disease due to the emergence of CSCs, hence justifying the inhibition of the commonly-associated signalling pathways. However, tumor heterogeneity in NPC patients is a problem that complicates disease situations and makes treatment outcomes to be inevitably variable. There was not even a definitive remission despite that drugs were developed based on a specific target protein. When study attempted to stratify patient based on the presence/level of expression of the target protein in tumor like in the case for cetuximab, the best outcome was only 11.7% partial responders [417] and 34.9% long-term survivors [419] as found by two separate studies. Not only that, there were only 33% patients bearing PD-L1+ tumor responded to nivolumab treatment [385] and 28% of such patients being partial responders in pembrolizumab treatment [386]. In the context of PD-1/PD-L1 signalling, targeting PD-L1 might have been a wiser choice [437].

Better response-predicting biomarkers are obviously needed to better guide personalized medicine moving towards the implementation of precision medicine. For EBV^+^ NPCs, plasma EBV DNA load could at least in some way used to track the disease status apart from its use as diagnostic tool, but for other NPCs, novel molecule should be discovered. At this moment, the transition point is to start using genomic profiles and transcriptomic profiles to complement the clinical presentation of NPC patients besides the information regarding lifestyle, environment, and symptoms. From the traditional clinical trials, molecular profiles from exceptional responders can be analyzed and suitable biomarkers can be selected for patient stratification and then biomarkers can then be reassessed on how good they are as response predictors. Data of such associations between drugs and biomarkers are very much needed to established precise prescription of medication.

The ideal state of precision medicine is to make comprehensive use of omics technologies. There are integrations of omics data encompassing the genomics, transcriptomics, proteomics, epigenomics, metabolomics, and microbiomics with unique individual’s situations [438]. By using algorithms made sense by the artificial intelligence, these data can get associated to available pharmacokinetics and pharmacodynamics data of drugs to yield comprehensive predictions of efficacies and toxicities at different doses. When such data available in excess, machine learning can consume such data to form big knowledge and then help making automated decision of cancer therapy.

Current practice of making individualized treatment decision still relies on the availability of actionable targets. There is stilla lacking in the availability of drugs to match each and every potential therapeutical target. Hence, the closest initiative we get to the ideal precision medicine today is the existence of multidisciplinary molecular tumor boards (MTBs) that serve as the unifying platform to achieve a profile-based, patient-tailored consensus recommendation based on the identification/prioritization of genomic alterations and potential drug actionability and the assessment of availability of clinical trials or compassionate use drugs for eligible patients [439]. As also expected in precision medicine, MTBs can make recommendation for both diagnostic and therapeutic purposes. MTBs’ members consist of oncologists, pathologist, and clinical scientists in molecular pathology. When necessary, clinical geneticists and bioinformaticians can join in to support interpretation of large-scale sequencing data.

An example for MTB recommendation workflow performed in University Medical Center Groningen of The Netherlands for rare or complex mutational profiles of non-small cell lung carcinoma (NSCLC) patients is delineated in the following [440]. Review request is typically submitted by treating physician, pathologist, or clinical scientist. Clinical scientist annotates the patient’s molecular profile. Somatic variants are annotated according to the Human Genome Variant Society recommendations for the description of sequence variants. Variants are classified as single nucleotide polymorphisms (SNPs) on the basis of the variant allele frequency in combination with a database search consulted for known SNPs, such as in dbSNP, ExAC, GnomAD, and the 1000 Genomes Browser. Actionability of oncogenic variants is tiered according to the 2017 American College of Medical Genetics and Genomics (ACMG)/American Society of Clinical Oncology (ASCO)/College of American Pathologists (CAP) guidelines, by consulting knowledge databases, such as in cBioPortal, CIViC, ClinVar, COSMIC, JAX-CKB, and OncoKB, and by a systematic review of the literature. Assessment is also based on prior experience. Structural biologists can be involved in the case of rare or unknown variants to seek reference from molecular modeling for the testing of binding affinities even for drugs that are not indicated for the assessed disease. Decision needed for first-line treatment choice or for progressive disease often affects the direction of assessment. For the discussion of first-line choice of therapy, guide-line based therapeutics are usually followed. When it is not directly apparent, patients may be enrolled to clinical trials, even for non-targeted therapy trials. Otherwise off-label targeted therapy may be considered based on the availability of evidence-based prescription from 2017 ACMG/ASCO/CAP guidelines. Treating physician then considers the recommendation with other considerations, such as performance status, comorbidities, and drug availability. In a retrospective study, high adherence to targeted therapy recommended by MTB had led to high ORR and long-lasting PFS and OS in NSCLC patients with rare or complex mutational cancer profiles analyzed [440]. To ensure an even higher level of consistency for the recommendation provided by the MTB, a support system has been established to bring the realization of precision medicine even closer [441].

There are also concerns associated with the adoption of precision medicine. Since more advanced tools and more individualized plans are used starting from diagnoses to therapeutic decisions, it could be associated with more cost along the line, i.e.,molecular test, data storage, and data processing cost. Certainly, a realistic comparison of cost between the traditional way of treating patients and the protocol of executing precision medicine should be presented down the line in order to judge whether the new implementation of precision medicine worths the cost and is beneficial for prolonging patients’ treatment outcomes. Whole genome sequencing of one sample alone generates about 200 gigabytes of data [438]. That much of information generated per patient combining with other sets of data will quickly overload the data storage and will undoubtedly require computer with higher processing power to read the data more quickly. Dilemma about the ethical issues from the data obtained from patients is also a concern [442, 443]. We are definitely worried that there is an overwhelming amount of information about us that is not within our control that may be susceptible to data breach risk. Not to say that there is small chance of false positive or negative that will directly influence the treatment decision in precision medicine. This could amplifies to unnecessary feeling of anxiety that might emerge due to the prediction of disease that might happen in the future or incurring of unnecessary medical intervention cost. In financial aspect, susceptibility to certain disease identified through molecular predictions will likely disqualify patient from having insurance plan or coverage. Being aware of the potential ethical and financial risks of implementing precision medicine could definitely further help us to create an accommodating and reliable system.

## Challenge and future outlook

The main challenge of treatment management for advanced stages NPC patients is the complexity of the tumor biology which is attributed by the heterogeneity, diverse epigenetic pattern and distinct molecular aberrations associated with self-renewal, proliferation, migration and invasion. The tumor complexity is also conferred by the interactions between host and environmental factors. Cellular evolution in tumor have further amplified its complexity. In view of this, personalized medicine has gained the greatest interest in NPC management including diagnosis and treatment. It is a promising approach that seeks to tailor treatment to each individual patient based on the genetic makeup of their tumor and other personalized factors.

Radio- and chemo-therapy remain useful for patients, especially those with early stage of NPC. However, aggressive radio- and chemo-therapy are needed for advance stages of NPC which are associated with fatal toxicity. In future, the precision of radiotherapy with more refined plan and delivery in individual can hopefully be achieved with the emergence of image-guided radiotherapy (IGRT), adaptive radiotherapy (ART), intensity-modulated carbon ion therapy (IMCT), and IMPT. Further comparative and randomized studies using these novel technologies in prospective clinical trial with IMRT are required to confirm their efficacy and toxicities. Despite that, cancer therapy has slowly shifted from using cytotoxic therapy to a more targeted approach with the inclusion of targeted therapy and immunotherapy which respectively aim specifically on a cellular protein target. This shift has prompted researchers to evaluate whether segregating patients based on the positivity/degree of expression of molecular target is helpful. Observation thus far only saw additional benefits in some cases. However, certain subgroup of patients or certain mode of application, i.e., whether applied as induction therapy or combined with chemoradiotherapy can modify the efficacy of specific drugs, hence it may be advantageous when segregating patients in this manner. The overview of cancer therapy discussed in current review conclude that targeted therapies are the most efficient option. Nevertheless, immunotherapy is still required further studies to compare its efficacy with the current standard care. Collectively, with the rapid development of precision medicine, optimized combination of immunotherapy, targeted therapy, precision radiotherapy or chemotherapy according to the individual patient’s cancer stage, genomic characteristic and immune status to customize the treatment may ultimately enhance the treatment outcome for advanced stage of NPC patients.

Precision medicine in cancer, although a promising approach, it has both opportunities and challenges. First, there is lack of understanding of the genomic basis of cancer. Despite advances in genome sequencing and other technologies, our understanding of the genomic basis of cancer is still limited. This can make it difficult to develop targeted treatments that are effective for all patients with a particular type of cancer. Second, cancer is a highly heterogeneous disease, with each tumor possessing its own unique genomic alterations. This make it become more challenging to develop a one-size-fits-all treatment approach for each type of cancer. Third, there is limited availability of targeted therapies. Despite advances in our understanding of the genomic basis of cancer, there are still relatively few targeted therapies available for the treatment of many types of cancer. This has also limited the ability of precision medicine to improve patient outcomes. Fourth, genomic testing is essential for the implementation of precision medicine in cancer, but it can be expensive and may not be readily available to all patients. However, this could be solve by increasing medical insurance coverage. Besides that, the clinical trial design is challenging as precision medicine in cancer required a different approach to clinical trial design compared to traditional trials. For example, it may be necessary to enroll smaller patient population and to use more complex endpoints to evaluate treatment efficacy. Moreover, low implementation is the main obstacle for precision medicine. Precision medicine in cancer must be integrated with the existing standard of care in order to be widely adopted and to improve patient outcomes. Lastly, sharing of patient data and samples is crucial for the advancement of precision medicine in cancer, but this raises the concerns about patient privacy and data security.

To reduce the financial burden, economic models and frameworks were studied to establish precision medicine into clinical practice. Cost effective analysis is an economic method, which can be used to evaluate the potential challenge of precision medicine interventions. Gavan et al. exemplified four studied cases, commented the cost of research and development should be considered at the early stage, while the later stage of clinical and economic evidence could practice in an iterative process. Base on the value of information of both, it can be utilized as a prioritized program for further research to reduce the uncertainly by decision-makers [444]. Notably, public sector tends to bear most of the drugs cost in the single payer health systems, Lu et al. postulated a government-industry collaborative engagement model, by integrating clinical trials into the standard of care. This allows more participants access to early stage of drug development, which able to benefit to health system-industry collaboration bodies, in terms of the value of information sharing ethically, enhance the efficiency of biomarker-dependent drug development as well as reduce the cost [445].

Other than that, future research could focus on discovery of distinct genetic markers (novel molecular aberrations) or molecular patterns that forecast therapeutic target, treatment responses and outcomes. Study on investigating combination therapies that simultaneously targeting numerous of aberrant pathways is anticipated. It is also important to discovering the advance technologies such as single-cell sequencing, liquid biopsies, advanced imaging approaches and pre-clinical models such as individualized pharmacokinetics, organoids, and the patient derived-xenograft (PDX) or -spheroid model in NPC. These avenues have the potential to shed light on the disease's heterogeneity and progression, a crucial step towards implementing precision medicine in NPC. Continued investigation into the complex interplay among the TME, immune response, and molecular abnormalities is vital for the development of novel approaches to precision immunotherapy in treating NPC. With the development of biomarker-based diagnostic and prognostic tools, personalize medicine that tailored individual NPC patient’s characteristic can be developed.

## Data Availability

Not applicable.

## Abbreviations

ACMG: American College of Medical Genetics and Genomics
AFAP1-AS1: Actin fiber-associated protein 1-antisense RNA1
Akt: Serine/threonine kinase 2
ALT: Alanine transaminase
ANCR: Antidifferentiation non-coding RNA
ANRIL: Antisense noncoding RNA in the INK4 locus
AP-1: Activator protein 1
ART: Adaptive radiotherapy
ASCO: American Society of Clinical Oncology
ASR: Age-standardised rate
AST: Aspartate transaminase
AUC: Area under the curve
BARF1: Epstein–Barr virus BamHI-A rightward frame 1
BARTs: BamHI-A rightwards transcripts
BCL-2: B-cell lymphoma 2
BLU: MYNDtype containing 10
BRAF1: V-raf murine sarcoma viral oncogene homolog B1
BST2: Bone marrow stromal cell antigen 2
CAP: College of American Pathologists
Capn4: Calpain small subunit 1
CASC2: Cancer susceptibility 2
CCND1: Cyclin D1
CCNE2: Cyclin E2
CDK2: Cyclin dependent kinase 2
CASC9: Cancer susceptibility candidate 9
Casp-12: Caspase-12
CBP: CREB binding protein
CBR: Clinical benefit rate
Cby: Chibby
CCRT: Concurrent chemoradiotherapy
cdc2: Cell division control protein 2
CDKN2A: Cyclin-dependent kinase inhibitor 2A
CEBPA: CCAAT enhancer binding protein alpha
CHFR: Checkpoint with forkhead and ring finger
COL1A: Collagen type I alpha 1
COX-2: Cyclooxygenase-2
CpG: Cytosine–phosphate–guanine
CSC: Cancer stem cell
CT: Computed tomography
CTAR: Carboxy-terminal activating region
ctDNA: Circulating tumor DNA
CTLA4: Cytotoxic T-lymphocyte protein 4
CTLs: Cytotoxic T lymphocytes
CYLD: Cylindromatosis lysine 63 deubiquitinase
CYP2E1: Cytochrome P450 family 2 subfamily E member 1
DACT2: Dishevelled binding antagonist of beta catenin 2
DAPK1: Death-associated protein kinase 1
DFS: Disease free survival
DKK1: Dickkopf WNT signalling pathway inhibitor 1
DLC-1: Deleted in liver cancer-1
DLEC1: Deleted in lung and esophageal cancer protein 1
DMFS: Distant metastasis-free survival
DNMT1: DNA methyltransferase 1
DNTTIP1: Deoxynucleotidyltransferase terminal-interacting protein 1
DOR: Duration of response
DRAIC: Downregulated RNA in cancer
DUSP2: Dual specificity phosphatase 2
EA: Early antigen
EA-IgA: Early antigen to IgA
EBERs: EBV-encoded small RNAs
EBNA: Nuclear antigen
EBNA1: Epstein Barr nuclear antigen 1
EBV: Epstein Barr Virus
ECOG: Eastern Cooperative Oncology Group
EGCG: Epigallocatechin-3-gallate
EGFR: Epidermal growth factor receptor
EMT: Epithelial to mesenchymal transition
ERBB3: Erb-B2 receptor tyrosine kinase 3
ERK: Extracellular signal-regulated kinase
EpCAM: Epithelial cell adhesion molecule
FAK: Focal adhesion kinase
FAM225B: Family with sequence similarity 225 member B
FGF2: Fibroblast growth factor 2
Flot-2: Flotillin 2
FNAC: Fine needle aspiration cytology
FOXD3-AS1: Forkhead box protein D3-antisense RNA 1
FOXO3a: Forkhead box O3a
FOXP3: Forkhead box P3
FOXQ: Forkhead box protein Q1
GAP: GTPase-activating protein
Gal-9: Galectin-9
GLOBOCAN: Global Cancer Observatory
GM-CSF: Granulocyte-macrophage colony-stimulating factor
GSK3β: Glycogen synthase kinase 3β
GyE: Gray equivalent
HDAC: Histone deacetylation
HIF-1α: Hypoxia-inducible factor 1-alpha
HI-TOPK-032: T-LAK-cell-originated protein kinase (TOPK) inhibitor
HIV: Human immunodeficiency virus
HLA: Human leukocyte antigen
HLFAFab-MCC: Human antibody Fab conjugated with mitomycin C
hTERT: Telomerase reverse transcriptase
HATs: Histone acetyl transferases
H3K9me3: Histone 3 lysine 9 trimethylation
IARC: International Agency for Research on Cancer
ICAM: Intercellular adhesion molecule 1
IFN: Interferon
ITGB3: Integrin β3
IGRT: Image-guided radiotherapy
IMCT: Intensity-modulated carbon ion therapy
IRF-3: Interferon regulatory factor 3
IKK: IκB kinase
IL: Interleuki
IMPT: Intensity-modulated proton therapy
IMRT: Intensity-modulated radiotherapy
IncRNA: Long non-coding RNA
IFNγ: Interferon-gamma
IP-10: Interferon gamma-induced protein 10
IQR: Interquartile range
ITGB3: Invasion via overexpression of integrin β3
ivm: Ivermectin
JAG1: Jagged1
JAK: Janus kinase
JAM-A: Junctional adhesion molecule-A
JNK/c-JUN: C-Jun N-terminal protein kinase
KPS: Karnofsky performance status
LAG-3: Lymphocyte activation gene 3
LARS2: Leucyl-TRNA synthetase 2, mitochondrial
LCL: Lymphoblastoid cell line
LFA: Lymphocyte function associated antigen
Lgr5: Leucine-rich repeat-containing G protein-coupled receptor 5
LHX2: LIM Homeobox 2
LINC00460: Long intergenic non-protein coding RNA 460
LMP1: Latent membrane protein 1
LRFS: Locoregional failure free-survival
LTF: Lactotransferrin
MALAT1: Metastasis associated lung adenocarcinoma transcript 1
MAPK: Mitogen-activated protein kinase
MAP2K6: Mitogen-activated protein kinase 6
MDK: Midkine
MDM2: E3 ubiquitin-protein ligase murine double minute 2
MEG3: Maternally expressed gene 3
MEK: Mitogen-activated protein kinase
MET: Mesenchymal epithelial transition
MHC: Major histocompatibility complex
MICB: MHC class I polypeptide-related sequence B
MIP-1: Macrophage inflammatory protein-1 beta
MIP-3α: Macrophage inflammatory protein-3 alpha
miRNA: MicroRNA
MK2: Mitogen-activated protein kinase-activated protein kinase 2
MLH1: MutL Homolog 1
MMP-9: Matrix metalloproteinase 9
MNK1: Mitogen activated protein kinase interacting kinases
MPL: Monophosphoryl lipid A
MRI: Magnetic resonance imaging
MTA1: Metastatic tumor antigen 1
MTB: Molecular tumor board
mTOR: Mammalian target of rapamycin
mTORC1: Mammalian target of rapamycin complex 1
MVA: Modified Vaccinia Ankara
MVA-EL: Inactivate fusion of EBNA1 and LMP2 with MVA
NCCN: National Comprehensive Cancer Network
NDV: Newcastle disease virus
NEAT1: Nuclear enriched abundant transcript 1
NESG1: Nasopharyngeal epithelium specific protein 1
NF-κB: Nuclear factor kappa B
NFKBIA: NF-κB inhibitor alpha
NF-κB p65: Nuclear factor kappa-light-chain-enhancer of activated B cells p65
NF1: Neurofibromatosis 1
NGS: Next-generation genome sequencing
NK: Natural killer
NKILA: NF-kappabeta-interacting long noncoding RNA
NLRC5: NOD-like receptor family CARD domain containing 5
NPC: Nasopharyngeal carcinoma
NRF1: Inhibiting nuclear respiratory factor 1
NSCLC: Non-small cell lung carcinoma
ORR: Objective response rate
OS: Overall survival
PAK1: P21 (RAC1) activated kinase 1
PBMC: Peripheral blood mononuclear cell
PCDH17: Protocadherin 17
PDGFR: Platelet-derived growth factor receptor
PD-1: Programmed cell death protein 1
PD-L1: Programmed death-ligand 1
PDX: Patient derived-xenograft
PET–CT: Positron emission tomography–computed tomography
PFS: Progression-free survival
PIK3CA: Phosphatidylinositol-4,5-bisphosphate 3-kinase catalytic subunit alpha
PIK3R1: Phosphoinositide-3-kinase regulatory subunit 1
Pim1: Provirus integration site for moloney murine leukemia virus 1
PinX1: Pin2 telomeric repeat factor 1-interacting telomerase inhibitor 1
PIN 1: Peptidyl-prolyl *cis*–*trans* isomerase NIMA-interacting 1
PI3K: Phosphoinositide 3-Kinase
POLN: Polymerase nu
PPV: Positive predictive value
PTEN: Phosphatase and tensin homolog
PTLD: Posttransplant lymphoproliferative disease
PTV: Planning target volume
PVT1: Plasmacytoma variant translocation 1
PY*LKTK: Phosphopeptide inhibitor
p21: Cyclin-dependent kinase inhibitory protein-1
Ras: Rat sarcoma virus
RASSF1A: Ras association domain family member 1
RBBP8: Retinoblastoma binding protein 8
RBM3: RNA-binding motif protein 3
RERG: Ras-like estrogen-regulated growth inhibitor
RIG-I: Retinoic acid-inducible gene I
RKIP: Raf kinase inhibitory protein
Rsf: Remodelling and spacing factor 1
SATB: Special AT-rich sequence-binding protein-1
SBRT: Stereotactic body radiation therapy
SFRP1: Secreted frizzled related protein 1
SIRT6: Sirtuin 6
Skp2: S-phase kinase-associated protein 2
slug: Zinc finger protein SNAI2
SNP: Single nucleotide polymorphism
SREBP1: Sterol regulatory element-binding transcription factor 1
SRY-Box: Sex-determining region Y protein-Box
STAT3: Signal transducer and activator of transcription 3
TAP: Transporter associated with antigen processing
TAP2: Antigen peptide transporter 2
TET1: Ten–eleven translocation methylcytosine dioxygenase 1
TGF-β: Transforming growth factor beta
Th1: T-helper-type 1
TIGAR: TP53 inducible glycolysis and apoptosis regulator
TILs: Tumor infiltrating leukocytes
TIM-3: T cell immunoglobulin and mucin domain-containing protein 3
TKIs: Tyrosine kinase inhibitors
TME: Tumor microenvironment
TNF: Tumor necrosis factor
TNFAIP3: Tumor necrosis factor-alpha-induced protein 3
TNM: Tumor, node, and metastasis
Tp53: Tumor protein P53
TP73-AS1: P73 antisense RNA 1T
TRAF3: Tumor necrosis factor receptor-associated factor 3
TRAIL: TNF-related apoptosis ligand
Treg: Regulatory T-cells
TSG: Tumor suppressor gene
UCA1: Urothelial cancer associated 1
VCA: Viral capsid antigen
VCA-IgA: IgA to EBV capsid antigen
VEGF: Vascular endothelial growth factor
VEGFR: Vascular endothelial growth factor receptor
VLPs: Virus-like particles
VM: Vasculogenic mimicry
VMAT: Volumetric modulated arc therapy
VPS33B: Vacuolar protein sorting 33B
WBC: White blood cell
WHO: World Health Organisation
WIF1: Wnt inhibitory factor 1
Wnt: Wingless-related integration site
ZMYND10: Zinc finger MYND-type containing 10
3′-UTR: 3′-Untranslated regions
5-FU: 5-Fluorouracil

## References

[1] Chan KCA, Woo JKS, King A (2017). Analysis of plasma Epstein–Barr virus DNA to screen for nasopharyngeal cancer. N Engl J Med.

[2] Zhu Q, Zhao G, Li Y (2021). Advances in pathogenesis and precision medicine for nasopharyngeal carcinoma. MedComm.

[3] Young LS (2020). A novel Epstein–Barr virus subtype associated with nasopharyngeal carcinoma found in South China. Cancer Commun.

[4] Ho CS (2017). Beating ‘guangdong cancer’: a review and update on nasopharyngeal cancer. Hong Kong Med J.

[5] Gibb AG, van Hasselt CA (1999). Nasopharyngeal carcinoma.

[6] GLOBOCAN. Estimated number of incident cases nasopharynx, both sexes, all ages. https://gco.iarc.fr/.

[7] Tu C, Zeng Z, Qi P (2018). Identification of genomic alterations in nasopharyngeal carcinoma and nasopharyngeal carcinoma-derived Epstein–Barr virus by whole-genome sequencing. Carcinogenesis.

[8] Stelow EB, Wenig BM (2017). Update from the 4th edition of the World Health Organization classification of head and neck tumours: nasopharynx. Head Neck Pathol.

[9] Kang Y, He W, Qiao J (2020). Advances in targeted therapy mainly based on signal pathways for nasopharyngeal carcinoma. Signal Transduct Target Ther.

[10] Yu WM, Hussain SSM (2009). Incidence of nasopharyngeal carcinoma in Chinese immigrants, compared with Chinese in China and South East Asia: review. J Laryngol Otol.

[11] Chow YP, Tan LP, Chai SJ (2017). Exome sequencing identifies potentially druggable mutations in nasopharyngeal carcinoma. Sci Rep.

[12] Zheng H, Dai W, Cheung AKL (2016). Whole-exome sequencing identifies multiple loss-of-function mutations of NF-κB pathway regulators in nasopharyngeal carcinoma. Proc Natl Acad Sci USA.

[13] Dai W, Zheng H, Cheung AKL (2016). Whole-exome sequencing identifies MST1R as a genetic susceptibility gene in nasopharyngeal carcinoma. Proc Natl Acad Sci USA.

[14] Wong EYL, Wong SCC, Ming Lok Chan C (2015). TP53-induced glycolysis and apoptosis regulator promotes proliferation and invasiveness of nasopharyngeal carcinoma cells. Oncol Lett.

[15] Lin DC, Meng X, Hazawa M (2014). The genomic landscape of nasopharyngeal carcinoma. Nat Genet.

[16] Zuo XY, Feng QS, Sun J (2019). X-chromosome association study reveals genetic susceptibility loci of nasopharyngeal carcinoma. Biol Sex Differ.

[17] Tang LL, Chen WQ, Xue WQ (2016). Global trends in incidence and mortality of nasopharyngeal carcinoma. Cancer Lett.

[18] Rietveld CA, Medland SE, Derringer J (2013). GWAS of 126,559 individuals identifies genetic variants associated with educational attainment. Science.

[19] Lu SJ, Day NE, Degos L (1990). Linkage of a nasopharyngeal carcinoma susceptibility locus to the HLA region. Nature.

[20] Lo KW, Chung GTY, To KF (2012). Deciphering the molecular genetic basis of NPC through molecular, cytogenetic, and epigenetic approaches. Semin Cancer Biol.

[21] Shih-Hsin WuL (2006). Construction of evolutionary tree models for nasopharyngeal carcinoma using comparative genomic hybridization data. Cancer Genet Cytogenet.

[22] Xiao RW, Wang F, Wang TM (2022). Rare POLN mutations confer risk for familial nasopharyngeal carcinoma through weakened Epstein–Barr virus lytic replication. EBioMedicine.

[23] Yarza R, Bover M, Agulló-Ortuño MT (2021). Current approach and novel perspectives in nasopharyngeal carcinoma: the role of targeting proteasome dysregulation as a molecular landmark in nasopharyngeal cancer. J Exp Clin Cancer Res.

[24] Young LS, Yap LF, Murray PG (2016). Epstein–Barr virus: more than 50 years old and still providing surprises. Nat Rev Cancer.

[25] Dai W, Zheng H, Cheung AKL (2016). Genetic and epigenetic landscape of nasopharyngeal carcinoma. Chin Clin Oncol.

[26] Maas B, Ho C, Hamilton S (2018). Impact of neoadjuvant chemotherapy on the administration of concurrent chemoradiation for locally advanced nasopharyngeal carcinoma. Cureus.

[27] Okekpa SI, Mydin RBSMN, Mangantig E (2019). Nasopharyngeal carcinoma (NPC) risk factors: a systematic review and meta-analysis of the association with lifestyle, diets, socioeconomic and sociodemographic in Asian region. Asian Pac J Cancer Prev.

[28] Roy Chattopadhyay N, Das P, Chatterjee K (2017). Higher incidence of nasopharyngeal carcinoma in some regions in the world confers for interplay between genetic factors and external stimuli. Drug Discov Ther.

[29] Yong SK, Ha TC, Yeo MCR (2017). Associations of lifestyle and diet with the risk of nasopharyngeal carcinoma in Singapore: a case–control study. Chin J Cancer.

[30] Lo KW, To KF, Huang DP (2004). Focus on nasopharyngeal carcinoma. Cancer Cell.

[31] Farhat F, Daulay ER, Chrestella J (2020). The association of CYP2E1 polymorphism and environmental factor in nasopharyngeal carcinoma patients. Open Access Maced J Med Sci.

[32] Tang LQ, Zhang H, Li Y, et al. A sponsored supplement to science: precision medicine in China, Chapter 2, Precision medicine for nasopharyngeal carcinoma, Science, 2016; 24–27

[33] Chua DTT, Sham JST, Kwong DLW (2003). Treatment outcome after radiotherapy alone for patients with stage I–II nasopharyngeal carcinoma. Cancer.

[34] Zhang L, Yang Y, Chen X (2020). 912MO A single-arm, open-label, multicenter phase II study of camrelizumab in patients with recurrent or metastatic (R/M) nasopharyngeal carcinoma (NPC) who had progressed on ≥2 lines of chemotherapy: CAPTAIN study. Ann Oncol.

[35] Liu ZG, Zhao Y, Tang J (2016). Nimotuzumab combined with concurrent chemoradiotherapy in locally advanced nasopharyngeal carcinoma: a retrospective analysis. Oncotarget.

[36] Liu T, Sun Q, Li Q (2015). Dual PI3K/mTOR inhibitors, GSK2126458 and PKI-587, suppress tumor progression and increase radiosensitivity in nasopharyngeal carcinoma. Mol Cancer Ther.

[37] Huang Y, Zhang L, Pan J (2013). A phase II, multicenter, open-label, single-arm trial of famitinib in patients with advanced recurrent and/or metastatic nasopharyngeal carcinoma (NPC) after two previous treatment regimens. J Clin Oncol.

[38] Jiang H, Fan D, Zhou G (2010). Phosphatidylinositol 3-kinase inhibitor (LY294002) induces apoptosis of human nasopharyngeal carcinoma in vitro and in vivo. J Exp Clin Cancer Res.

[39] Jin YY, Yang WZ, Zou S (2020). Chemoradiotherapy combined with NK cell transfer in a patient with recurrent and metastatic nasopharyngeal carcinoma inducing long-term tumor control: a case report. Medicine.

[40] Chia WK, Teo M, Wang WW (2014). Adoptive T-cell transfer and chemotherapy in the first-line treatment of metastatic and/or locally recurrent nasopharyngeal carcinoma. Mol Ther.

[41] Simo R, Robinson M, Lei M (2016). Nasopharyngeal carcinoma: United Kingdom National Multidisciplinary guidelines. J Laryngol Otol.

[42] Bossi P, Chan AT, Licitra L (2021). Nasopharyngeal carcinoma: ESMO-EURACAN clinical practice guidelines for diagnosis, treatment and follow-up. Ann Oncol Off J Eur Soc Med Oncol.

[43] Wang KH, Austin SA, Chen SH (2017). Nasopharyngeal carcinoma diagnostic challenge in a nonendemic setting: our experience with 101 patients. Perm J.

[44] King AD, Vlantis AC, Bhatia KSS (2011). Primary nasopharyngeal carcinoma: diagnostic accuracy of MR imaging versus that of endoscopy and endoscopic biopsy. Radiology.

[45] Gorolay VV, Niles NN, Huo YR (2022). MRI detection of suspected nasopharyngeal carcinoma: a systematic review and meta-analysis. Neuroradiology.

[46] Yang X, Wu J, Chen X (2023). Application of artificial intelligence to the diagnosis and therapy of nasopharyngeal carcinoma. J Clin Med.

[47] Basu K, Sinha R, Ong A (2020). Artificial intelligence: how is it changing medical sciences and its future?. Indian J Dermatol.

[48] Rueda Domínguez A, Cirauqui B, García Castaño A (2022). SEOM-TTCC clinical guideline in nasopharynx cancer (2021). Clin Transl Oncol.

[49] American Cancer Society. Nasopharyngeal cancer early detection, diagnosis, and staging. 2022. https://www.cancer.org/cancer/types/nasopharyngeal-cancer/detection-diagnosis-staging.html

[50] Obel N, Høier-Madsen M, Kangro H (1996). Serological and clinical findings in patients with serological evidence of reactivated Epstein–Barr virus infection. APMIS.

[51] Zeng Y, Jan MG, Zhang Q (1982). Serological mass survey for early detection of nasopharyngeal carcinoma in Wuzhou City, China. Int J Cancer.

[52] Li T, Guo X, Ji M (2018). Establishment and validation of a two-step screening scheme for improved performance of serological screening of nasopharyngeal carcinoma. Cancer Med.

[53] Chien Y-C, Chen J-Y, Liu M-Y (2001). Serologic markers of Epstein–Barr virus infection and nasopharyngeal carcinoma in Taiwanese men. N Engl J Med.

[54] Henle G, Henle W (1976). Epstein–Barr virus-specific IgA serum antibodies as an outstanding feature of nasopharyngeal carcinoma. Int J Cancer.

[55] Li YQ, Khin NS, Chua MLK (2018). The evolution of Epstein–Barr virus detection in nasopharyngeal carcinoma. Cancer Biol Med.

[56] Fachiroh J, Paramita DK, Hariwiyanto B (2006). Single-assay combination of Epstein–Barr Virus (EBV) EBNA1- and viral capsid antigen-p18-derived synthetic peptides for measuring anti-EBV immunoglobulin G (IgG) and IgA antibody levels in sera from nasopharyngeal carcinoma patients: options for field screening. J Clin Microbiol.

[57] Zhen Y, Fang W, Zhao M (2017). miR-374a-CCND1-pPI3K/AKT-c-JUN feedback loop modulated by PDCD4 suppresses cell growth, metastasis, and sensitizes nasopharyngeal carcinoma to cisplatin. Oncogene.

[58] Chen Y, Zhao W, Lin L (2015). Nasopharyngeal Epstein–Barr virus load: an efficient supplementary method for population-based nasopharyngeal carcinoma screening. PLoS ONE.

[59] Leung SF, Tam JS, Chan ATC (2004). Improved accuracy of detection of nasopharyngeal carcinoma by combined application of circulating Epstein–Barr virus DNA and anti-Epstein–Barr viral capsid antigen IgA antibody. Clin Chem.

[60] Tsao SW, Tsang CM, Lo KW (2017). Epstein–Barr virus infection and nasopharyngeal carcinoma. Philos Trans R Soc B Biol Sci.

[61] Kimura H, Ito Y, Suzuki R (2008). Measuring Epstein–Barr virus (EBV) load: the significance and application for each EBV-associated disease. Rev Med Virol.

[62] Song C, Yang S (2013). A meta-analysis on the EBV DNA and VCA-IgA in diagnosis of nasopharyngeal carcinoma. Pak J Med Sci.

[63] Liu Y, Fang Z, Liu L (2011). Detection of Epstein–Barr virus DNA in serum or plasma for nasopharyngeal cancer: a meta-analysis. Genet Test Mol Biomark.

[64] Lam WKJ, Jiang P, Chan KCA (2018). Sequencing-based counting and size profiling of plasma Epstein–Barr virus DNA enhance population screening of nasopharyngeal carcinoma. Proc Natl Acad Sci USA.

[65] Siak PY, Khoo ASB, Leong CO (2021). Current status and future perspectives about molecular biomarkers of nasopharyngeal carcinoma. Cancers.

[66] He Y, Li J, Mao W (2018). HLA common and well-documented alleles in China. HLA.

[67] Chin YM, Mushiroda T, Takahashi A (2015). HLA-A SNPs and amino acid variants are associated with nasopharyngeal carcinoma in Malaysian Chinese. Int J Cancer.

[68] Hsu WL, Tse KP, Liang S (2012). Evaluation of human leukocyte antigen-A (HLA-A), other non-HLA markers on chromosome 6p21 and risk of nasopharyngeal carcinoma. PLoS ONE.

[69] Ye M, Huang T, Ni C (2017). Diagnostic capacity of RASSF1A promoter methylation as a biomarker in tissue, brushing, and blood samples of nasopharyngeal carcinoma. EBioMedicine.

[70] Bruce JP, To KF, Lui VWY (2021). Whole-genome profiling of nasopharyngeal carcinoma reveals viral-host co-operation in inflammatory NF-κB activation and immune escape. Nat Commun.

[71] Lee AWM, Sze WM, Au JSK (2005). Treatment results for nasopharyngeal carcinoma in the modern era: the Hong Kong experience. Int J Radiat Oncol Biol Phys.

[72] Rottey S, Madani I, Deron P (2011). Modern treatment for nasopharyngeal carcinoma: current status and prospects. Curr Opin Oncol.

[73] Chen C, Zhou Y, Zhang X (2020). Anti-epidermal growth factor receptor monoclonal antibody plus palliative chemotherapy as a first-line treatment for recurrent or metastatic nasopharyngeal carcinoma. Cancer Med.

[74] Lee AW, Ng WT, Pan JJ (2019). International guideline on dose prioritization and acceptance criteria in radiation therapy planning for nasopharyngeal carcinoma. Int J Radiat Oncol Biol Phys.

[75] Perri F, Della Vittoria Scarpati G, Caponigro F (2019). Management of recurrent nasopharyngeal carcinoma: current perspectives. Onco Targets Ther.

[76] Chen W, Hu GH (2015). Biomarkers for enhancing the radiosensitivity of nasopharyngeal carcinoma. Cancer Biol Med.

[77] Tham IWK, Lu JJ (2012). Treatment of early stage nasopharyngeal carcinoma: conventional versus new radiation therapy technologies. J Radiat Oncol.

[78] Zhu G-L, Xu C, Ma J (2020). Combination of precision radiotherapy with chemotherapy and immunotherapy in non-recurrent/metastatic nasopharyngeal carcinoma. Ann Nasopharynx Cancer.

[79] Long G, Tang W, Fu X (2019). Pre-treatment serum lactate dehydrogenase predicts distant metastasis and poor survival in nasopharyngeal carcinoma. J Cancer.

[80] Leong YH, Soon YY, Lee KM (2018). Long-term outcomes after reirradiation in nasopharyngeal carcinoma with intensity-modulated radiotherapy: a meta-analysis. Head Neck.

[81] Ma R, Zhao LN, Yang H (2018). RNA binding motif protein 3 (RBM3) drives radioresistance in nasopharyngeal carcinoma by reducing apoptosis via the PI3K/AKT/Bcl-2 signaling pathway. Am J Transl Res.

[82] Li LN, Xiao T, Yi HM (2017). MiR-125b increases nasopharyngeal carcinoma radioresistance by targeting A20/NF-κB signaling pathway. Mol Cancer Ther.

[83] Huang J, Zou Q, Qian D (2017). Intensity-modulated radiotherapy plus nimotuzumab with or without concurrent chemotherapy for patients with locally advanced nasopharyngeal carcinoma. Onco Targets Ther.

[84] Lee AWM, Ma BBY, Ng WT (2015). Management of nasopharyngeal carcinoma: current practice and future perspective. J Clin Oncol.

[85] Jin Y, Cai XY, Shi YX (2012). Comparison of five cisplatin-based regimens frequently used as the first-line protocols in metastatic nasopharyngeal carcinoma. J Cancer Res Clin Oncol.

[86] Le QT, Colevas AD, O’Sullivan B (2019). Current treatment landscape of nasopharyngeal carcinoma and potential trials evaluating the value of immunotherapy. J Natl Cancer Inst.

[87] Wong TS, Gao W, Chan JYW (2015). Biomarkers in nasopharyngeal carcinoma and ionizing radiation. Biomarkers in cancer, biomarkers in disease: methods, discoveries and applications.

[88] Sun Y, Li WF, Chen NY (2016). Induction chemotherapy plus concurrent chemoradiotherapy versus concurrent chemoradiotherapy alone in locoregionally advanced nasopharyngeal carcinoma: a phase 3, multicentre, randomised controlled trial. Lancet Oncol.

[89] Cao SM, Yang Q, Guo L (2017). Neoadjuvant chemotherapy followed by concurrent chemoradiotherapy versus concurrent chemoradiotherapy alone in locoregionally advanced nasopharyngeal carcinoma: a phase III multicentre randomised controlled trial. Eur J Cancer.

[90] Chen L, Hu CS, Chen XZ (2017). Adjuvant chemotherapy in patients with locoregionally advanced nasopharyngeal carcinoma: long-term results of a phase 3 multicentre randomised controlled trial. Eur J Cancer.

[91] Hui EP, Ma BBY, Chan KCA (2015). Clinical utility of plasma Epstein–Barr virus DNA and ERCC1 single nucleotide polymorphism in nasopharyngeal carcinoma. Cancer.

[92] Liang ZG, Chen XQ, Lin GX (2017). Significant survival benefit of adjuvant chemotherapy after concurrent chemoradiotherapy in locally advanced high-risk nasopharyngeal carcinoma. Sci Rep.

[93] Zhang L, Huang Y, Hong S (2016). Gemcitabine plus cisplatin versus fluorouracil plus cisplatin in recurrent or metastatic nasopharyngeal carcinoma: a multicentre, randomised, open-label, phase 3 trial. Lancet.

[94] Jin T, Qin WF, Jiang F (2019). Cisplatin and fluorouracil induction chemotherapy with or without docetaxel in locoregionally advanced nasopharyngeal carcinoma. Transl Oncol.

[95] Ding RB, Chen P, Rajendran BK (2021). Molecular landscape and subtype-specific therapeutic response of nasopharyngeal carcinoma revealed by integrative pharmacogenomics. Nat Commun.

[96] Liu J, Yu H, Sun X (2017). Salvage endoscopic nasopharyngectomy for local recurrent or residual nasopharyngeal carcinoma: a 10-year experience. Int J Clin Oncol.

[97] Na’ara S, Amit M, Billan S (2014). Outcome of patients undergoing salvage surgery for recurrent nasopharyngeal carcinoma: a meta-analysis. Ann Surg Oncol.

[98] Liu YP, Lv X, Zou X (2019). Minimally invasive surgery alone compared with intensity-modulated radiotherapy for primary stage I nasopharyngeal carcinoma. Cancer Commun.

[99] Vlantis AC, Lee DLY, Wong EWY (2017). Endoscopic nasopharyngectomy in recurrent nasopharyngeal carcinoma: a case series, literature review, and pooled analysis. Int Forum Allergy Rhinol.

[100] Argirion I, Zarins KR, Ruterbusch JJ (2020). Increasing incidence of Epstein–Barr virus-related nasopharyngeal carcinoma in the United States. Cancer.

[101] Van Zyl DG, Mautner J, Delecluse HJ (2019). Progress in EBV vaccines. Front Oncol.

[102] Bar-Sela G, Kuten A, Minkov I (2004). Prevalence and relevance of EBV latency in nasopharyngeal carcinoma in Israel. J Clin Pathol.

[103] Johannsen E, Luftig M, Chase MR (2004). Proteins of purified Epstein–Barr virus. Proc Natl Acad Sci USA.

[104] Sokal EM, Hoppenbrouwers K, Vandermeulen C (2007). Recombinant gp350 vaccine for infectious mononucleosis: a phase 2, randomized, double-blind, placebo-controlled trial to evaluate the safety, immunogenicity, and efficacy of an Epstein–Barr virus vaccine in healthy young adults. J Infect Dis.

[105] Rees L, Tizard EJ, Morgan AJ (2009). A phase I trial of Epstein–Barr virus gp350 vaccine for children with chronic kidney disease awaiting transplantation. Transplantation.

[106] Cui X, Cao Z, Chen Q (2016). Rabbits immunized with Epstein–Barr virus gH/gL or gB recombinant proteins elicit higher serum virus neutralizing activity than gp350. Vaccine.

[107] Ogembo GG, Muraswki MR, McGinnes LW (2015). A chimeric EBV gp350/220-based VLP replicates the virion B-cell attachment mechanism and elicits long-lasting neutralizing antibodies in mice. J Transl Med.

[108] Delecluse HJ, Pich D, Hilsendegen T (1999). A first-generation packaging cell line for Epstein–Barr virus-derived vectors. Proc Natl Acad Sci USA.

[109] Ruiss R, Jochum S, Wanner G (2011). A virus-like particle-based Epstein–Barr virus vaccine. J Virol.

[110] Elliott SL, Suhrbier A, Miles JJ (2008). Phase I trial of a CD8+ T-cell peptide epitope-based vaccine for infectious mononucleosis. J Virol.

[111] Bu W, Joyce MG, Nguyen H (2019). Immunization with components of the viral fusion apparatus elicits antibodies that neutralize Epstein–Barr virus in B cells and epithelial cells. Immunity.

[112] Spear PG, Longnecker R (2003). Herpesvirus entry: an update. J Virol.

[113] Dolgin E (2021). The tangled history of mRNA vaccines. Nature.

[114] Moderna. Moderna announces first participant dosed in phase 1 study of its mRNA Epstein–Barr virus (EBV) vaccine. AccessWire. 2022. https://www.accesswire.com/681034/Moderna-Announces-First-Participant-Dosed-in-Phase-1-Study-of-its-mRNA-Epstein-Barr-Virus-EBV-Vaccine.

[115] Roberti A, Valdes AF, Torrecillas R (2019). Epigenetics in cancer therapy and nanomedicine. Clin Epigenet.

[116] Xu Y, Huang X, Ye W (2020). Comprehensive analysis of key genes associated with ceRNA networks in nasopharyngeal carcinoma based on bioinformatics analysis. Cancer Cell Int.

[117] Dai W, Dong P, Liu J (2019). Euscaphic acid inhibits proliferation and promotes apoptosis of nasopharyngeal carcinoma cells by silencing the PI3K/AKT/mTOR signaling pathway. Am J Transl Res.

[118] Li Y, Liu X, Lin X (2019). Chemical compound cinobufotalin potently induces FOXO1-stimulated cisplatin sensitivity by antagonizing its binding partner MYH9. Signal Transduct Target Ther.

[119] He Q, Ren X, Chen J (2016). miR-16 targets fibroblast growth factor 2 to inhibit NPC cell proliferation and invasion via PI3K/AKT and MAPK signaling pathways. Oncotarget.

[120] Liu J, Huang W, Ren C (2015). Flotillin-2 promotes metastasis of nasopharyngeal carcinoma by activating NF-κB and PI3K/Akt3 signaling pathways. Sci Rep.

[121] Li Y, He Q, Wen X (2019). EZH2-DNMT1-mediated epigenetic silencing of miR-142-3p promotes metastasis through targeting ZEB2 in nasopharyngeal carcinoma. Cell Death Differ.

[122] Lo AKF, Lung RWM, Dawson CW (2018). Activation of sterol regulatory element-binding protein 1 (SREBP1)-mediated lipogenesis by the Epstein–Barr virus-encoded latent membrane protein 1 (LMP1) promotes cell proliferation and progression of nasopharyngeal carcinoma. J Pathol.

[123] Wang MH, Sun R, Zhou XM (2018). Epithelial cell adhesion molecule overexpression regulates epithelial–mesenchymal transition, stemness and metastasis of nasopharyngeal carcinoma cells via the PTEN/AKT/mTOR pathway. Cell Death Dis.

[124] Xiang T, Lin YX, Ma W (2018). Vasculogenic mimicry formation in EBV-associated epithelial malignancies. Nat Commun.

[125] Guo Y, Zhai J, Zhang J (2019). Improved radiotherapy sensitivity of nasopharyngeal carcinoma cells by miR-29-3p targeting COL1A1 3′-UTR. Med Sci Monit.

[126] Xu S, Li Y, Lu Y (2018). LZTS2 inhibits PI3K/AKT activation and radioresistance in nasopharyngeal carcinoma by interacting with p85. Cancer Lett.

[127] Ma BBY, Goh BC, Lim WT (2015). Multicenter phase II study of the AKT inhibitor MK-2206 in recurrent or metastatic nasopharyngeal carcinoma from patients in the mayo phase II consortium and the cancer therapeutics research group (MC1079). Invest New Drugs.

[128] Liang Z, Liu Z, Cheng C (2019). VPS33B interacts with NESG1 to modulate EGFR/PI3K/AKT/c-Myc/P53/miR-133a-3p signaling and induce 5-fluorouracil sensitivity in nasopharyngeal carcinoma. Cell Death Dis.

[129] Lu J, Liu QH, Wang F (2018). Exosomal miR-9 inhibits angiogenesis by targeting MDK and regulating PDK/AKT pathway in nasopharyngeal carcinoma. J Exp Clin Cancer Res.

[130] Fan X, Wang Y, Song J (2019). MDM2 inhibitor RG7388 potently inhibits tumors by activating p53 pathway in nasopharyngeal carcinoma. Cancer Biol Ther.

[131] Yee-Lin V, Pooi-Fong W, Soo-Beng AK (2018). Nutlin-3, A p53-Mdm2 antagonist for nasopharyngeal carcinoma treatment. Mini Rev Med Chem.

[132] Shi C, Guan Y, Zeng L (2018). High COX-2 expression contributes to a poor prognosis through the inhibition of chemotherapy-induced senescence in nasopharyngeal carcinoma. Int J Oncol.

[133] Yu C, Chen F, Wang X (2020). Pin2 telomeric repeat factor 1-interacting telomerase inhibitor 1 (PinX1) inhibits nasopharyngeal cancer cell stemness: implication for cancer progression and therapeutic targeting. J Exp Clin Cancer Res.

[134] Chen JJ, Liu SX, Chen MZ (2015). Has-miR-125a and 125b are induced by treatment with cisplatin in nasopharyngeal carcinoma and inhibit apoptosis in a p53-dependent manner by targeting p53 mRNA. Mol Med Rep.

[135] Zuo L, Xie Y, Tang J (2019). Targeting exosomal EBV-LMP1 transfer and miR-203 expression via the NF-κB pathway: the therapeutic role of aspirin in NPC. Mol Ther Nucleic Acids.

[136] Ding RR, Yuan JL, Jia YN (2019). Epstein–Barr virus-encoded LMP1 regulated Pim1 kinase expression promotes nasopharyngeal carcinoma cells proliferation. Onco Targets Ther.

[137] Wang W, Cheng H, Gu X (2019). The natural flavonoid glycoside vitexin displays preclinical antitumor activity by suppressing NF-κB signaling in nasopharyngeal carcinoma. Onco Targets Ther.

[138] Peng T, Hu M, Wu TT (2015). Andrographolide suppresses proliferation of nasopharyngeal carcinoma cells via attenuating NF-κB pathway. Biomed Res Int.

[139] Sun FR, Wang SL, Wang M (2020). Simvastatin induces apoptosis of nasopharyngeal carcinoma cells through NF-κB signaling pathway. Eur Rev Med Pharmacol Sci.

[140] Kuang CM, Fu X, Hua YJ (2017). BST2 confers cisplatin resistance via NF-κB signaling in nasopharyngeal cancer. Cell Death Dis.

[141] Ouyang L, Yi L, Li J (2018). SIRT6 overexpression induces apoptosis of nasopharyngeal carcinoma by inhibiting NF-κB signaling. Onco Targets Ther.

[142] Cheng N, Guo Y (2017). Long noncoding RNA NEAT1 promotes nasopharyngeal carcinoma progression through regulation of miR-124/NF-κB pathway. Onco Targets Ther.

[143] Huang W, Liu J, Feng X (2015). DLC-1 induces mitochondrial apoptosis and epithelial mesenchymal transition arrest in nasopharyngeal carcinoma by targeting EGFR/Akt/NF-κB pathway. Med Oncol.

[144] Zhao W, Ma N, Wang S (2017). RERG suppresses cell proliferation, migration and angiogenesis through ERK/NF-κB signaling pathway in nasopharyngeal carcinoma. J Exp Clin Cancer Res.

[145] Li YJ, Wu SL, Lu SM (2015). (−)-Epigallocatechin-3-gallate inhibits nasopharyngeal cancer stem cell self-renewal and migration and reverses the epithelial–mesenchymal transition via NF-κB p65 inactivation. Tumour Biol.

[146] Xu J, Ying Y, Xiong G (2019). Amyloid β precursor protein silencing attenuates epithelial–mesenchymal transition of nasopharyngeal carcinoma cells via inhibition of the MAPK pathway. Mol Med Rep.

[147] Gallardo F, Mariamé B, Gence R (2018). Macrocyclic lactones inhibit nasopharyngeal carcinoma cells proliferation through PAK1 inhibition and reduce in vivo tumor growth. Drug Des Dev Ther.

[148] Wang MY, Lin ZR, Cao Y (2016). PDZ binding kinase (PBK) is a theranostic target for nasopharyngeal carcinoma: driving tumor growth via ROS signaling and correlating with patient survival. Oncotarget.

[149] Zhang M, Jiang L, Tao J (2019). Design, synthesis and biological evaluation of 4-aniline-thieno[2,3-d]pyrimidine derivatives as MNK1 inhibitors against renal cell carcinoma and nasopharyngeal carcinoma. Bioorg Med Chem.

[150] Liu T, Li Q, Sun Q (2014). MET inhibitor PHA-665752 suppresses the hepatocyte growth factor-induced cell proliferation and radioresistance in nasopharyngeal carcinoma cells. Biochem Biophys Res Commun.

[151] Zhang X, Shao S-J, Zhou J-H (2018). Tumor suppressor BLU exerts growth inhibition by blocking ERK signaling and disrupting cell cycle progression through RAS pathway interference. Int J Clin Exp Pathol.

[152] Du M, Chen W, Zhang W (2018). TGF-β regulates the ERK/MAPK pathway independent of the SMAD pathway by repressing miRNA-124 to increase MALAT1 expression in nasopharyngeal carcinoma. Biomed Pharmacother.

[153] Tian Y, Yan M, Zheng J (2019). miR-483-5p decreases the radiosensitivity of nasopharyngeal carcinoma cells by targeting DAPK1. Lab Invest.

[154] Morris MA, Laverick L, Wei W (2018). The EBV-encoded oncoprotein, LMP1, induces an epithelial-to-mesenchymal transition (EMT) via its CTAR1 domain through integrin-mediated ERK-MAPK signalling. Cancers.

[155] Xu M, Cheung CCM, Chow C (2016). Overexpression of PIN1 enhances cancer growth and aggressiveness with cyclin D1 induction in EBV-associated nasopharyngeal carcinoma. PLoS ONE.

[156] Liu SC, Huang CM, Bamodu OA (2019). Ovatodiolide suppresses nasopharyngeal cancer by targeting stem cell-like population, inducing apoptosis, inhibiting EMT and dysregulating JAK/STAT signaling pathway. Phytomedicine.

[157] He QY, Yi HM, Yi H (2015). Reduction of RKIP expression promotes nasopharyngeal carcinoma invasion and metastasis by activating Stat3 signaling. Oncotarget.

[158] Gao J, Shao Z, Yan M (2018). Targeted regulation of STAT3 by miR-29a in mediating Taxol resistance of nasopharyngeal carcinoma cell line CNE-1. Cancer Biomark.

[159] Liu C, Zhang H, Liu H (2019). Long noncoding RNA UCA1 accelerates nasopharyngeal carcinoma cell progression by modulating miR-124-3p/ITGB1 axis. Onco Targets Ther.

[160] Li X, Zhao Z, Li M (2018). Sulforaphane promotes apoptosis, and inhibits proliferation and self-renewal of nasopharyngeal cancer cells by targeting STAT signal through miRNA-124-3p. Biomed Pharmacother.

[161] Chan KC, Chan LS, Ip JCY (2015). Therapeutic targeting of CBP/β-catenin signaling reduces cancer stem-like population and synergistically suppresses growth of EBV-positive nasopharyngeal carcinoma cells with cisplatin. Sci Rep.

[162] Wan FZ, Chen KH, Sun YC (2020). Exosomes overexpressing miR-34c inhibit malignant behavior and reverse the radioresistance of nasopharyngeal carcinoma. J Transl Med.

[163] Cai CF, Liu LM, Shangguan HJ (2018). Anti-oncogenic activity of Chibby in the development of human nasopharyngeal carcinoma. Oncol Lett.

[164] Hu H, Wang G, Lli C (2017). miR-124 suppresses proliferation and invasion of nasopharyngeal carcinoma cells through the Wnt/β-catenin signaling pathway by targeting Capn4. Onco Targets Ther.

[165] Liang TS, Zheng YJ, Wang J (2021). Retraction Note: MicroRNA-506 inhibits tumor growth and metastasis in nasopharyngeal carcinoma through the inactivation of the Wnt/β-catenin signaling pathway by downregulating LHX2. J Exp Clin Cancer Res.

[166] Luo M, Wu C, Guo E (2019). FOXO3a knockdown promotes radioresistance in nasopharyngeal carcinoma by inducing epithelial–mesenchymal transition and the Wnt/β-catenin signaling pathway. Cancer Lett.

[167] Li F, Song X, Li X (2020). Lgr5 maintains stemness and regulates cell property in nasopharyngeal carcinoma through Wnt/β-catenin signaling pathway. Stem Cell Res.

[168] Tang LQ, Li CF, Li J (2015). Establishment and validation of prognostic nomograms for endemic nasopharyngeal carcinoma. J Natl Cancer Inst.

[169] Tang LQ, Li CF, Chen QY (2015). High-sensitivity C-reactive protein complements plasma Epstein–Barr virus deoxyribonucleic acid prognostication in nasopharyngeal carcinoma: a large-scale retrospective and prospective cohort study. Int J Radiat Oncol Biol Phys.

[170] Chou J, Lin YC, Kim J (2008). Nasopharyngeal carcinoma—review of the molecular mechanisms of tumorigenesis. Head Neck.

[171] Zhang L, MacIsaac KD, Zhou T (2017). Genomic analysis of nasopharyngeal carcinoma reveals TME-based subtypes. Mol Cancer Res.

[172] Liao Q, Guo X, Li X (2012). Analysis of the contribution of nasopharyngeal epithelial cancer cells to the induction of a local inflammatory response. J Cancer Res Clin Oncol.

[173] Pathmanathan R, Prasad U, Sadler R (1995). Clonal proliferations of cells infected with Epstein–Barr virus in preinvasive lesions related to nasopharyngeal carcinoma. N Engl J Med.

[174] Yang L, Liu L, Xu Z (2015). EBV-LMP1 targeted DNAzyme enhances radiosensitivity by inhibiting tumor angiogenesis via the JNKs/HIF-1 pathway in nasopharyngeal carcinoma. Oncotarget.

[175] Yang L, Xu Z, Liu L (2014). Targeting EBV-LMP1 DNAzyme enhances radiosensitivity of nasopharyngeal carcinoma cells by inhibiting telomerase activity. Cancer Biol Ther.

[176] Ma X, Xu Z, Yang L (2013). EBV-LMP1-targeted DNAzyme induces DNA damage and causes cell cycle arrest in LMP1-positive nasopharyngeal carcinoma cells. Int J Oncol.

[177] Yang L, Lu Z, Ma X (2010). A therapeutic approach to nasopharyngeal carcinomas by DNAzymes targeting EBV LMP-1 gene. Molecules.

[178] Cao Y, Yang L, Jiang W (2014). Therapeutic evaluation of Epstein–Barr virus-encoded latent membrane protein-1 targeted DNAzyme for treating of nasopharyngeal carcinomas. Mol Ther.

[179] Liao WH, Yang LF, Liu XY (2014). DCE-MRI assessment of the effect of Epstein–Barr virus-encoded latent membrane protein-1 targeted DNAzyme on tumor vasculature in patients with nasopharyngeal carcinomas. BMC Cancer.

[180] Jiang L, Lan R, Huang T (2017). EBNA1-targeted probe for the imaging and growth inhibition of tumours associated with the Epstein–Barr virus. Nat Biomed Eng.

[181] Shaw P, Senthilnathan R, Krishnan S (2021). A clinical update on the prognostic effect of microRNA biomarkers for survival outcome in nasopharyngeal carcinoma: a systematic review and meta-analysis. Cancers.

[182] Liu F, Tai Y, Ma J (2018). LncRNA NEAT1/let-7a-5p axis regulates the cisplatin resistance in nasopharyngeal carcinoma by targeting Rsf-1 and modulating the Ras-MAPK pathway. Cancer Biol Ther.

[183] Ma X, Zhou J, Liu J (2018). LncRNA ANCR promotes proliferation and radiation resistance of nasopharyngeal carcinoma by inhibiting PTEN expression. Onco Targets Ther.

[184] Jin C, Yan B, Lu Q (2016). The role of MALAT1/miR-1/slug axis on radioresistance in nasopharyngeal carcinoma. Tumour Biol.

[185] Zhao L, Tang M, Hu Z (2015). miR-504 mediated down-regulation of nuclear respiratory factor 1 leads to radio-resistance in nasopharyngeal carcinoma. Oncotarget.

[186] Hong X, Liu N, Liang Y (2020). Circular RNA CRIM1 functions as a ceRNA to promote nasopharyngeal carcinoma metastasis and docetaxel chemoresistance through upregulating FOXQ1. Mol Cancer.

[187] Li Y, Ju K, Wang W (2018). Dinitrosopiperazine-decreased PKP3 through upregulating miR-149 participates in nasopharyngeal carcinoma metastasis. Mol Carcinog.

[188] Wu J, Du M, Zhang Q (2018). Long noncoding RNA UCA1 promotes the proliferation, invasion, and migration of nasopharyngeal carcinoma cells via modulation of miR-145. Onco Targets Ther.

[189] Su X, Li G, Liu W (2017). The long noncoding RNA cancer susceptibility candidate 9 promotes nasophatryngeal carcinogenesis via stabilizing HIF1α. DNA Cell Biol.

[190] Tang XR, Wen X, He QM (2017). MicroRNA-101 inhibits invasion and angiogenesis through targeting ITGA3 and its systemic delivery inhibits lung metastasis in nasopharyngeal carcinoma. Cell Death Dis.

[191] He Y, Jing Y, Wei F (2018). Long non-coding RNA PVT1 predicts poor prognosis and induces radioresistance by regulating DNA repair and cell apoptosis in nasopharyngeal carcinoma. Cell Death Dis.

[192] Chak WP, Lung RWM, Tong JHM (2017). Downregulation of long non-coding RNA MEG3 in nasopharyngeal carcinoma. Mol Carcinog.

[193] Miao WJ, Yuan DJ, Zhang GZ (2019). lncRNA CASC2/miR-18a-5p axis regulates the malignant potential of nasopharyngeal carcinoma by targeting RBBP8. Oncol Rep.

[194] Zheng ZQ, Li ZX, Zhou GQ (2019). Long noncoding RNA FAM225A promotes nasopharyngeal carcinoma tumorigenesis and metastasis by acting as ceRNA to sponge miR-590-3p/miR-1275 and upregulate ITGB3. Cancer Res.

[195] Hu J, Pan J, Luo Z (2021). Long non-coding RNA FOXD3-AS1 silencing exerts tumor suppressive effects in nasopharyngeal carcinoma by downregulating FOXD3 expression via microRNA-185-3p upregulation. Cancer Gene Ther.

[196] Feng S, Wang Y, Zhang R (2017). Curcumin exerts its antitumor activity through regulation of miR-7/Skp2/p21 in nasopharyngeal carcinoma cells. Onco Targets Ther.

[197] Fang LL, Wang XH, Sun BF (2017). Expression, regulation and mechanism of action of the miR-17-92 cluster in tumor cells (Review). Int J Mol Med.

[198] Chen C, Lu Z, Yang J (2016). MiR-17-5p promotes cancer cell proliferation and tumorigenesis in nasopharyngeal carcinoma by targeting p21. Cancer Med.

[199] Zhao F, Pu Y, Qian L (2017). MiR-20a-5p promotes radio-resistance by targeting NPAS2 in nasopharyngeal cancer cells. Oncotarget.

[200] Shi L, Yin W, Zhang Z (2016). Down-regulation of miR-26b induces cisplatin resistance in nasopharyngeal carcinoma by repressing JAG1. FEBS Open Bio.

[201] He S, Yang S, Niu M (2018). HMG-box transcription factor 1: a positive regulator of the G1/S transition through the Cyclin-CDK-CDKI molecular network in nasopharyngeal carcinoma. Cell Death Dis.

[202] Han Q, Li L, Liang H (2017). Downregulation of lncRNA X inactive specific transcript (XIST) suppresses cell proliferation and enhances radiosensitivity by upregulating mir-29c in nasopharyngeal carcinoma cells. Med Sci Monit.

[203] Tan GW, Sivanesan VM, Abdul Rahman FI (2019). A novel and non-invasive approach utilising nasal washings for the detection of nasopharyngeal carcinoma. Int J Cancer.

[204] Sun Q, Liu T, Zhang T (2015). MiR-101 sensitizes human nasopharyngeal carcinoma cells to radiation by targeting stathmin 1. Mol Med Rep.

[205] Wu M, Ye X, Deng X (2016). Upregulation of metastasis-associated gene 2 promotes cell proliferation and invasion in nasopharyngeal carcinoma. Onco Targets Ther.

[206] Li X, Liu F, Lin B (2017). miR-150 inhibits proliferation and tumorigenicity via retarding G1/S phase transition in nasopharyngeal carcinoma. Int J Oncol.

[207] Xu J, Ai Q, Cao H (2015). MiR-185-3p and miR-324-3p predict radiosensitivity of nasopharyngeal carcinoma and modulate cancer cell growth and apoptosis by targeting SMAD7. Med Sci Monit.

[208] Jiang C, Wang H, Zhou L (2017). MicroRNA-212 inhibits the metastasis of nasopharyngeal carcinoma by targeting SOX4. Oncol Rep.

[209] Zhang HQ, Sun Y, Li JQ (2017). The expression of microRNA-324-3p as a tumor suppressor in nasopharyngeal carcinoma and its clinical significance. Onco Targets Ther.

[210] Wang T, Du M, Zhang W (2019). MicroRNA-432 suppresses invasion and migration via E2F3 in nasopharyngeal carcinoma. Onco Targets Ther.

[211] Tang Y, He Y, Shi L (2017). Co-expression of AFAP1-AS1 and PD-1 predicts poor prognosis in nasopharyngeal carcinoma. Oncotarget.

[212] Hu X, Liu W, Jiang X (2019). Long noncoding RNA LINC00460 aggravates invasion and metastasis by targeting miR-30a-3p/Rap1A in nasopharyngeal carcinoma. Hum Cell.

[213] Zhang W, Guo Q, Liu G (2019). NKILA represses nasopharyngeal carcinoma carcinogenesis and metastasis by NF-κB pathway inhibition. PLoS Genet.

[214] Liao B, Wang Z, Zhu Y (2019). Long noncoding RNA DRAIC acts as a microRNA-122 sponge to facilitate nasopharyngeal carcinoma cell proliferation, migration and invasion via regulating SATB1. Artif Cells Nanomed Biotechnol.

[215] Dai BQ, Jiang X, Feng LC (2021). LncRNA TP73-AS1 regulates miR-495 expression to promote migration and invasion of nasopharyngeal carcinoma cells through junctional adhesion molecule A. Kaohsiung J Med Sci.

[216] Bruce JP, Yip K, Bratman SV (2015). Nasopharyngeal cancer: molecular landscape. J Clin Oncol.

[217] Li YY, Chung GTY, Lui VWY (2017). Exome and genome sequencing of nasopharynx cancer identifies NF-κB pathway activating mutations. Nat Commun.

[218] Wong CH, Ma BBY, Cheong HT (2015). Preclinical evaluation of PI3K inhibitor BYL719 as a single agent and its synergism in combination with cisplatin or MEK inhibitor in nasopharyngeal carcinoma (NPC). Am J Cancer Res.

[219] Zhang Z, Yu X, Zhou Z (2019). LMP1-positive extracellular vesicles promote radioresistance in nasopharyngeal carcinoma cells through P38 MAPK signaling. Cancer Med.

[220] Li SS, Yang S, Yin DH (2016). Inhibition of PI3K-Akt pathway reverses LMP1 induced TRAIL resistance in nasopharyngeal carcinoma cell. Lin Chung Er Bi Yan Hou Tou Jing Wai Ke Za Zhi.

[221] Da YG, Huang TJ, Peng LX (2013). Epstein–Barr virus_encoded LMP1 upregulates microRNA-21 to promote the resistance of nasopharyngeal carcinoma cells to cisplatin-induced apoptosis by suppressing PDCD4 and Fas-L. PLoS ONE.

[222] Chen Y-R, Liu M-T, Chang Y-T (2008). Epstein–Barr virus latent membrane protein 1 represses DNA repair through the PI3K/Akt/FOXO3a pathway in human epithelial cells. J Virol.

[223] Xuechi L, Sufang L, Xiangjian L (2009). EBV-encoded LMP1 regulates Op18/stathmin signaling pathway by cdc2 mediation in nasopharyngeal carcinoma cells. Int J Cancer.

[224] Kordbacheh F, Farah CS (2021). Current and emerging molecular therapies for head and neck squamous cell carcinoma. Cancers.

[225] Yom SS, Wee J (2014). Nasopharyngeal carcinoma: management strategies.

[226] Ma CX, Luo J, Naughton M (2016). A phase I trial of BKM120 (buparlisib) in combination with fulvestrant in postmenopausal women with estrogen receptor-positive metastatic breast cancer. Clin Cancer Res.

[227] Rodon J, Curigliano G, Delord JP (2018). A phase Ib, open-label, dose-finding study of alpelisib in combination with paclitaxel in patients with advanced solid tumors. Oncotarget.

[228] Mishra R, Patel H, Alanazi S (2021). PI3K inhibitors in cancer: clinical implications and adverse effects. Int J Mol Sci.

[229] Wong KK, Engelman JA, Cantley LC (2010). Targeting the PI3K signaling pathway in cancer. Curr Opin Genet Dev.

[230] Chen Q, Zheng W, Zhu L (2020). ANXA6 contributes to radioresistance by promoting autophagy via inhibiting the PI3K/AKT/mTOR signaling pathway in nasopharyngeal carcinoma. Front cell Dev Biol.

[231] Li WY, Li Q, Jing L (2019). P57-mediated autophagy promotes the efficacy of EGFR inhibitors in hepatocellular carcinoma. Liver Int.

[232] Deng L, Yang J, Zhao XR (2003). Cells in G2/M phase increased in human nasopharyngeal carcinoma cell line by EBV-LMP1 through activation of NF-κB and AP-1. Cell Res.

[233] Zhou G, Liu Z, Myers JN (2016). TP53 mutations in head and neck squamous cell carcinoma and their impact on disease progression and treatment response. J Cell Biochem.

[234] Zheng X, Wang J, Wei L (2018). Epstein–Barr virus microRNA miR-BART5-3p inhibits p53 expression. J Virol.

[235] Li Z, Chen X, Li L (2012). EBV encoded miR-BHRF1-1 potentiates viral lytic replication by downregulating host p53 in nasopharyngeal carcinoma. Int J Biochem Cell Biol.

[236] Hao W, Zhu Y, Wang H (2021). miR-4270 modulates the irradiation-sensitivity of nasopharyngeal carcinoma cells through modulation of p53 in vivo. Tohoku J Exp Med.

[237] Mendez E, Rodriguez CP, Kao MC (2018). A phase I clinical trial of AZD1775 in combination with neoadjuvant weekly docetaxel and cisplatin before definitive therapy in head and neck squamous cell carcinoma. Clin Cancer Res.

[238] Voon YL, Ahmad M, Wong PF (2015). Nutlin-3 sensitizes nasopharyngeal carcinoma cells to cisplatin-induced cytotoxicity. Oncol Rep.

[239] Khurana A, Shafer DA (2019). MDM2 antagonists as a novel treatment option for acute myeloid leukemia: perspectives on the therapeutic potential of idasanutlin (RG7388). Onco Targets Ther.

[240] Ding Q, Zhang Z, Liu JJ (2013). Discovery of RG7388, a potent and selective p53-MDM2 inhibitor in clinical development. J Med Chem.

[241] Salim KY, MalekiVareki S, Danter WR (2016). COTI-2, a novel small molecule that is active against multiple human cancer cell lines in vitro and in vivo. Oncotarget.

[242] Roszkowska KA, Piecuch A, Sady M (2022). Gain of function (GOF) mutant p53 in cancer—current therapeutic approaches. Int J Mol Sci.

[243] Chung GTY, Lou WPK, Chow C (2013). Constitutive activation of distinct NF-κB signals in EBV-associated nasopharyngeal carcinoma. J Pathol.

[244] Wang FH, Wei XL, Feng J (2021). Efficacy, safety, and correlative biomarkers of toripalimab in previously treated recurrent or metastatic nasopharyngeal carcinoma: a phase II clinical trial (POLARIS-02). J Clin Oncol.

[245] You R, Liu YP, Lin DC (2019). Clonal mutations activate the NF-κB pathway to promote recurrence of nasopharyngeal carcinoma. Cancer Res.

[246] Yi M, Cai J, Li J (2018). Rediscovery of NF-κB signaling in nasopharyngeal carcinoma: how genetic defects of NF-κB pathway interplay with EBV in driving oncogenesis?. J Cell Physiol.

[247] Liu Y, Gong P, Zhou N (2019). Insufficient PINX1 expression stimulates telomerase activation by direct inhibition of EBV LMP1-NF-κB axis during nasopharyngeal carcinoma development. Biochem Biophys Res Commun.

[248] Leão R, Apolónio JD, Lee D (2018). Mechanisms of human telomerase reverse transcriptase (hTERT) regulation: clinical impacts in cancer. J Biomed Sci.

[249] Ding L, Li LL, Yang J (2005). Epstein–Barr virus encoded latent membrane protein 1 modulates nuclear translocation of telomerase reverse transcriptase protein by activating nuclear factor-kappaB p65 in human nasopharyngeal carcinoma cells. Int J Biochem Cell Biol.

[250] Stewart S, Dawson CW, Takada K (2004). Epstein–Barr virus-encoded LMP2A regulates viral and cellular gene expression by modulation of the NF-kappaB transcription factor pathway. Proc Natl Acad Sci USA.

[251] Chow SE, Chien HT, Chu WK (2021). Human caspase 12 enhances NF-κB activity through activation of IKK in nasopharyngeal carcinoma cells. Int J Mol Sci.

[252] Zhu DD, Zhang J, Deng W (2016). Significance of NF-κB activation in immortalization of nasopharyngeal epithelial cells. Int J Cancer.

[253] Zhou M, Zhao G, Zhou X (2022). Clinicopathological analysis and prognostic significance of NF-κB p65 and IKKβ protein and mRNA expression in nasopharyngeal carcinoma. J Int Med Res.

[254] Taylor MH, Betts CB, Maloney L (2022). Safety and efficacy of pembrolizumab in combination with acalabrutinib in advanced head and neck squamous cell carcinoma: phase 2 proof-of-concept study. Clin Cancer Res.

[255] Gomez-Roca C, Even C, Le Tourneau C (2022). Exploratory window-of-opportunity trial to investigate the tumor pharmacokinetics/pharmacodynamics of the IAP antagonist Debio 1143 in patients with head and neck cancer. Clin Transl Sci.

[256] Matzinger O, Viertl D, Tsoutsou P (2015). The radiosensitizing activity of the SMAC-mimetic, Debio 1143, is TNFα-mediated in head and neck squamous cell carcinoma. Radiother Oncol.

[257] Tucker N. FDA grants breakthrough therapy designation to Debio 1143 in front-line head and neck cancer. 2020. https://www.targetedonc.com/view/fda-grants-breakthrough-therapy-designation-to-debio-1143-in-frontline-head-and-neck-cancer. Accessed 11 Sept 2023.

[258] Attinger A, Gavillet B, Chessex AV (2018). Abstract 4703: The inhibitor of apoptosis protein (IAP) antagonist Debio 1143 enhances the immune response to anti-PD1/L1 inhibitors in vitro and in vivo. Cancer Res.

[259] Zhang Z, Ma Z, Kang J (2023). Period2-mediated downregulation of ERK/MAPK phosphorylation in nasopharyngeal carcinoma. medRxiv.

[260] Zhang Y-J, Zhang M-F, Zhou H-F (2018). Activation of c-Jun/JNK signaling predicts poor prognosis in nasopharyngeal carcinoma. Int J Clin Exp Pathol.

[261] Li Z, Li N, Shen L (2018). MAP2K6 is associated with radiation resistance and adverse prognosis for locally advanced nasopharyngeal carcinoma patients. Cancer Manag Res.

[262] Lo AKF, Liu Y, Wang XH (2003). Alterations of biologic properties and gene expression in nasopharyngeal epithelial cells by the Epstein–Barr virus-encoded latent membrane protein 1. Lab Invest.

[263] Li L, Guo L, Tao Y (2007). Latent membrane protein 1 of Epstein–Barr virus regulates p53 phosphorylation through MAP kinases. Cancer Lett.

[264] Tsai CL, Li HP, Lu YJ (2006). Activation of DNA methyltransferase 1 by EBV LMP1 Involves c-Jun NH(2)-terminal kinase signaling. Cancer Res.

[265] Rosen LS, LoRusso P, Ma WW (2016). A first-in-human phase I study to evaluate the MEK1/2 inhibitor, cobimetinib, administered daily in patients with advanced solid tumors. Invest New Drugs.

[266] Adjei AA, LoRusso PM, Ribas A (2012). Multicenter, dose-escalation study of the investigational drug tak-733, an oral mek inhibitor, in patients (PTS) with advanced solid tumors: preliminary phase 1 results. Ann Oncol.

[267] Uppaluri R, Winkler AE, Lin T (2017). Biomarker and tumor responses of oral cavity squamous cell carcinoma to trametinib: a phase II neoadjuvant window-of-opportunity clinical trial. Clin Cancer Res.

[268] Robert C, Karaszewska B, Schachter J (2015). Improved overall survival in melanoma with combined dabrafenib and trametinib. N Engl J Med.

[269] Long GV, Hauschild A, Santinami M (2017). Adjuvant dabrafenib plus trametinib in stage III BRAF-mutated melanoma. N Engl J Med.

[270] Zhao Y, Adjei AA (2014). The clinical development of MEK inhibitors. Nat Rev Clin Oncol.

[271] Si Y, Xu J, Meng L (2022). Role of STAT3 in the pathogenesis of nasopharyngeal carcinoma and its significance in anticancer therapy. Front Oncol.

[272] Wei N, Li J, Fang C (2019). Targeting colon cancer with the novel STAT3 inhibitor bruceantinol. Oncogene.

[273] Hsiao JR, Jin YT, Tsai ST (2003). Constitutive activation of STAT3 and STAT5 is present in the majority of nasopharyngeal carcinoma and correlates with better prognosis. Br J Cancer.

[274] Cheng JZ, Chen JJ, Xue K (2018). Clinicopathologic and prognostic significance of VEGF, JAK2 and STAT3 in patients with nasopharyngeal carcinoma. Cancer Cell Int.

[275] Zhuang M, Ding X, Song W (2021). Correlation of IL-6 and JAK2/STAT3 signaling pathway with prognosis of nasopharyngeal carcinoma patients. Aging.

[276] Togi S, Hatano Y, Muromoto R (2016). Caspase-dependent cleavage regulates protein levels of Epstein–Barr virus-derived latent membrane protein 1. FEBS Lett.

[277] Liu YP, Tan YN, Wang ZL (2008). Phosphorylation and nuclear translocation of STAT3 regulated by the Epstein–Barr virus latent membrane protein 1 in nasopharyngeal carcinoma. Int J Mol Med.

[278] Chen H, Hutt-Fletcher L, Cao L (2003). A positive autoregulatory loop of LMP1 expression and STAT activation in epithelial cells latently infected with Epstein–Barr virus. J Virol.

[279] Wang Z, Luo F, Li L (2010). STAT3 activation induced by Epstein–Barr virus latent membrane protein1 causes vascular endothelial growth factor expression and cellular invasiveness via JAK3 And ERK signaling. Eur J Cancer.

[280] Bousoik E, Montazeri AH (2018). ‘Do we know Jack’ about JAK? A closer look at JAK/STAT signaling pathway. Front Oncol.

[281] Chen H, Lee JM, Zong Y (2001). Linkage between STAT regulation and Epstein–Barr virus gene expression in tumors. J Virol.

[282] Simon SE, Ahmed U, Saad SM (2022). New synthetic phenylquinazoline derivatives induce apoptosis by targeting the pro-survival members of the BCL-2 family. Bioorg Med Chem Lett.

[283] Qing X, Tan GL, Liu HW (2020). LINC00669 insulates the JAK/STAT suppressor SOCS1 to promote nasopharyngeal cancer cell proliferation and invasion. J Exp Clin Cancer Res.

[284] Johnson DE, O’Keefe RA, Grandis JR (2018). Targeting the IL-6/JAK/STAT3 signalling axis in cancer. Nat Rev Clin Oncol.

[285] Sen M, Thomas SM, Kim S (2012). First-in-human trial of a STAT3 decoy oligonucleotide in head and neck tumors: implications for cancer therapy. Cancer Discov.

[286] Xu G, Zhu L, Wang Y (2017). Stattic enhances radiosensitivity and reduces radio-induced migration and invasion in HCC cell lines through an apoptosis pathway. Biomed Res Int.

[287] Zhang Q, Zhang C, He J (2015). STAT3 inhibitor stattic enhances radiosensitivity in esophageal squamous cell carcinoma. Tumour Biol.

[288] Pan Y, Zhou F, Zhang R (2013). Stat3 inhibitor stattic exhibits potent antitumor activity and induces chemo- and radio-sensitivity in nasopharyngeal carcinoma. PLoS ONE.

[289] Qureshy Z, Li H, Zeng Y (2022). STAT3 activation as a predictive biomarker for ruxolitinib response in head and neck cancer. Clin Cancer Res.

[290] Bose P, Verstovsek S (2020). JAK inhibition for the treatment of myelofibrosis: limitations and future perspectives. HemaSphere.

[291] Cohen EE, Hong DS, Wise Draper T (2017). Phase 1b/2 study (SCORES) assessing safety, tolerability, and preliminary anti-tumor activity of durvalumab plus AZD9150 or AZD5069 in patients with advanced solid malignancies and squamous cell carcinoma of the head and neck (SCCHN). Ann Oncol.

[292] Li G, Su Q, Liu H (2018). Frizzled7 promotes epithelial-to-mesenchymal transition and stemness via activating canonical Wnt/β-catenin pathway in gastric cancer. Int J Biol Sci.

[293] Ngernsombat C, Prattapong P, Larbcharoensub N (2021). WNT8B as an independent prognostic marker for nasopharyngeal carcinoma. Curr Oncol.

[294] Jin PY, Zheng ZH, Lu HJ (2019). Roles of β-catenin, TCF-4, and survivin in nasopharyngeal carcinoma: correlation with clinicopathological features and prognostic significance. Cancer Cell Int.

[295] Zhou LQ, Shen JX, Zhou T (2022). The prognostic significance of β-catenin expression in patients with nasopharyngeal carcinoma: a systematic review and meta-analysis. Front Genet.

[296] Lin Z, Wan X, Jiang R (2014). Epstein–Barr virus-encoded latent membrane protein 2A promotes the epithelial–mesenchymal transition in nasopharyngeal carcinoma via metastatic tumor antigen 1 and mechanistic target of rapamycin signaling induction. J Virol.

[297] Huang Y, Tan D, Xiao J (2018). miR-150 contributes to the radioresistance in nasopharyngeal carcinoma cells by targeting glycogen synthase kinase-3β. J Cancer Res Ther.

[298] Arend RC, Londoño-Joshi AI, Samant RS (2014). Inhibition of Wnt/β-catenin pathway by niclosamide: a therapeutic target for ovarian cancer. Gynecol Oncol.

[299] Li B, Flaveny CA, Giambelli C (2014). Repurposing the FDA-approved pinworm drug pyrvinium as a novel chemotherapeutic agent for intestinal polyposis. PLoS ONE.

[300] Tai WP, Hu PJ, Wu J (2014). The inhibition of Wnt/β-catenin signaling pathway in human colon cancer cells by sulindac. Tumori.

[301] Bordonaro M, Lazarova DL (2015). CREB-binding protein, p300, butyrate, and Wnt signaling in colorectal cancer. World J Gastroenterol.

[302] Emami KH, Nguyen C, Ma H (2004). A small molecule inhibitor of beta-catenin/CREB-binding protein transcription [corrected]. Proc Natl Acad Sci USA.

[303] Lenz HJ, Kahn M (2014). Safely targeting cancer stem cells via selective catenin coactivator antagonism. Cancer Sci.

[304] Flanagan DJ, Barker N, Di Costanzo NS (2019). Frizzled-7 is required for Wnt signaling in gastric tumors with and without Apc mutations. Cancer Res.

[305] Krishnamurthy N, Kurzrock R (2018). Targeting the Wnt/beta-catenin pathway in cancer: update on effectors and inhibitors. Cancer Treat Rev.

[306] Kahn M (2014). Can we safely target the WNT pathway?. Nat Rev Drug Discov.

[307] Waaler J, Machon O, Tumova L (2012). A novel tankyrase inhibitor decreases canonical Wnt signaling in colon carcinoma cells and reduces tumor growth in conditional APC mutant mice. Cancer Res.

[308] Gonsalves FC, Klein K, Carson BB (2011). An RNAi-based chemical genetic screen identifies three small-molecule inhibitors of the Wnt/wingless signaling pathway. Proc Natl Acad Sci USA.

[309] Huang SMA, Mishina YM, Liu S (2009). Tankyrase inhibition stabilizes axin and antagonizes Wnt signalling. Nature.

[310] Eguchi M, Nguyen C, Lee S (2005). ICG-001, a novel small molecule regulator of TCF/beta-catenin transcription. Med Chem.

[311] Qiu GH, Tan LKS, Loh KS (2004). The candidate tumor suppressor gene BLU, located at the commonly deleted region 3p21.3, is an E2F-regulated, stress-responsive gene and inactivated by both epigenetic and genetic mechanisms in nasopharyngeal carcinoma. Oncogene.

[312] Kwong J, Lo KW, To KF, Teo PM, Johnson PJ, Huang DP (2002). Promoter hypermethylation of multiple genes in nasopharyngeal carcinoma. Clin Cancer Res.

[313] Chan KC, Ko JMY, Lung HL (2011). Catalytic activity of matrix metalloproteinase-19 is essential for tumor suppressor and anti-angiogenic activities in nasopharyngeal carcinoma. Int J Cancer.

[314] Cheung HW, Ching YP, Nicholls JM (2005). Epigenetic inactivation of CHFR in nasopharyngeal carcinoma through promoter methylation. Mol Carcinog.

[315] Fan J, Zhang Y, Mu J (2018). TET1 exerts its anti-tumor functions via demethylating DACT2 and SFRP2 to antagonize Wnt/β-catenin signaling pathway in nasopharyngeal carcinoma cells. Clin Epigenet.

[316] Dai W, Cheung AKL, Ko JMY (2015). Comparative methylome analysis in solid tumors reveals aberrant methylation at chromosome 6p in nasopharyngeal carcinoma. Cancer Med.

[317] Li L, Zhang Y, Fan Y (2015). Characterization of the nasopharyngeal carcinoma methylome identifies aberrant disruption of key signaling pathways and methylated tumor suppressor genes. Epigenomics.

[318] Peng H, Chen Y, Gong P (2016). Higher methylation intensity induced by EBV LMP1 via NF-κB/DNMT3b signaling contributes to silencing of PTEN gene. Oncotarget.

[319] Luo X, Hong L, Cheng C (2018). DNMT1 mediates metabolic reprogramming induced by Epstein–Barr virus latent membrane protein 1 and reversed by grifolin in nasopharyngeal carcinoma. Cell Death Dis.

[320] Krishna SM, Kattoor J, Balaram P (2005). Down regulation of adhesion protein E-cadherin in Epstein–Barr virus infected nasopharyngeal carcinomas. Cancer Biomark.

[321] Tulalamba W, Janvilisri T (2012). Nasopharyngeal carcinoma signaling pathway: an update on molecular biomarkers. Int J Cell Biol.

[322] Shao Y, Jiang H, Wu X (2014). p16 promoter hypermethylation is associated with increased risk of nasopharyngeal carcinoma. Mol Clin Oncol.

[323] Hsu CL, Lui KW, Chi LM (2018). Integrated genomic analyses in PDX model reveal a cyclin-dependent kinase inhibitor palbociclib as a novel candidate drug for nasopharyngeal carcinoma. J Exp Clin Cancer Res.

[324] Li L, Xu J, Qiu G (2018). Epigenomic characterization of a p53-regulated 3p22.2 tumor suppressor that inhibits STAT3 phosphorylation via protein docking and is frequently methylated in esophageal and other carcinomas. Theranostics.

[325] Cheung AKL, Lung HL, Hung SC (2008). Functional analysis of a cell cycle-associated, tumor-suppressive gene, protein tyrosine phosphatase receptor type G, in nasopharyngeal carcinoma. Cancer Res.

[326] Kwong J, Chow LSN, Wong AYH (2007). Epigenetic inactivation of the deleted in lung and esophageal cancer 1 gene in nasopharyngeal carcinoma. Genes Chromosomes Cancer.

[327] Peserico A, Simone C (2011). Physical and functional HAT/HDAC interplay regulates protein acetylation balance. J Biomed Biotechnol.

[328] Murata T, Kondo Y, Sugimoto A (2012). Epigenetic histone modification of Epstein–Barr virus BZLF1 promoter during latency and reactivation in Raji cells. J Virol.

[329] Cai MY, Tong ZT, Zhu W (2011). H3K27me3 protein is a promising predictive biomarker of patients’ survival and chemoradioresistance in human nasopharyngeal carcinoma. Mol Med.

[330] Ke L, Zhou H, Wang C (2017). Nasopharyngeal carcinoma super-enhancer-driven ETV6 correlates with prognosis. Proc Natl Acad Sci USA.

[331] Leong MML, Lung ML (2021). The impact of Epstein–Barr virus infection on epigenetic regulation of host cell gene expression in epithelial and lymphocytic malignancies. Front Oncol.

[332] Leong MML, Cheung AKL, Dai W (2019). EBV infection is associated with histone bivalent switch modifications in squamous epithelial cells. Proc Natl Acad Sci USA.

[333] Wu C, Guo E, Ming J (2020). Radiation-induced DNMT3B promotes radioresistance in nasopharyngeal carcinoma through methylation of p53 and p21. Mol Ther Oncolytics.

[334] He Y, Wang Z, Liu C (2019). Protocadherin 17 is a tumor suppressor and is frequently methylated in nasopharyngeal carcinoma. Cancer Manag Res.

[335] Xie J, Wang Z, Fan W (2021). Targeting cancer cell plasticity by HDAC inhibition to reverse EBV-induced dedifferentiation in nasopharyngeal carcinoma. Signal Transduct Target Ther.

[336] Place RF, Noonan EJ, Giardina C (2005). HDAC inhibition prevents NF-kappa B activation by suppressing proteasome activity: down-regulation of proteasome subunit expression stabilizes I kappa B alpha. Biochem Pharmacol.

[337] Suresh PS, Devaraj VC, Srinivas NR (2017). Review of bioanalytical assays for the quantitation of various HDAC inhibitors such as vorinostat, belinostat, panobinostat, romidepsin and chidamine. Biomed Chromatogr.

[338] Lee J, Huang S (2013). Cancer epigenetics: mechanisms and crosstalk of a HDAC inhibitor, vorinostat. Chemotherapy.

[339] Grant C, Rahman F, Piekarz R (2010). Romidepsin: a new therapy for cutaneous T-cell lymphoma and a potential therapy for solid tumors. Expert Rev Anticancer Ther.

[340] Bolden JE, Peart MJ, Johnstone RW (2006). Anticancer activities of histone deacetylase inhibitors. Nat Rev Drug Discov.

[341] Jiang W, Li YQ, Liu N (2014). 5-Azacytidine enhances the radiosensitivity of CNE2 and SUNE1 cells in vitro and in vivo possibly by altering DNA methylation. PLoS ONE.

[342] Von Hoff DD, Rasco DW, Heath EI (2018). Phase I study of CC-486 alone and in combination with carboplatin or nab-paclitaxel in patients with relapsed or refractory solid tumors. Clin Cancer Res.

[343] Mesia R, Bossi P, Hansen AR (2019). Phase II study of CC-486 (oral azacitidine) in previously treated patients with locally advanced or metastatic nasopharyngeal carcinoma. Eur J Cancer.

[344] Hui KF, Cheung AKL, Choi CK (2016). Inhibition of class I histone deacetylases by romidepsin potently induces Epstein–Barr virus lytic cycle and mediates enhanced cell death with ganciclovir. Int J Cancer.

[345] Hui KF, Ho DN, Tsang CM (2012). Activation of lytic cycle of Epstein–Barr virus by suberoylanilide hydroxamic acid leads to apoptosis and tumor growth suppression of nasopharyngeal carcinoma. Int J Cancer.

[346] Gressette M, Vérillaud B, Jimenez-Pailhès AS (2014). Treatment of nasopharyngeal carcinoma cells with the histone-deacetylase inhibitor abexinostat: cooperative effects with cis-platin and radiotherapy on patient-derived xenografts. PLoS ONE.

[347] Ding S, Gao Y, Lv D (2022). DNTTIP1 promotes nasopharyngeal carcinoma metastasis via recruiting HDAC1 to DUSP2 promoter and activating ERK signaling pathway. EBioMedicine.

[348] Chen DS, Mellman I (2013). Oncology meets immunology: the cancer-immunity cycle. Immunity.

[349] Chan ATC, Tao Q, Robertson KD (2004). Azacitidine induces demethylation of the Epstein–Barr virus genome in tumors. J Clin Oncol.

[350] Gourzones C, Barjon C, Busson P (2012). Host–tumor interactions in nasopharyngeal carcinomas. Semin Cancer Biol.

[351] Lai FMM, Cheng PNM, Tsao SY (1990). Immunohistological characteristics of the infiltrating lymphoid cells and expression of HLA class I and II antigens in nasopharyngeal carcinoma. Virchows Arch A Pathol Anat Histopathol.

[352] Chong P, Chew C, Chan K (1988). Tumour infiltrating lymphocytes in nasopharyngeal carcinoma. Ann Acad Med Singap.

[353] Su ZY, Siak PY, Leong CO (2022). Nasopharyngeal carcinoma and its microenvironment: past, current, and future perspectives. Front Oncol.

[354] Jin S, Li R, Chen MY (2020). Single-cell transcriptomic analysis defines the interplay between tumor cells, viral infection, and the microenvironment in nasopharyngeal carcinoma. Cell Res.

[355] Lo AKF, Dawson CW, Lung HL (2021). The role of EBV-encoded LMP1 in the NPC tumor microenvironment: from function to therapy. Front Oncol.

[356] Hurwitz SN, Nkosi D, Conlon MM (2017). CD63 regulates Epstein–Barr virus LMP1 exosomal packaging, enhancement of vesicle production, and noncanonical NF-κB signaling. J Virol.

[357] Aga M, Bentz GL, Raffa S (2014). Exosomal HIF1α supports invasive potential of nasopharyngeal carcinoma-associated LMP1-positive exosomes. Oncogene.

[358] Keryer-Bibens C, Pioche-Durieu C, Villemant C (2006). Exosomes released by EBV-infected nasopharyngeal carcinoma cells convey the viral latent membrane protein 1 and the immunomodulatory protein galectin 9. BMC Cancer.

[359] Middeldorp JM, Pegtel DM (2008). Multiple roles of LMP1 in Epstein–Barr virus induced immune escape. Semin Cancer Biol.

[360] Fang W, Zhang J, Hong S (2014). EBV-driven LMP1 and IFN-γ up-regulate PD-L1 in nasopharyngeal carcinoma: implications for oncotargeted therapy. Oncotarget.

[361] Cai TT, Ye SB, Liu YN (2017). LMP1-mediated glycolysis induces myeloid-derived suppressor cell expansion in nasopharyngeal carcinoma. PLoS Pathog.

[362] Bentz GL, Shackelford J, Pagano JS (2012). Epstein–Barr virus latent membrane protein 1 regulates the function of interferon regulatory factor 7 by inducing its sumoylation. J Virol.

[363] Dawson CW, Port RJ, Young LS (2012). The role of the EBV-encoded latent membrane proteins LMP1 and LMP2 in the pathogenesis of nasopharyngeal carcinoma (NPC). Semin Cancer Biol.

[364] Zhang CX, Huang DJ, Baloche V (2020). Galectin-9 promotes a suppressive microenvironment in human cancer by enhancing STING degradation. Oncogenesis.

[365] Shah KM, Stewart SE, Wei W (2009). The EBV-encoded latent membrane proteins, LMP2A and LMP2B, limit the actions of interferon by targeting interferon receptors for degradation. Oncogene.

[366] Mrizak D, Martin N, Barjon C (2014). Effect of nasopharyngeal carcinoma-derived exosomes on human regulatory T cells. J Natl Cancer Inst.

[367] Hooykaas MJG, van Gent M, Soppe JA (2017). EBV microRNA BART16 suppresses type I IFN signaling. J Immunol.

[368] Lu Y, Qin Z, Wang J (2017). Epstein–Barr virus miR-BART6-3p inhibits the RIG-I pathway. J Innate Immun.

[369] Tagawa T, Albanese M, Bouvet M (2016). Epstein–Barr viral miRNAs inhibit antiviral CD4+ T cell responses targeting IL-12 and peptide processing. J Exp Med.

[370] Nachmani D, Stern-Ginossar N, Sarid R (2009). Diverse herpesvirus microRNAs target the stress-induced immune ligand MICB to escape recognition by natural killer cells. Cell Host Microbe.

[371] Albanese M, Tagawa T, Bouvet M (2016). Epstein–Barr virus microRNAs reduce immune surveillance by virus-specific CD8+ T cells. Proc Natl Acad Sci USA.

[372] Samanta M, Takada K (2010). Modulation of innate immunity system by Epstein–Barr virus-encoded non-coding RNA and oncogenesis. Cancer Sci.

[373] Gao L, Han H, Wang H (2019). IL-10 knockdown with siRNA enhances the efficacy of doxorubicin chemotherapy in EBV-positive tumors by inducing lytic cycle via PI3K/p38 MAPK/NF-kB pathway. Cancer Lett.

[374] Samanta M, Iwakiri D, Takada K (2008). Epstein–Barr virus-encoded small RNA induces IL-10 through RIG-I-mediated IRF-3 signaling. Oncogene.

[375] Chan OSH, Kowanetz M, Ng WT (2017). Characterization of PD-L1 expression and immune cell infiltration in nasopharyngeal cancer. Oral Oncol.

[376] Li Y-F, Ding J-W, Liao L-M (2017). Expression of programmed death ligand-1 predicts poor outcome in nasopharyngeal carcinoma. Mol Clin Oncol.

[377] Zhu Q, Cai MY, Chen CL (2017). Tumor cells PD-L1 expression as a favorable prognosis factor in nasopharyngeal carcinoma patients with pre-existing intratumor-infiltrating lymphocytes. Oncoimmunology.

[378] Lee VHF, Lo AWI, Leung CY (2016). Correlation of PD-L1 expression of tumor cells with survival outcomes after radical intensity-modulated radiation therapy for non-metastatic nasopharyngeal carcinoma. PLoS ONE.

[379] Ooft ML, van Ipenburg JA, Braunius WW (2017). Prognostic role of tumor infiltrating lymphocytes in EBV positive and EBV negative nasopharyngeal carcinoma. Oral Oncol.

[380] Lu J, Chen XM, Huang HR (2018). Detailed analysis of inflammatory cell infiltration and the prognostic impact on nasopharyngeal carcinoma. Head Neck.

[381] Ooft ML, van Ipenburg JA, Sanders ME (2018). Prognostic role of tumour-associated macrophages and regulatory T cells in EBV-positive and EBV-negative nasopharyngeal carcinoma. J Clin Pathol.

[382] Makowska A, Braunschweig T, Denecke B (2019). Interferon β and Anti-PD-1/PD-L1 checkpoint blockade cooperate in NK cell-mediated killing of nasopharyngeal carcinoma cells. Transl Oncol.

[383] Jiang Y, Chen M, Nie H (2019). PD-1 and PD-L1 in cancer immunotherapy: clinical implications and future considerations. Hum Vaccin Immunother.

[384] Hamanishi J, Mandai M, Matsumura N (2016). PD-1/PD-L1 blockade in cancer treatment: perspectives and issues. Int J Clin Oncol.

[385] Ma BBY, Lim WT, Goh BC (2018). Antitumor activity of nivolumab in recurrent and metastatic nasopharyngeal carcinoma: an international, multicenter study of the mayo clinic phase 2 consortium (NCI-9742). J Clin Oncol.

[386] Hsu C, Lee SH, Ejadi S (2017). Safety and antitumor activity of pembrolizumab in patients with programmed death-ligand 1-positive nasopharyngeal carcinoma: results of the KEYNOTE-028 study. J Clin Oncol.

[387] Hamid O, Robert C, Daud A (2013). Safety and tumor responses with lambrolizumab (anti-PD-1) in melanoma. N Engl J Med.

[388] Chan ATC, Lee VHF, Hong RL (2023). Pembrolizumab monotherapy versus chemotherapy in platinum-pretreated, recurrent or metastatic nasopharyngeal cancer (KEYNOTE-122): an open-label, randomized, phase III trial. Ann Oncol Off J Eur Soc Med Oncol.

[389] Even C, Wang HM, Li SH (2021). Phase II, randomized study of spartalizumab (PDR001), an anti-PD-1 antibody, versus chemotherapy in patients with recurrent/metastatic nasopharyngeal cancer. Clin Cancer Res.

[390] Huang YF, Xie WJ, Fan HY (2019). Comparative safety of PD-1/PD-L1 inhibitors for cancer patients: systematic review and network meta-analysis. Front Oncol.

[391] Lv JW, Li JY, Luo LN (2019). Comparative safety and efficacy of anti-PD-1 monotherapy, chemotherapy alone, and their combination therapy in advanced nasopharyngeal carcinoma: findings from recent advances in landmark trials. J Immunother Cancer.

[392] Schachter J, Ribas A, Long GV (2017). Pembrolizumab versus ipilimumab for advanced melanoma: final overall survival results of a multicentre, randomised, open-label phase 3 study (KEYNOTE-006). Lancet.

[393] Zaretsky JM, Garcia-Diaz A, Shin DS (2016). Mutations associated with acquired resistance to PD-1 blockade in melanoma. N Engl J Med.

[394] Zhao J, Guo C, Xiong F (2020). Single cell RNA-seq reveals the landscape of tumor and infiltrating immune cells in nasopharyngeal carcinoma. Cancer Lett.

[395] Chen TC, Chen CH, Wang CP (2017). The immunologic advantage of recurrent nasopharyngeal carcinoma from the viewpoint of Galectin-9/Tim-3-related changes in the tumour microenvironment. Sci Rep.

[396] Ke X, Yang YC, Hong SL (2011). EBV-LMP1-targeted DNAzyme restrains nasopharyngeal carcinoma growth in a mouse C666-1 xenograft model. Med Oncol.

[397] Chen R, Zhang D, Mao Y (2012). A human Fab-based immunoconjugate specific for the LMP1 extracellular domain inhibits nasopharyngeal carcinoma growth in vitro and in vivo. Mol Cancer Ther.

[398] Smith C, Tsang J, Beagley L (2012). Effective treatment of metastatic forms of Epstein–Barr virus-associated nasopharyngeal carcinoma with a novel adenovirus-based adoptive immunotherapy. Cancer Res.

[399] Chia WK, Wang WW, Teo M (2012). A phase II study evaluating the safety and efficacy of an adenovirus-ΔLMP1-LMP2 transduced dendritic cell vaccine in patients with advanced metastatic nasopharyngeal carcinoma. Ann Oncol Off J Eur Soc Med Oncol.

[400] Louis CU, Straathof K, Bollard CM (2010). Adoptive transfer of EBV-specific T cells results in sustained clinical responses in patients with locoregional nasopharyngeal carcinoma. J Immunother.

[401] Zeng Y, Si YF, Lan GP (2020). LMP2-DC vaccine elicits specific EBV-LMP2 response to effectively improve immunotherapy in patients with nasopharyngeal cancer. Biomed Environ Sci.

[402] Taylor GS, Jia H, Harrington K (2014). A recombinant modified vaccinia ankara vaccine encoding Epstein–Barr virus (EBV) target antigens: a phase I trial in UK patients with EBV-positive cancer. Clin Cancer Res.

[403] Hui EP, Taylor GS, Jia H (2013). Phase I trial of recombinant modified vaccinia ankara encoding Epstein–Barr viral tumor antigens in nasopharyngeal carcinoma patients. Cancer Res.

[404] Lim DW-T, Ng QS, Hong R-L (2020). Abstract CT203: a phase II trial of ipilimumab in combination with nivolumab in EBV-associated advanced nasopharyngeal carcinoma (NCT03097939). Cancer Res.

[405] Schöffski P, Tan DSW, Martín M (2022). Phase I/II study of the LAG-3 inhibitor ieramilimab (LAG525) ± anti-PD-1 spartalizumab (PDR001) in patients with advanced malignancies. J Immunother Cancer.

[406] Fang W, Yang Y, Ma Y (2018). Camrelizumab (SHR-1210) alone or in combination with gemcitabine plus cisplatin for nasopharyngeal carcinoma: results from two single-arm, phase 1 trials. Lancet Oncol.

[407] Mai HQ, Chen QY, Chen D (2021). Toripalimab or placebo plus chemotherapy as first-line treatment in advanced nasopharyngeal carcinoma: a multicenter randomized phase 3 trial. Nat Med.

[408] Johnson D, Ma BBY (2021). Targeting the PD-1/PD-L1 interaction in nasopharyngeal carcinoma. Oral Oncol.

[409] Masterson L, Howard J, Gonzalez-Cruz J (2020). Immune checkpoint inhibitors in advanced nasopharyngeal carcinoma: beyond an era of chemoradiation?. Int J Cancer.

[410] Ding X, Zhang WJ, You R (2023). Camrelizumab plus apatinib in patients with recurrent or metastatic nasopharyngeal carcinoma: an open-label, single-arm, phase II study. J Clin Oncol.

[411] Tan EW, Abd-Aziz N, Poh CL (2022). Engineered oncolytic adenoviruses: an emerging approach for cancer therapy. Pathogens.

[412] Wen L, You C, Lu X (2015). Phase II trial of concurrent chemoradiotherapy with S-1 versus weekly cisplatin for locoregionally advanced nasopharyngeal carcinoma. Mol Clin Oncol.

[413] Gao Y, Liu Z, Liu Y (2021). Cisplatin combined with capecitabine-induced chemotherapy for local nasopharyngeal carcinoma can improve the quality of life and reduce toxic and side effects. World J Surg Oncol.

[414] Osterlund P, Kinos S, Pfeiffer P (2022). Continuation of fluoropyrimidine treatment with S-1 after cardiotoxicity on capecitabine- or 5-fluorouracil-based therapy in patients with solid tumours: a multicentre retrospective observational cohort study. ESMO Open.

[415] Kwakman JJM, Simkens LHJ, van Rooijen JM (2017). Randomized phase III trial of S-1 versus capecitabine in the first-line treatment of metastatic colorectal cancer: SALTO study by the Dutch Colorectal Cancer Group. Ann Oncol.

[416] Hu W, Wang W, Yang P (2015). Phase I study of icotinib, an EGFR tyrosine kinase inhibitor combined with IMRT in nasopharyngeal carcinoma. Int J Clin Exp Med.

[417] Chan ATC, Hsu MM, Goh BC (2005). Multicenter, phase II study of cetuximab in combination with carboplatin in patients with recurrent or metastatic nasopharyngeal carcinoma. J Clin Oncol.

[418] Wu LR, Zhu HF, Xu J (2018). Effectiveness of cetuximab in combination with concurrent chemoradiotherapy in locoregionally advanced nasopharyngeal carcinoma: a 1:2 propensity score-matched analysis. J Cancer.

[419] Zhang M, Huang H, Li X (2020). Long-term survival of patients with chemotherapy-naïve metastatic nasopharyngeal carcinoma receiving cetuximab plus docetaxel and cisplatin regimen. Front Oncol.

[420] Song X, Wang S, Li J (2021). Induction chemotherapy plus nimotuzumab followed by concurrent chemoradiotherapy for advanced nasopharyngeal carcinoma. Arch Med Sci.

[421] Kong L, Lin Q, Hu C (2016). Radiation plus concurrent nimotuzumab versus CDDP in locally advanced nasopharyngeal cancer: results of a phase III randomised trial. J Clin Oncol.

[422] Chen Q, Tang L, Liu N (2018). Famitinib in combination with concurrent chemoradiotherapy in patients with locoregionally advanced nasopharyngeal carcinoma: a phase 1, open-label, dose-escalation study. Cancer Commun.

[423] Hui EP, Ma BBY, Loong HHF (2018). Efficacy, safety, and pharmacokinetics of axitinib in nasopharyngeal carcinoma: a preclinical and phase II correlative study. Clin Cancer Res.

[424] Guan Y, Li A, Xiao W (2015). The efficacy and safety of Endostar combined with chemoradiotherapy for patients with advanced, locally recurrent nasopharyngeal carcinoma. Oncotarget.

[425] Chen C, Liu SR, Zhou S (2019). Recombined humanized endostatin (Endostar) intravenous infusion in the treatment of refractory nasopharyngeal carcinoma: three case reports. Medicine.

[426] Holliday EB, Frank SJ (2016). Proton therapy for nasopharyngeal carcinoma. Chin Clin Oncol.

[427] Dionisi F, Croci S, Giacomelli I (2019). Clinical results of proton therapy reirradiation for recurrent nasopharyngeal carcinoma. Acta Oncol.

[428] Williams VM, Sasidharan B, Aljabab S (2019). Proton radiotherapy for locally advanced nasopharyngeal carcinoma: early clinical outcomes from a single institution. Int J Radiat Oncol.

[429] Li X, Kitpanit S, Lee A (2021). Toxicity profiles and survival outcomes among patients with nonmetastatic nasopharyngeal carcinoma treated with intensity-modulated proton therapy vs intensity-modulated radiation therapy. JAMA Netw Open.

[430] Ma Y, Fang W, Zhang Y (2019). A phase I/II open-label study of nivolumab in previously treated advanced or recurrent nasopharyngeal carcinoma and other solid tumors. Oncologist.

[431] Sato H, Fushimi C, Okada T (2020). Investigation of the efficacy and safety of nivolumab in recurrent and metastatic nasopharyngeal carcinoma. In Vivo.

[432] Kao H-F, Ang M-K, Ng QS (2020). 266O combination ipilimumab and nivolumab in recurrent/metastatic nasopharyngeal carcinoma (R/M NPC): updated efficacy and safety analysis of NCT03097939. Ann Oncol.

[433] Xu R, Mai H-Q, Chen Q-Y (2021). JUPITER-02: randomized, double-blind, phase III study of toripalimab or placebo plus gemcitabine and cisplatin as first-line treatment for recurrent or metastatic nasopharyngeal carcinoma (NPC). J Clin Oncol.

[434] Yang Y, Qu S, Li J (2021). Camrelizumab versus placebo in combination with gemcitabine and cisplatin as first-line treatment for recurrent or metastatic nasopharyngeal carcinoma (CAPTAIN-1st): a multicentre, randomised, double-blind, phase 3 trial. Lancet Oncol.

[435] Smith C, McGrath M, Neller MA (2021). Complete response to PD-1 blockade following EBV-specific T-cell therapy in metastatic nasopharyngeal carcinoma. NPJ Precis Oncol.

[436] Choi J, Lee SY (2020). Clinical characteristics and treatment of immune-related adverse events of immune checkpoint inhibitors. Immune Netw.

[437] De Sousa LA, Battin C, Jutz S (2019). Therapeutic PD-L1 antibodies are more effective than PD-1 antibodies in blocking PD-1/PD-L1 signaling. Sci Rep.

[438] Naithani N, Sinha S, Misra P (2021). Precision medicine: concept and tools. Med J Armed Forces India.

[439] Koopman B, Groen HJM, Ligtenberg MJL (2021). Multicenter comparison of molecular tumor boards in The Netherlands: definition, composition, methods, and targeted therapy recommendations. Oncologist.

[440] Koopman B, van der Wekken AJ, Ter Elst A (2020). Relevance and effectiveness of molecular tumor board recommendations for patients with non-small-cell lung cancer with rare or complex mutational profiles. JCO Precis Oncol.

[441] Tamborero D, Dienstmann R, Rachid MH (2022). The molecular tumor board portal supports clinical decisions and automated reporting for precision oncology. Nat Cancer.

[442] Ahmed L, Constantinidou A, Chatzittofis A (2023). Patients’ perspectives related to ethical issues and risks in precision medicine: a systematic review. Front Med.

[443] Lehmann LS, Sulmasy LS, Burke W (2022). Ethical considerations in precision medicine and genetic testing in internal medicine practice: a position paper from the American College of Physicians. Ann Internal Med.

[444] Gavan SP, Thompson AJ, Payne K (2018). The economic case for precision medicine. Expert Rev Precis Med Drug Dev.

[445] Lu CY, Terry V, Thomas DM (2023). Precision medicine: affording the successes of science. npj Precis Oncol.

